# Metabolic partitioning between photosynthesis and osmotrophy in Collodaria photosymbiotic holobionts

**DOI:** 10.1038/s41467-026-76549-6

**Published:** 2026-08-19

**Authors:** Vera Nikitashina, Georg Pohnert

**Affiliations:** 1 Institute for Inorganic and Analytical Chemistry, Friedrich Schiller University Jena, Jena, Germany; 2 Cluster of Excellence Balance of the Microverse, Friedrich Schiller University Jena, Jena, Germany

**Keywords:** Marine biology, Marine chemistry, Symbiosis, Metabolomics

## Abstract

Collodaria (Radiolaria) are widespread marine planktonic protists that host photosynthetic microalgal symbionts. Within this photosymbiosis, they can contribute substantially to primary production, particularly in oligotrophic ocean regions. Despite their ecological importance, the mechanisms underlying the formation and maintenance of photosymbiosis are poorly understood. To uncover the metabolic dependencies within this photosymbiotic system, we develop a stable isotope labeling strategy that allows us to investigate the assimilation of inorganic and organic carbon in the Collodaria holobiont. By integrating metabolomic profiling with isotopic labeling analyses, we demonstrate that algal photosynthesis makes a substantial contribution to the holobiont carbohydrate pool. In particular, we observe the incorporation of inorganic carbon into glucose and fructose, followed by the enrichment of other metabolites including ribose. In contrast, osmotrophic uptake of dissolved organic compounds derived from labeled algal lysate does not lead to the incorporation of any of these carbohydrates, suggesting no significant contribution of osmotrophy to the holobiont’s carbohydrate pool. In contrast, specialized metabolites, such as dimethylsulfoniopropionate (DMSP) and betaines, are primarily acquired through osmotrophy implying a minor contribution of photosymbionts. Our findings thus reveal defined roles of phototrophic and osmotrophic metabolism in the Collodaria holobiont with consequences for our understanding of element fluxes in plankton.

## Abstract (web summary)

Collodaria harboring photosymbiotic microalgae are major primary producers in oligotrophic oceans. Stable-isotope labelling revealed distinct roles of phototrophy and osmotrophy, refining our understanding of plankton element-flux.

## Introduction

Phototrophic microorganisms inhabiting the upper layers of the water column form the foundation of the marine food web and contribute significantly to the global carbon cycle via photosynthesis^1^. Marine phototrophs can exist as free-living organisms or form symbiotic associations with heterotrophic community members—a relationship known as photosymbiosis. The photosymbiotic lifestyle is widely distributed among, for example, corals, sponges, mollusks, and jellyfish^2,3^. Also, various unicellular protists, including dinoflagellates, haptophytes, diatoms, foraminifera, and radiolaria, form photosymbiotic associations with different prokaryotic and eukaryotic microalgae^4,5^. Despite their substantial role in primary production and the marine food web, the mechanisms governing photosymbiotic relationships remain poorly understood. This hampers our ability to reliably understand and predict the functioning of marine ecosystems and the impacts of climate change. The urgent need to better investigate the marine ecosystems and their biodiversity has been highlighted by many international initiatives, such as the 2025 United Nations Ocean Conference.

The general model of photosymbiosis suggests that photosymbionts provide organic compounds, fueled by photosynthesis, to an otherwise heterotrophic partner, thereby making such associations mixotrophic^2,6^. The proportion of the photosymbionts’ contribution to the host’s nutrition varies among photosymbiotic systems and depends on environmental conditions. For example, in some sea slugs with acquired photosynthetic plastids, the photosynthesis provides less than 1% of the total organic carbon, whereas in some pelagic foraminifera, ciliates, corals, and jellyfish, the photosynthetic activity of symbionts can almost fully meet the carbon requirements^2,3,7–9^. The form of the translocated photosynthetic carbon includes diverse metabolites like lipids in foraminifera, sugars in ciliates, glycerol, lipids, and glucose in corals, or carbohydrates, fatty acids, and amino acids in jellyfish^3,5,8–14^. However, the role of the photosymbiont in photosymbioses of planktonic radiolaria remains poorly understood.

Planktonic radiolaria are widely distributed in the ocean and abundant in oligotrophic waters, forming a substantial fraction of the zooplankton^15,16^. Radiolaria feed heterotrophically on small zooplankton, microorganisms, and detritus. Many planktonic radiolaria harbor photosymbiotic microalgae and are therefore considered as mixotrophs^17–23^.

**Fig. 1:**
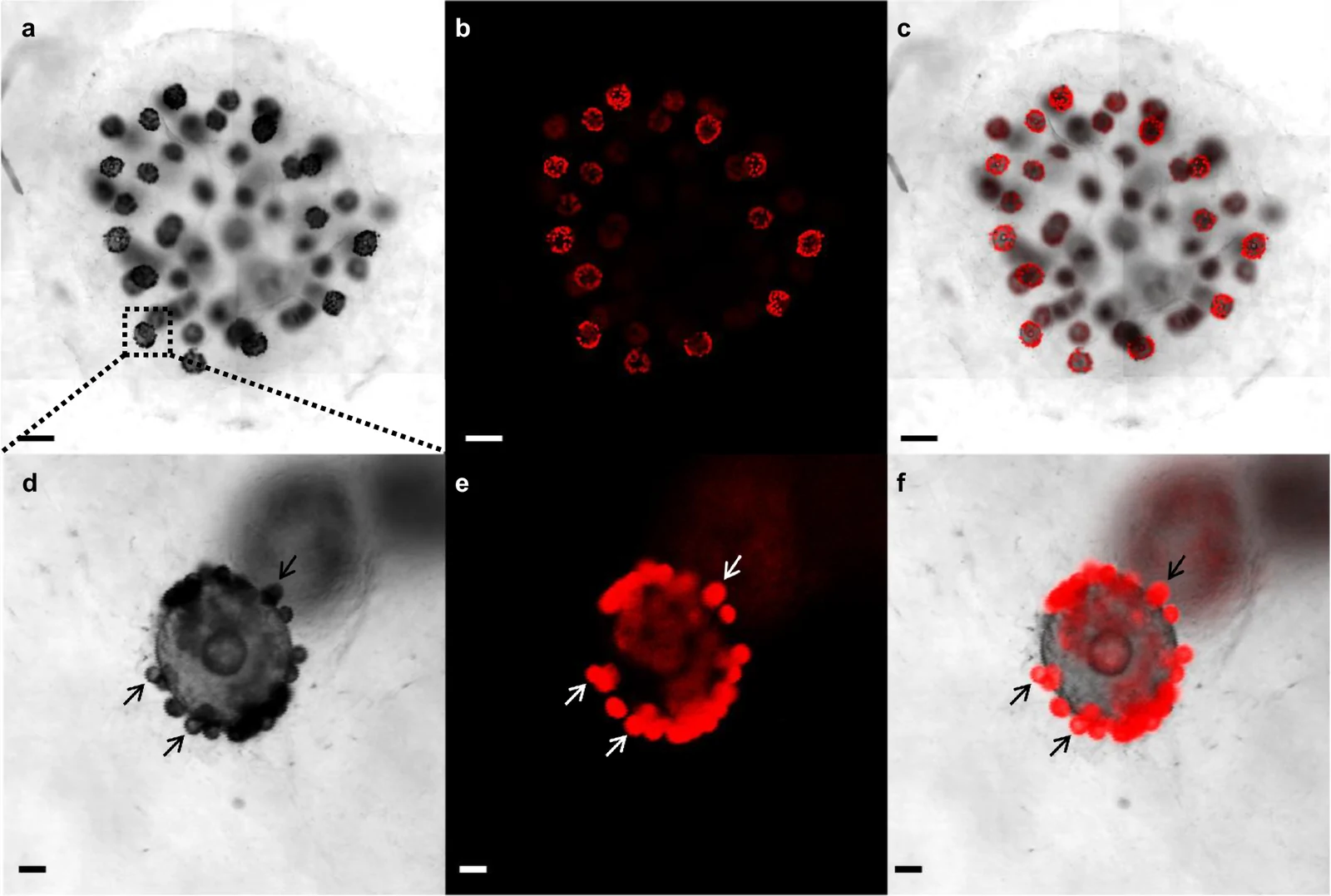
Colony of symbiont-bearing Collodaria (a-c) and collodarian central capsules at higher magnification (d-f) under a confocal microscope. **a,** **d** Transmitted light; **b,** **e** chlorophyll *a* autofluorescence; **c,** **f** overlay. The arrows point to single algal cells; scale bars represent 200 µm for **a**–**c**, 20 µm for **d**–**f**. Similar results were observed for *n* = 3 biological replicates, presence of microscopic algae was verified for all organisms investigated in this study.

Collodaria is an order of Radiolaria (subgroup of Polycystinea) that includes photosymbiont-bearing species. Indeed, all described colony-forming Collodaria host photosymbiotic algae in their cytoplasm^5,24,25^. Photosymbiotic Collodaria are the most abundant radiolaria in the upper 100 m of the water column^16^ and can harbor hundreds to thousands of microalgae in their cytoplasm (Fig. 1). Because the population density of microalgae is higher within the host compared to the surrounding environment, photosymbiont-bearing Collodaria represent hot spots of primary production^17,18,26,27^. Accordingly, Collodaria with their associated algae contribute to oxygen production, fixation of inorganic carbon, and its transport to higher trophic levels or the deep ocean^16,18,27^. The mechanisms of metabolite fluxes within Collodaria photosymbiotic associations and the role of symbionts are still not fully understood.

For Collodaria, the presence of the symbionts is believed to be obligate^21^. Some studies indicate that symbionts play a substantial role in meeting up to 80% of the daily carbon requirements in solitary and around 20% in colonial species^21,27^. But this substantial contribution is under debate, since another study estimates that the amount of photosynthetically derived carbon is less than 1% in photosymbiotic colonies, which is clearly insufficient to meet the colony’s nutrient needs^18^. In this case, the role of symbionts would lie rather in the supply of essential vitamins and/or protective secondary metabolites to the host^18^. Since the forms of translocated products are still undefined, a final clarification is not possible. An earlier study^28^ detected ^14^C-bicarbonate incorporation in lipids and aqueous soluble fractions of Collodaria extracts. Among labeled lipids, fatty acids, triglycerides, and wax esters were identified, but other, more polar metabolites were not addressed^28^. This finding could not be confirmed in a recent NanoSIMS study of labeled Collodaria colonies that did not detect label incorporation in any host organelles involved in lipid metabolism^26^. A transcriptomic analysis revealed a downregulation of symbiont genes involved in the glycolysis/gluconeogenesis pathway and lipid biosynthesis *in hospite*, also suggesting that these symbionts’ pathways are not the major sources of translocated organic carbon to the host^29^. The insights provided by radioisotope labeling and NanoSIMS approaches are limited to monitoring isotope shuttling but do not give any structural information about the metabolites involved in the translocation^10,26–28,30–33^.

Together with photosynthate translocation, metabolism of specialized metabolites (i.e., compounds that are not directly required for basic growth, development, or reproduction, but instead serve ecological and adaptive functions) can play a fundamental role in the holobiont-functioning. In this context, radiolaria photosymbioses are potentially important sources of marine dimethylsulfoniopropionate (DMSP)^34,35^. As a sulfur-containing compound and a precursor of the volatile dimethyl sulfide, DMSP contributes significantly to the global sulfur cycle and is primarily produced by marine phytoplankton and pelagic bacteria^36–39^. It serves as an osmoprotectant, an antioxidant, and signaling molecules^40–42^. In radiolarian photosymbioses, a significantly elevated DMSP content compared to free-living algae has been reported; however, the relative contribution of each symbiotic partner to the DMSP pool remains unclear^34,35^.

Dissolved organic compounds represent a major reservoir of organic carbon in the ocean^43^. They are passively or actively released by phytoplankton and play an essential role in global carbon cycling, being a primary carbon source for marine pelagic prokaryotes^43^. Furthermore, various protists are capable of uptake and consumption of dissolved organic compounds in a process termed osmotrophy^5,44–48^. Osmotrophy has been mainly studied in phytoplankton^49–52^, but has not yet been observed for Collodaria^53^.

Here, we address the origin of primary as well as specialized metabolites in radiolarian photosymbiosis using mass spectrometry-based analytical tools in combination with the administration of stable, non-radioactive isotopes. This method makes it possible to investigate both the structure and the origin of the metabolites involved in biochemical processes^54^. Introduction of one or more stable ^13^C isotope atoms into a metabolite increases the intensity of the heavier isotopologues (molecules that differ only in the isotopic composition and thus also in their molecular weight) relative to the naturally occurring compound^54^. Differences in the isotopologue distribution can be detected using high-resolution mass spectrometry (HRMS), and with these data, the degree of labeling (DOL) of each exchanged metabolite can be determined^54,55^. Incubations with labeled inorganic and organic carbon sources thus allow us to distinguish between phototrophic and osmotrophic metabolite acquisition in the colonial Collodaria photosymbiosis in the absence of phagotrophy. By analyzing ^13^C incorporation into the holobiont’s endometabolome (the complete set of low-molecular-weight metabolites within the holobiont), we identify the specific metabolites involved in the photosymbiosis and determine the contribution of photosynthesis and osmotrophy to metabolite acquisition of the holobiont. We show that photosynthetically assimilated carbon accounts for approximately one-third of the saccharide pool in the Collodaria holobiont within 12 h, whereas specialized metabolites such as DMSP and betaines are derived from osmotrophic uptake.

## Results

Using four different approaches for stable isotope labeling on freshly collected photosymbiont-bearing Collodaria (holobiont) (Fig. 1) in combination with metabolomics protocols, we aimed to elucidate the contribution of the respective partners to the holobiont metabolism. We therefore conducted (i) incubations with ^13^C-bicarbonate in the light to determine which fraction of metabolites is derived from photosynthetically fixed carbon. (ii) Incubations in a light/dark regime to monitor consumption and turnover of photosynthetically acquired metabolites in the holobiont, (iii) incubations under prolonged illumination to investigate the accumulation of the photosynthate in the holobiont, and (iv) incubation with labeled dissolved organic metabolites from algal lysate to monitor their osmotrophic uptake from the water.

**Fig. 2:**
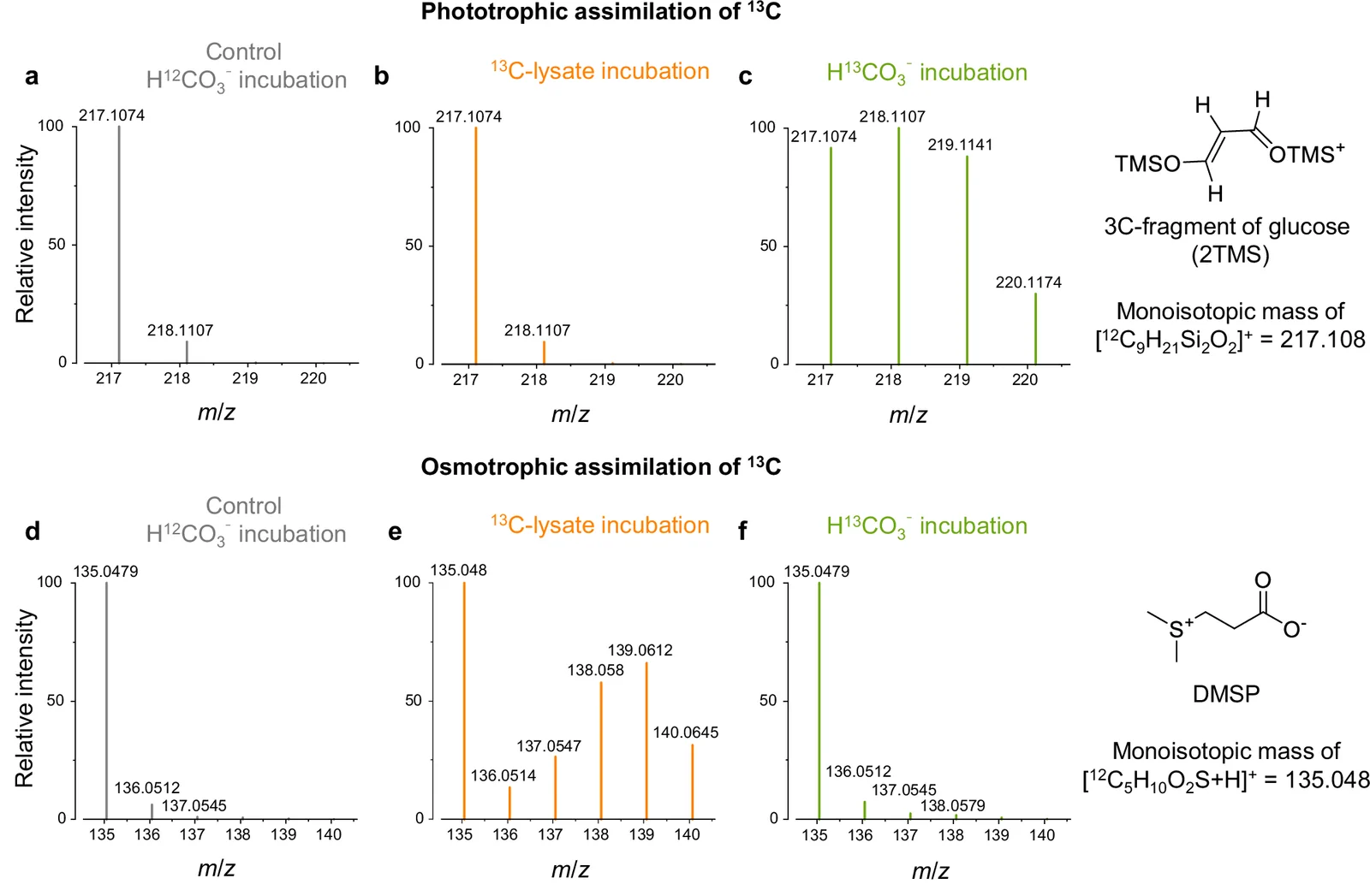
Representative mass spectra of ^13^C-enriched metabolites of the Collodaria holobiont. Incubation with ^13^C-enriched algal lysate resulted in no detectable ^13^C incorporation in glucose (**b**) but substantial incorporation into dimethylsulfoniopropionate (DMSP) (**e**). Incubation with ^13^C-bicarbonate resulted in substantial ^13^C incorporation into glucose (**c**) and minor incorporation into DMSP (**f**). **a**, **d** Show the ^12^C-bicarbonate control; the ^12^C-lysate control yielded identical spectra to this control and therefore is not shown. **a**–**c** Display mass spectra of the depicted C3-fragment of derivatized glucose (*m/z* = 217.108) measured by GC-HRMS, **d**–**f** show M + H^+^ ion of DMSP (*m/z* = 135.048) measured by LC-HRMS. *y*-axes show relative intensity compared to the most abundant ion; *x*-axes show the mass to charge ratio (*m/z*). TMS trimethylsily.

The analysis of mass spectra obtained from the four treatments revealed that specific metabolites were significantly ^13^C-enriched in the respective treatments (Fig. 2). The ^13^C enrichment was evaluated based on the signal intensity distribution of isotopologues (see “Materials and Methods”). A metabolite is considered labeled when signal intensities of higher isotopologues are higher than those from metabolites in the unlabeled controls. To assess the contribution of photosynthetic and osmotrophic activity to the pool of these metabolites, we calculated their DOL. This value indicates which proportion of an analyzed metabolite’s molecules contains an introduced stable isotope label.

This approach works without further data treatment with liquid chromatography high-resolution mass spectrometry (LC-HRMS) data. In gas chromatography, high-resolution mass spectrometry (GC-HRMS) using chemical ionization (CI) mode, it was not always possible to detect the molecular ion to evaluate the DOL directly. Therefore, we additionally determined the DOL by electron ionization (EI) (Supplementary Fig. 1). The DOL was similar in both methods, which allowed us to use fragment ions recorded in GC-EI MS and pseudo-molecular ions in LC-electrospray-HRMS to analyze DOL across all incubation setups.

### Photosynthetic contribution of symbiotic algae to Collodaria holobiont metabolism

**Fig. 3:**
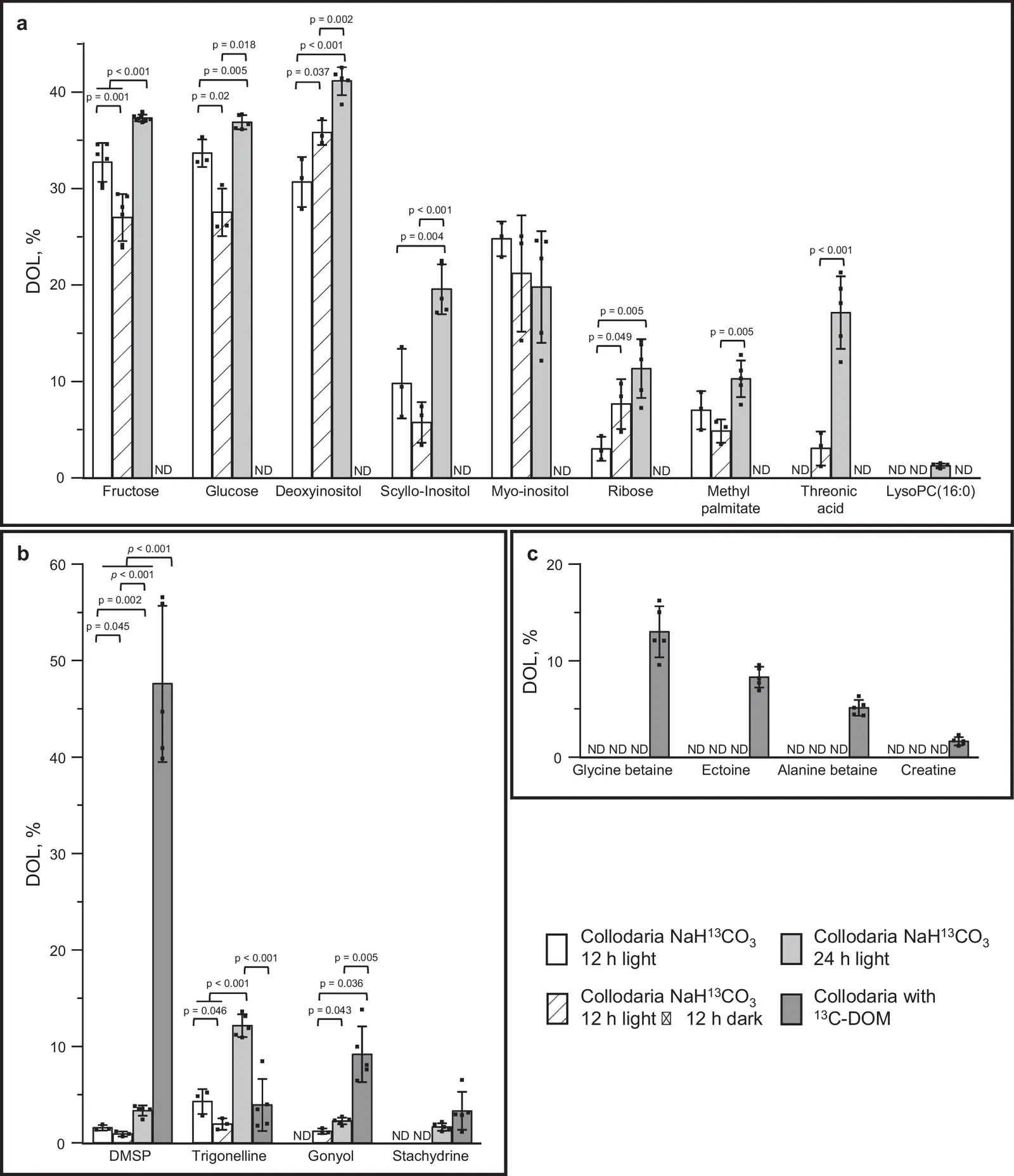
Degree of labeling (DOL) for metabolites of the Collodaria holobiont following incubation with ^13^C-bicarbonate or ^13^C-labeled dissolved organic matter (DOM) derived from algal lysate. **a** Metabolites incorporating ^13^C exclusively through photosynthesis, **b** metabolites incorporating ^13^C through both, photosynthesis and osmotrophy, and **c** metabolites incorporating ^13^C labeling exclusively through osmotrophy. The final ^13^C-DOL values were corrected for natural ^13^C abundance by normalization to the corresponding non-labeled control samples. Only metabolites whose identities were unambiguously confirmed using standards are shown, with the exception of deoxyinositol, which was identified based on a library match. Biological replicates were *n* = 3 for NaH^13^CO_3_ incubations under 12 h light and 12 h light/12 h dark, *n* = 5 for the 24 h light NaH^13^CO_3_ incubation, and for incubation with ^13^C labeled DOM; the same number of replicates was used for the corresponding non-labeled controls. Data are presented as mean ± SD. Statistical significance was assessed using an unpaired two-sided *t* test. LysoPC palmitoyl-glycero-phosphocholine, DMSP dimethylsulfoniopropionate; ND metabolite present, but no label incorporation detected. Source data are provided as a Source Data file.

The DOL in the holobiont’s metabolites incubated with ^13^C-bicarbonate showed that after 12 h in the light, around one-third of glucose and fructose and 30% of putatively identified deoxyinositol were produced through photosynthesis (Fig. 3a). Also, ^13^C-label incorporation was observed into myo-inositol, followed by methyl palmitate, trigonelline, scyllo-inositol, ribose, and, to a minor extent, DMSP. Overall, the substantial ^13^C incorporation in the Collodaria holobiont’s carbohydrates from dissolved bicarbonate suggests that the photosynthetic activity of symbiotic algae contributes significantly to the holobiont’s carbohydrate pool.

### Utilization of photosynthate

A 12 h dark period after incubation of the photosymbiont-bearing Collodaria with ^13^C-bicarbonate in the light (12 h) resulted in significantly lower DOL in both glucose and fructose compared to the incubation in the light alone (*p*-value ≤ 0.05), reaching approximately 27%. Similarly, the DOL of DMSP and trigonelline was significantly lower compared to the light alone treatment (Fig. 3). In contrast, the DOL of the putatively identified deoxyinositol and ribose significantly increased after incubation in the dark. For ribose, the proportion of labeled carbon increased more than twofold after dark incubations, and for deoxyinositol, the label incorporation increase made up around 15%. Incorporation of labeled carbon was also detected for threonic acid and gonyol, which were not labeled after 12 h of incubation in the light alone. The decrease in freshly photosynthetically derived carbon in both hexoses, coupled with its increase in deoxyinositol, ribose, threonic acid, and gonyol, indicates rapid metabolic turnover of the hexose pool in the dark. These patterns are consistent with hexoses being consumed and subsequently replenished by carbon sources independent of direct photosynthetic fixation—most likely through mobilization of previously stored compounds such as starch^56,57^.

### Prolonged incubations in the light

The analysis of ^13^C label incorporation into the metabolome of the Collodaria holobiont incubated for 24 h under light with ^13^C-bicarbonate showed the highest ^13^C incorporation rates for many analyzed metabolites, as expected (Fig. 3a, b). More than 35% fructose, glucose, and putatively identified deoxyinositol were derived from the carbon fixed during photosynthesis. The DOL of scyllo-inositol, trigonelline, and DMSP also significantly increased compared to both 12 h light and 12 h light/12 h dark incubations. The DOL of ribose was significantly higher in comparison to the 12 h light incubation. Whereas the DOL of methyl palmitate, gonyol, and threonic acid were higher compared to the light-dark incubation. Furthermore, 24 h incubation with labeled bicarbonate resulted in label detection in stachydrine and palmitoyl-glycero-phosphocholine that were not labeled in both 12 h light and 12 h light/12 h dark incubations. The increase in the proportion of photosynthetically derived carbon after prolonged (24 h) light incubation compared to shorter light incubation (12 h) suggests a direct relationship between symbionts’ photosynthetic activity and carbon incorporation in Collodaria holobiont metabolism.

### Contribution of osmotrophy to Collodaria metabolism

Besides analyzing the contribution of photosynthesis to the Collodaria holobiont metabolism, we also investigated ^13^C enrichment in metabolites that results from collodarian osmotrophy upon exposure to labeled dissolved organic metabolites. To obtain the labeled metabolites, which mimic compounds released from co-occurring microalgae in the field, we grew *Brandtodinium nutricula*—the free-living form of the Collodaria photosymbiont^25^ in ^13^C-bicarbonate-enriched medium and extracted the labeled metabolites that were used for further incubations from the algae. These compounds will only be a proxy for the metabolites the holobiont is exposed to, but include the most prevalent compound classes produced by phytoplankton. The final lysate concentration corresponded to algal cell densities that were approximately 10 times lower than the symbiont density within a colony^18,58^. Extraction success was >98%, calculated based on remains in the cell pellet for all investigated metabolites (Supplementary Fig. 2). The DOL for the ^13^C-enriched compounds of the lysate varied and, on average, made up around 60% (Supplementary Fig. 3). The DOL of the respective compounds in the lysate reflects the maximum possible labeling in the following incubations.

The analysis of DOL for Collodaria incubated with the ^13^C labeled algal lysate showed variable incorporation between different metabolites, with the highest value among identified metabolites for DMSP, reaching nearly 50%. The DOL of DMSP and gonyol were significantly higher in comparison to any of the incubations with labeled bicarbonate (Fig. 3b). In contrast, the DOL of trigonelline was significantly lower in colonies incubated with the labeled lysate compared to those incubated with labeled bicarbonate under light for 24 hours. We could also detect label incorporation in several metabolites that were not labeled after any performed incubation with ^13^C-labeled bicarbonate, such as ectoine, glycine-, and alanine betaines, and creatine (Fig. 3c). The exclusive label incorporation into these metabolites suggests their osmotrophic origin in the Collodaria holobiont in this experiment.

We also evaluated the DOL in carbohydrates, which were labeled after incubation with ^13^C-bicarbonate. However, no ^13^C incorporation was detected in these metabolites (Fig. 3a). These results suggest that osmotrophy does not substantially contribute to the availability of the identified sugars in Collodaria.

We addressed the contribution of photosynthetic activity of the symbionts and of osmotrophy of the host to the pool of metabolites shared by the Collodaria holobiont and the free-living form of the algae (Supplementary Table 1) by analyzing their DOL in each incubation. The investigated metabolites were identified in a previous study that compared the metabolic composition of the holobiont and the free-living algae^59^. More than half of the targeted metabolites, and particularly all analyzed amino acids, were not significantly labeled after incubation with neither labeled bicarbonate, nor labeled lysate, although we could detect ^13^C incorporation in the algal lysate itself (Supplementary Fig. 3). These results imply slow biosynthesis of these metabolites in the holobiont, such that incubation times used were not sufficient for accumulation of newly synthesized metabolites in detectable amounts neither by the photosymbionts nor by the host. Alternatively, the lack of ^13^C incorporation into these metabolites following incubation with labeled bicarbonate may reflect their rapid turnover by the host or symbiosis-induced modifications of algal metabolic pathways that suppress their production.

## Discussion

**Fig. 4:**
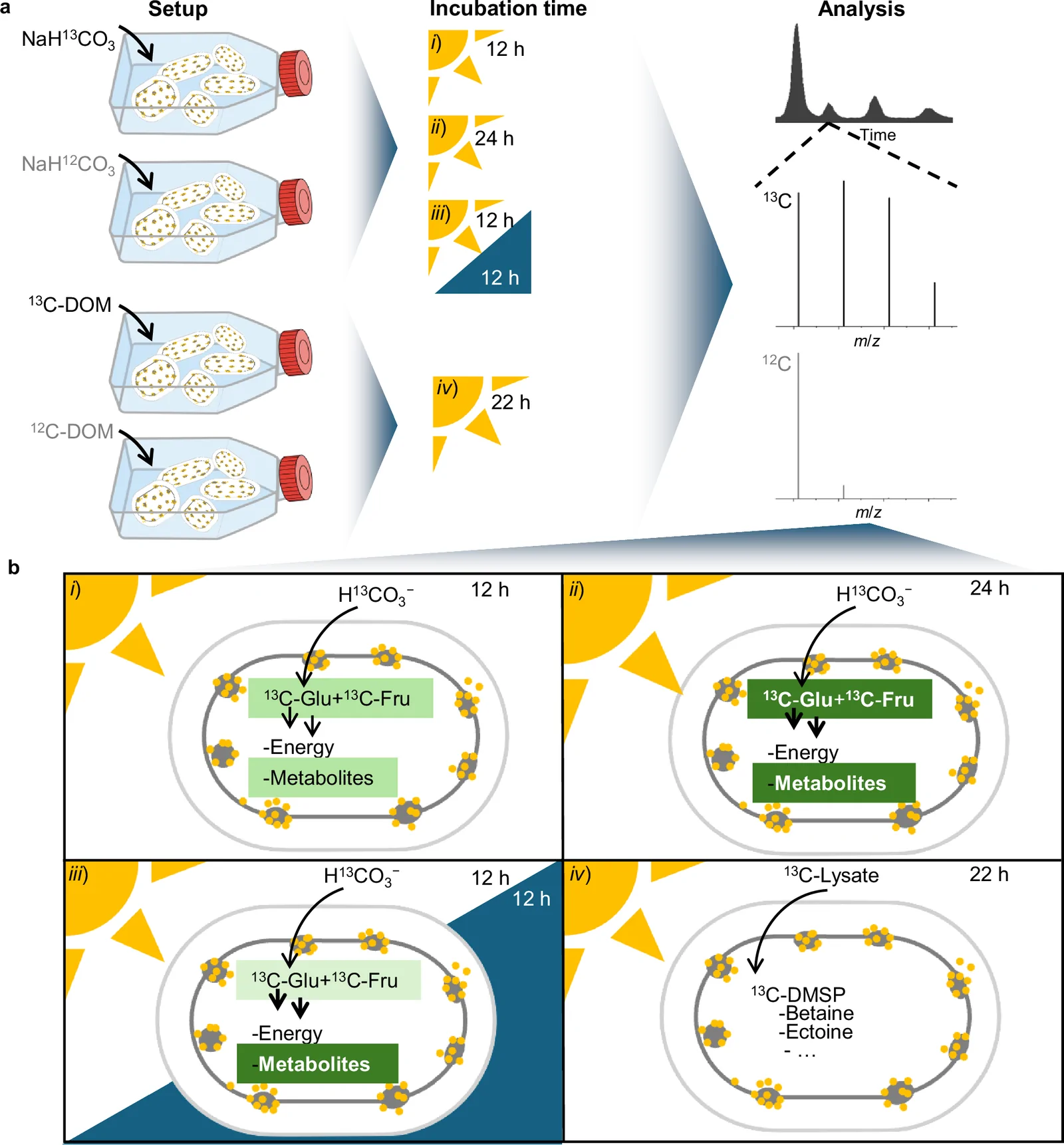
Experimental setup for ^13^C labeling and proposed metabolic fluxes in photosymbiont-bearing Collodaria. **a** Degree of ^13^C labeling of holobiont metabolites after incubation with ^13^C-labeled bicarbonate under different light conditions (i–iii) or with ^13^C-labeled dissolved organic matter (DOM) (iv), used to infer metabolite fluxes. **b** Conceptual model of carbon assimilation pathways: (i) incubation under light for 12 h with ^13^C-labeled bicarbonate leads to photosynthetic incorporation of inorganic carbon into glucose (Glu) and fructose (Fru), which are subsequently used for energy production and biosynthesis of downstream metabolites; (ii) prolonged light exposure (24 h) results in increased labeling of Glu, Fru, and derived metabolites; (iii) a 12 h dark period following 12 h of light leads to consumption of newly photosynthetically assimilated Glu and Fru and accumulation of their downstream metabolites; (iv) incubation with ^13^C-labeled algal lysate for 22 h results in assimilation of osmolytes, including dimethylsulfoniopropionate (DMSP), ectoine, and several betaines. Grey font represents ^12^C controls, black arrows indicate metabolite fluxes, green boxes indicate photosynthetically assimilated metabolites, dark green boxes indicate increased degree of labeling (DOL); light green box indicates decrease of DOL.

The absence of photosynthetically active algal symbionts significantly reduces the survival of the photosymbiont-bearing Collodaria^21^. However, because Collodaria are uncultivable, it is still not fully understood how the photosynthetic symbionts support them^5^. Here, we describe a set of molecules that connect the partners in this symbiotic system by different pathways. Overall, our analysis of the DOL of the Collodaria holobiont metabolome upon incubation with labeled bicarbonate or labeled dissolved organic compounds derived from algal lysate showed that the holobiont produces glucose and fructose during photosynthesis (Fig. 4). During the dark period, both sugars were converted to other metabolites, and their pool was being replenished from sources other than direct photosynthetic fixation. Prolonged incubation with labeled bicarbonate in the light resulted in DOL-increase in glucose, fructose, and derived metabolites. Osmotrophy served as a source of osmolytes but not sugars or amino acids. It has to be considered that all experiments were conducted in the absence of phagotrophy that might provide additional resources.

We observed that a substantial portion of glucose and fructose in the Collodaria holobiont was ^13^C-enriched after incubation with labeled bicarbonate. An accumulation of photosynthetically derived glucose by the host has been previously reported for various photosymbiont-bearing corals, jellyfish, and giant clams based on evaluations of ^13^C or ^14^C incorporation into host tissues after incubation with labeled bicarbonate^13,33,60–62^. Assimilation of photosynthetically derived fructose was previously reported for a photosymbiotic sea anemone^33^. Based on the earlier observations of substantial ^14^C label incorporation in the aqueous-soluble fraction in central capsules of photosymbiont-bearing Collodaria^28^, we conclude that in Collodaria photosymbiosis, photosynthetically assimilated glucose and fructose are metabolites that photosymbionts provide to the host.

Together with glucose and fructose, we also observed a substantial DOL for putatively identified deoxyinositol, and myo- and scyllo-inositols upon incubation with labeled bicarbonate (Fig. 3). Inositols are cyclic sugar alcohols found in eukaryotic cells, including marine microalgae and protists. They function as compatible solutes to protect cells from osmotic stress, and inositol phosphates mediate cellular signaling^63,64^. In photosymbiotic systems, photosynthetically derived inositols were previously detected in the tissues of photosymbiotic sea anemones, where the presence of myo-inositol depended on the species of the colonizing symbiont. The roles of these inositols, however, were not defined^33,60^. It was hypothesized that they play a role in host-photosymbiont signaling, recognition, and dysfunction, but no proof was documented^33,65^. Although observations suggest that signaling is active in many photosymbiotic associations, little is known about the involved chemistry. Among the identified signals in corals are glycans, oxylipins, reactive species, noncoding RNAs, and also secondary messengers associated with the phosphatidylinositol pathway^66,67^. The presence of photosynthetically-derived inositols in Collodaria photosymbiosis suggests a possible role in host-symbiont signaling. Drawn parallels provide a direction for further investigations in host-symbiont communication in marine photosymbiotic systems.

Assimilation of photosynthetically derived ^13^C carbon was also detected in methyl palmitate and palmitoyl-glycero-phosphocholine, indicating that these metabolites originate from photosynthetic activity of the microalgae as well. These results align with reports of Anderson and co-authors, who reported an incorporation of photosynthetically derived carbon into the lipid fraction of Collodaria, and particularly into fatty acids^28^. Translocation of the photosynthetically-derived fatty acids to the host was previously shown in jellyfish photosymbiosis^12^. Furthermore, accumulation of translocated symbiotic photosynthates in the form of lipid droplets has been observed in planktonic foraminifera with dinoflagellate photosymbionts^14^. Similarly, assimilation of photosynthetically derived carbon in lipid droplets was described for benthic foraminifera bearing photosynthetically active sequestrated algal chloroplasts^68^. Taken together, these observations suggest that lipids may be another universal class of compounds involved in metabolic interactions in photosymbiotic associations.

Investigating the dynamics of photosynthetically assimilated carbon incorporation, we observed a significant decrease in DOL of glucose and fructose after a 12 h light/12 h dark incubation. Together with the decrease of DOL for these two sugars, a significant increase of the DOL for ribose, putatively identified deoxyinositol, and threonic acid was observed (Fig. 3a). The increase in the DOL for these metabolites during the incubations suggests that they are produced from the sugars initially accumulated during photosynthesis. The phosphorylated form of ribose is produced from glucose via the pentose phosphate pathway and plays key roles in cellular energy metabolism. It also provides the carbon backbone for nucleotide and nucleic acid biosynthesis^69^. Its assimilation from photosynthetically derived glucose in Collodaria photosymbiosis implies a significant impact of photosynthetic activity for the holobiont´s energy metabolism. Threonic acid, in turn, is formed from ascorbic acid, which itself can be synthesized from fructose^70,71^. Photosynthetically derived threonic acid has been reported in coral photosymbioses^33^, and in photosymbiotic systems, it may be associated with coping with oxidative stress resulting from symbiont photosynthetic activity. There are a few reports on the biosynthesis and physiological role of putatively identified deoxyinositol. By drawing parallels with other inositols, it is possible that deoxyinositol is synthesized from glucose via myo-inositol and may serve osmolytic and antioxidative functions^70,72,73^.

In metabolomics studies, the total DOL is not accessible, but the DOL of the identified metabolites is recorded. Therefore, for now, it is impossible to say whether the decrease of ^13^C label for some metabolites during the dark period is associated with the conversion of labeled precursors to other metabolites, or whether they are consumed as an energy source, or if both mechanisms are taking place. An increase in label incorporation for some metabolites, such as ribose, threonic acid, gonyol, and deoxyinositol during the dark period, supports the first mechanism. The lack of substantial label incorporation in other compound classes, like amino acids (Supplementary Fig. 3), indicates that photosynthetically assimilated carbon is mainly used for respiration rather than cell anabolism. Although our approach did not allow us to distinguish between host- and symbiont-derived metabolites within the holobiont, the comparatively high proportion of labeled carbohydrates, and relatively small contribution of the photosymbionts to the holobiont biomass^18^, suggest that the detected carbohydrates were present in both the symbionts and the host. Therefore, based on our observations, we hypothesize the following Collodaria–photosymbiont metabolic interactions: During photosynthesis, algae assimilate carbon and transfer it to the host as glucose and fructose, which are predominantly used for respiration to generate energy. The remaining photosynthetically derived carbon is converted into deoxyinositol via myo-inositol, as well as scyllo-inositol, ribose, and threonic acid. Alternatively, inositols could be directly supplied by the symbiont to the host (Fig. 4). Both the conversion of photosynthetically-derived metabolites and their consumption (similar to our model) have been described in corals^32,74^, implying shared metabolic mechanisms underlying diverse marine photosymbioses. The proposed hypothesis represents a model for further studies on metabolite exchange within Collodaria photosymbiosis and metabolic fluxes in marine plankton ecosystems.

Dissolved organic compounds constitute a significant portion of organic carbon in the ocean, and their release by phytoplankton is high in oligotrophic waters^43^. Since various protists are capable of osmotrophy^5^, and since it has been suggested that the osmotrophy is ubiquitous among phyto- and microzooplankton^44,47^, we investigated the ability of the Collodaria holobiont to assimilate dissolved organic compounds from the surrounding environment. Our results suggest that the Collodaria exhibit a selective uptake of defined metabolites. Thus, we did not observe any label incorporation for carbohydrates and amino acids when incubated with labeled dissolved organic compounds. This fact supports our conclusions above that carbohydrates are primarily derived from photosynthetic activity, at least in the absence of phagotrophy.

For metabolites like alanine, betaine, glycine betaine, ectoine, and creatine, the absence of ^13^C incorporation from photosynthetic activity, combined with the label incorporation after incubation with labeled lysate, suggests that Collodaria can assimilate these compounds through osmotrophy. All metabolites in this category exhibit high polarity, which may be a prerequisite for uptake. They can also contain nitrogen or sulfur, suggesting that osmotrophic assimilation of these compounds could contribute to nitrogen and sulfur acquisition. However, the relative contribution of this pathway compared to phagotrophy requires further investigation.

For compounds such as DMSP, stachydrine, gonyol, and trigonelline, ^13^C incorporation was observed after incubation with both inorganic and organic labeled carbon sources. The relative contribution of the source varied for these metabolites. Thus, significantly higher contribution of osmotrophic uptake than inorganic carbon incorporation was observed for DMSP and gonyol, whereas trigonelline contained the highest portion of the label after 24 h of incubation in the light with ^13^C-bicarbonate.

DMSP plays an important role as an osmoprotectant, antioxidant, and signaling molecule in various marine microorganisms and coral photosymbiosis^40–42^. Although previously detected in higher concentrations in radiolaria photosymbioses than those calculated for free-living algae, its origin has never been determined. Our observation of a high proportion of labeled DMSP after incubation with labeled algal lysate, together with photosynthetic contribution to the pool of this compound suggest that Collodaria can obtain this metabolite from the environment and the photosymbionts. The dual origin of this molecule may explain its higher-than-expected concentration in Collodaria reported earlier^35^ and indicate Collodaria photosymbiosis as an important sink of environmental DMSP.

The absence of photosynthetically assimilated carbon in ectoine in Collodaria colonies, together with comparatively low DOL of this metabolite in the algal lysate (Supplementary Fig. 3) could be explained by the fact that ectoine is supplied by bacteria to both partners. Marine bacteria are known to produce ectoine and supply it to the microalgae^75,76^. Since the microbiome of Collodaria and its role have never been thoroughly described, its contribution of ectoine to Collodaria metabolism is possible and requires further investigation.

This study contributes to the still-limited knowledge on the metabolic interdependency between radiolaria and their photosymbionts. Our findings show that photosymbiont-bearing Collodaria extensively assimilate photosynthetically derived carbon, highlighting their role as primary producers in oligotrophic waters, where they are highly abundant^16^. Alongside this, considerable acquisition of specialized metabolites, such as ectoine, DMSP, gonyol, and betaines by means of osmotrophy stresses their contribution to the recycling of dissolved organic matter in the ocean. Through accumulation of high concentrations of DMSP, they contribute as a sink to metabolites of the global sulfur cycle, and their acquisition of ectoine, potentially from surrounding bacteria highlight their metabolic interconnection with marine microbial communities. Our results pave the way for further molecular, genomics, metabolomics, and transcriptomics studies to investigate the biosynthetic pathways of identified molecules and the contribution of surrounding microbiota to the Collodaria photosymbiotic association.

## Methods

### Solvents

For endometabolome extraction, LC-HRMS, GC-HRMS analyses, the following solvents were used: methanol (SupraSolv, Merck, Germany), ethanol (LiChrosolv, Supelco, Merck, Germany), chloroform (HPLC grade, Fisher Scientific, UK), acetonitrile (CHEMSOLUTE, Th. Geyer, Germany), water (Chromasolv Plus for HPLC, Honeywell, Germany) if not stated otherwise.

### Strains and culture conditions of the microalgae

A strain of *B. nutricula* RCC 3387 was obtained from the Roscoff Culture Collection (Roscoff, France). The strain was grown under sterile conditions, and the associated microbiota were not removed. The culture medium was artificial seawater^77^ supplemented with an L1 culture medium kit from the Bigelow National Center for Marine Algae and Microbiota (Maine, USA) and 3 g L^−1^ sea salts (Sigma-Aldrich, USA). To obtain the algae with ^13^C-enriched metabolome, the NaH^12^CO_3_ in the medium was replaced with NaH^13^CO_3_ (SIGMA-ALDRICH, USA). To maximize ^13^C label incorporation in the algal metabolome, at least three passages of cultivation were performed in NaH^13^CO_3_-containing medium^48^. The inoculation of the new cultures was performed from the stationary phase cultures grown in NaH^13^CO_3_-containing media. Starting cell density was 2.3 × 10^4^ cells mL^−1^. Control cultures were grown in normal medium. The culturing temperature was 18 °C, and the light was adjusted to 90–110 μmol photons m^−2^ s^−1^ with a 14/10 light/dark cycle.

### Preparation of algal lysate

For the preparation of algal lysate, *B. nutricula* was grown in 250 mL culture flasks with 265 mL of the ^13^C-enriched culture medium filled to the top of the rim to minimize the surface area where air exchange occurs. ^12^C control cultures were grown in 250 mL culture flasks containing 180 mL of culture medium. When the cultures reached the stationary phase, cells were harvested by centrifugation at 3000 × *g* at 18 °C for 5 min, washed and re-centrifuged three times with non-labeled medium, and frozen at –80 °C. To prepare the algal lysate, frozen samples were lyophilized, resuspended in the non-labeled medium, and sonicated for 10 min in an ultrasonic cleaner USC1200TH (VWR, Malaysia). The samples were then vortexed for 30 s, frozen at –80 °C, thawed, frozen again, and subsequently lyophilized and stored below –20 °C.

To check the efficiency of metabolite extraction from algal cells, algal lysate was dissolved in double-distilled, sterile H_2_O. Two replicates of 1 mL aliquots corresponding approximately to 4 × 10^6^ *B. nutricula* cells mL^−1^ were processed. The samples were centrifuged at 20,627 × *g* at room temperature for 15 min, supernatants were transferred into new 1.5 mL Eppendorf Safe-Lock tubes (Eppendorf AG, Germany), and both pellets and supernatants were dried under vacuum. Dried samples were resuspended in 200 µL of methanol:acetonitrile:water (5:9:1, *v*:*v*:*v*), vortexed 1 min, centrifuged at 20,627 × *g* at room temperature for 15 min two times, and the tubes were rotated by 180° between the centrifugation cycles. Supernatants were transferred into glass vials with inserts, 1:5 dilutions were prepared in duplicates, and the samples were measured on a SeQuant ZIC-HILIC column as described below.

### Collection of Collodaria

Access and Benefit-Sharing (ABS) authorization was obtained on 29 March 2023 from the Ministère de la Transition écologique et de la Cohésion des territoires, Direction générale de l’aménagement, du logement et de la nature, Direction de l’eau et de la biodiversité, Bureau de l’encadrement des impacts sur la biodiversité (Reference No. TREL2302365S/655; ABS-CH Unique Identifier: ABSCH-IRCC-FR-263703-1).

Collodaria were collected in the bay of Villefranche-sur-Mer (France, 43°41´10´´N, 7°18´50´´E) in March 2024 using a plankton net with a 200 µm mesh size. Samples were obtained by slowly towing the net just below the water subsurface.

The samples were diluted in buckets with freshly collected seawater from the same sampling site. Individual morphologically similar Collodaria colonies containing symbiotic algae were manually selected under a microscope and transferred into filtered seawater (FSW, 0.2 µm pore size) from the same location. Given the requirement for comparatively high quantities of colonies per sample in each experiment, precise identification of individual Collodaria colonies to the species level was not achievable.

After at least 1 h of incubation, Collodaria colonies were transferred to fresh FSW. This transfer protocol was repeated at least three times to remove attached particles. This and all subsequent incubations were performed at the same temperature as the ambient seawater (16 °C), with illumination set to 85–95 μmol photons m⁻² s⁻¹ with various light regimes (see below).

### Incubation of Collodaria

Cleaned colonies were incubated either with NaH^13^CO_3_-enriched medium or ^13^C-enriched *B. nutricula* lysate. Corresponding non-labeled (^12^C) controls were prepared and processed for each experiment. A minimum of three replicates were processed per treatment.

For incubation with ^13^C-bicarbonate, NaH^13^CO_3_ (SIGMA-ALDRICH, USA) was dissolved in FSW to a final concentration of 0.2 g L^−1^. For controls, a solution of NaH^12^CO_3_ (Merck KGaA, Germany) in FSW with the same final concentration was prepared. The incubation of Collodaria colonies was performed in either 6-well plates with 10 mL of FSW supplemented with NaH^12/13^CO_3_ per well (12 h incubation), or in 50 mL cell culture flasks containing 55 mL of FSW supplemented with NaH^12/13^CO_3_ per flask (24 h incubation). Cleaned colonies were randomly distributed among the wells when incubated in the well plates (two colonies per well), or among the flasks when incubated in the flasks (nine colonies per flask).

Three setups with different time regimes were performed:

i) Incubation for 12 h in the light,
ii) Incubation for 12 h in the light followed by 12 h in the darkness,
iii) Incubation for 24 h in the light.
  The incubations were terminated by transfer of all colonies with a pipette from the treatment into fresh FSW. For washing off residual labeled medium, these colonies were transferred two more times to fresh FSW. The colonies were checked for their integrity under the microscope, transferred into 1.5 mL Micro tubes (SARSTEDT AG &Co. KG, Germany) with a minimal amount of FSW, immediately frozen in liquid nitrogen, and stored below –70 °C until further processing. Each replicate contained seven to nine colonies; each replicate had the same number of colonies across one setup. For 12 h incubation under light and 12 h light/12 h dark incubation, three biological replicates were prepared; for the 24 h light NaH^13^CO_3_ incubation, 5 biological replicates were prepared; the same number of replicates was used for the corresponding non-labeled controls.
iv) Incubation with algal lysate
  For incubation with the algal lysate, lyophilized lysates of *B. nutricula* grown in either normal or ^13^C-labeled media as described above were used. The lyophilized lysates from two 250 mL cultures for each treatment were resuspended in 2.5 mL of double-distilled, sterile-filtered H_2_O and pooled together. One mL samples corresponding to approximately 3 × 10^6^ *B. nutricula* cells mL^−1^ were aliquoted for LC-/GC-HRMS analysis and transferred into 1.5 mL Eppendorf Safe-Lock tubes (Eppendorf AG, Germany), frozen in liquid nitrogen, and stored below –70 °C until further processing. Aliquots corresponding to the final concentration of 3.5 × 10^4^ cells mL^−1^ were added to FSW. This concentration is approximately ten times lower than the symbiont density within a colony^18,58^, and was chosen to avoid any potentially harmful effects of the lysate on the colony. Incubation of Collodaria colonies was performed in 50 mL cell culture flasks with 50 mL of algal lysate solution in the light. After 22 h, Collodaria colonies were transferred into fresh FSW without lysate. To ensure residual lysate was removed, the isolation and transfer were repeated three times. After a total time of 24 h, Collodaria colonies were checked for their integrity under microscopy, transferred into 5 mL Eppendorf tubes (Eppendorf AG, Germany) with a minimal amount of FSW, immediately frozen in liquid nitrogen, and stored below –70 °C until further processing. Each replicate contained eleven colonies, 5 biological replicates were prepared, and the same number of replicates was used for the corresponding non-labeled controls.

### Extraction LC-/GC-HRMS sample preparation

Before extraction, all samples were lyophilized. The extraction of the samples was performed according to a previously published method^59^. The samples evaporated under vacuum were kept under argon and stored below –20 °C.

Dry samples were dissolved in 80 µL of methanol:acetonitrile:water (5:9:1, *v*:*v*:*v*). For GC-HRMS analysis, 25 µL of each sample was transferred into vials, and 1 µL from each sample was combined to create the pooled quality-control (QC) sample. Samples and QC were subsequently dried under vacuum. Dried samples were dissolved in 35 µL and QC in 140 µL of pyridine (Chromasolv Plus, Sigma-Aldrich) containing 20 mg mL^−1^ methoxyamine monohydrochloride (Sigma-Aldrich). All samples were vortexed for 1 min, briefly spun down, sonicated in an ultrasonic cleaner for 10 min, vortexed for 1 min, briefly spun down again, heated at 60 °C for 1 h, vortexed 1 min, briefly spun down, and incubated at room temperature overnight. The pooled QC was split into 4 replicates. To each sample 35 μL of *N*,*O*-bis(trimethylsilyl)trifluoroacetamide (Macherey-Nagel) was added. To the QC samples, 1 μL of a C7–C40 alkane standard mix, 100 μg mL^−1^ in hexane (diluted 1:10 (*v*:*v*) from the 1 mg mL^−1^ standard from Sigma-Aldrich) was added. Samples were vortexed for 1 min, briefly spun down, ultrasonicated for 10 min, vortexed for 1 min, briefly spun down, heated at 60 °C for 1 h, vortexed 1 min, briefly spun down, ultrasonicated for 10 min, transferred to 1.5 mL Eppendorf Safe-Lock tubes, and centrifuged at 16,000 × *g* at 4 °C for 15 min. Supernatants were transferred into glass vials with inserts and directly measured.

For LC-HRMS analysis, 35 µL of each sample dissolved in methanol:acetonitrile:water (5:9:1, *v*:*v*:*v*) was transferred into vials with inserts and diluted 5.5 times with the same solvent mixture to measure on a SeQuant ZIC-HILIC column. An extract of *B. nutricula* equivalent to 10^7^ cells per sample was processed in the same way and used as a QC sample.

### GC-HRMS measurements

GC-HRMS measurements were performed on a Q-Exactive Orbitrap mass spectrometer connected to a Trace 1310 Gas Chromatograph with a TriPlus RSH Autosampler (Thermo Scientific, Bremen, Germany). The gas chromatograph was equipped with a Zebron ZB-SemiVolatiles column (30 m × 0.25 mm × 0.25 μm, Phenomenex, USA). Helium was used as a carrier gas with a flow rate of 1 mL min^−1^. Temperature of the injector was 250 °C, of the transfer line 250 °C, and of the auxiliary zone 280 °C. The oven program was as followed: the initial temperature of 80 °C was maintained for 2 min, rose to 120 °C at a rate of 20 °C/min, maintained for 1 min, rose to 250 °C at a rate of 5 °C/min, rose to 320 °C at a rate of 10 °C/min, and maintained for 2 min. For the electron ionization (EI) mode, the ion source temperature was 300 °C, and for the chemical ionization (CI) mode, the ion source was set to 180 °C. Methane was used as an ionization gas with a flow rate of 1.5 mL min^−1^. The injected volume of samples was 1 μL, for EI mode split ratio was 1 to 5, and in CI mode, samples were measured in the splitless mode. Data were recorded in full scan profile mode with a resolution set to 120,000 across a mass range from 50 to 600 m/z in EI mode, and mass range from 80 to 1000 m/z in CI mode; the ionization energy was 70 eV. Mass accuracy was <5 ppm. Data were acquired using Thermo Finnigan XCalibur 4.4.16.14.

### LC-HRMS measurements

Samples for LC-HRMS analysis were measured with a Dionex UltiMate 3000 system equipped with UltiMate WPS-3000RS autosampler and coupled to a Q-Exactive Plus Orbitrap mass spectrometer (Thermo Scientific, Bremen, Germany). The column compartment temperature was set to 25 °C. The injection volume of the samples was 1 µl. The auto-injector was not washed between samples, and the carryover of the samples was controlled by solvent blank measurements.

The chromatographic separation was performed on a SeQuant ZIC-HILIC column (5 μm, 200 Å, 150 × 2.1 mm, Merck, Germany), equipped with SeQuant ZIC-HILIC guard column (20 × 2.1 mm, Merck, Germany). The duration of the method was 14.5 min with an MS runtime from 0.5 to 9 min. Eluent A consisted of water with 2% acetonitrile and 0.1% formic acid, eluent B of 90% acetonitrile with 10% aqueous ammonium acetate (1 mmol L^−1^). The flow rate was set to 0.6 mL min^−1^, the gradient started with 85% solvent B, which was held for 4.0 min, gradient to 0% of solvent B (4.0–5.0 min), hold time 0% B (5.0–9.0 min), gradient to 100% of B (9.0–10.0 min), hold time 100% B (10.0–12.0 min), gradient to 85% of B (12.0–12.5 min), hold time 85% B (12.5–14.5 min).

The MS measurements were performed in positive mode, with a resolution set to 280,000 within a scan range from 80 to 1200 m/z. Heated electrospray ionization was used to generate molecular ions. Mass accuracy was <5 ppm. Data were acquired using Thermo Finnigan XCalibur 4.4.16.14.

### GC-HRMS data processing and analysis

Raw data obtained from GC-HRMS analysis with EI mode was converted into.mzXML format using the Proteowizard Suite (proteowizard.sourceforge.net) with vendor peak picking enabled. Data was processed with the R-based X13CMS package^78^ for isotopologues detection, the script can be found in the supplementary information (Supplementary Code 1). Afterwards, significantly labeled isotopologue groups (*p*-value <0.05) were manually curated, and only groups with at least three consecutive isotopologues were considered for further analysis. For compound identification, the same.mzXML files were processed with the previously established R-based pipeline^79^ with some modification (Supplementary Code 2). The pipeline includes the XCMS package^80^, CAMERA package^81^, and MetaMS package^82^. The significantly labeled features from the X13CMS analysis were matched with the features from the analysis with the second pipeline based on the same retention time and *m*/*z* value. Identification of metabolites was conducted using the NIST 2011 Mass Spectral Library database (National Institute of Standards and Technology, USA). The final verification of compounds was done by comparison of mass spectra and retention index (accepted difference ≤3 units) against mass spectra and retention index of analytical standards deposited in an in-house library.

The ^13^C incorporation rate was calculated using Eq. (1) based on the intensities of the isotopologues of molecular ions extracted from the GC-HRMS CI mass spectrum. Consecutive isotopologues were identified as peaks differing by 1.00335 Da (±5 ppm). Since molecular ions were not always detected, the ^13^C incorporation rate for sugars was also calculated using one of the most intense fragments—*m*/*z* 217.108^83^ in the EI mode. For inositols, the ^13^C incorporation rate was calculated for *m*/*z* 305.1424^84^ to account for potential uneven distribution of ^13^C-labeled atoms in the molecule (Supplementary Fig. 4). Compounds were considered significantly ^13^C-enriched if the difference between labeled and unlabeled compound averages was significant (*p* ≤ 0.05, unpaired *t* test). The final ^13^C incorporation rate was corrected for natural ^13^C abundance using Eq. (2) across all replicates.

1 $${DOL}_{u}=\frac{{\sum}_{m=0}^{n}\frac{m}{n}\cdot{I}(m)} {{\sum}_{m=0}^{n} {I}(m)}\cdot{100}\%$$

DOL_u_ – uncorrected degree of ^13^C labeling

*n* – total number of carbon atoms

*m* – number of isotopologues (starting from 0)

*I*(*m*) – intensity of each isotopologue

2 $${DOL}=\frac{{{DOL}}_{u}-{{DOL}}_{n}}{1-\frac{{{DOL}}_{n}}{100}}$$

DOL – degree of labeling

DOL_u_ – uncorrected degree of ^13^C labeling

DOL_n_ – average of natural ^13^C content for unlabeled sample (carbon from derivatization reagent excluded).

### LC-HRMS data processing and analysis

Raw data obtained from LC-HRMS analysis were preprocessed using Compound Discoverer version 3.3 SP3 (Thermo Fisher Scientific, USA). The standard workflow (Stable Isotope Labeling w Metabolika Pathways and ID using Local Databases) with default settings was applied. The setting for Peak Rating Filter was changed to: Peak Rating Threshold—5, number of files—3. Within this workflow, a grouping of isotopes and adducts belonging to one molecule is performed. All incubation experiments were processed within one study. The compound identification was performed with an in-house library based on retention time and MS1/MS2 spectra (accepted difference ≤0.2 min, <5 ppm). Compounds were considered significantly ^13^C-enriched if DOL values differed significantly (*p* ≤ 005, unpaired *t* test) between labeled and unlabeled spectra. The final ^13^C incorporation rate was normalized for natural ^13^C abundance using Eq. (2) across all replicates.

The depiction of GC- and LC-HRMS results was performed with OriginPro 2025 (OriginLab Corporation, USA).

### Microscopic images

Fluorescent images of Collodaria colony were taken with a SP8 Leica confocal microscope (excitation 638 nm, emission 645–671 nm). Images of six regions from one focus plane were taken and assembled to cover the whole colony.

### Reporting summary

Further information on research design is available in the Nature Portfolio Reporting Summary linked to this article.

## Supplementary information

Supplementary Information

Description of Additional Supplementary Files

Supplementary Code

Reporting Summary

Transparent Peer Review File

## Source data

Source Data

## Data Availability

The authors declare that all data supporting the findings of this study are available within the paper and its Supplementary files. The metabolomics data generated in this study have been deposited to MetaboLights^85^ repository with the study identifier MTBLS12592. Source data are provided with this paper.

## Code availability

Code used for data analysis can be found in Supplementary Code 1 and 2.
