# Supplementary material for: Metabolic partitioning between photosynthesis and osmotrophy in Collodaria photosymbiotic holobionts
Source: Nat Commun. 2026 Aug 19;17:8570. doi: 10.1038/s41467-026-76549-6 (PMC13490583; doi:10.1038/s41467-026-76549-6)
Supplement: Supplementary file 1 [file 41467_2026_76549_MOESM1_ESM.pdf]

Supplementary Information:

Metabolic Partitioning between Photosynthesis and Osmotrophy in Collodaria Photosymbiotic Holobionts

Vera Nikitashina[1] and Georg Pohnert[1,2*]

[1]Institute for Inorganic and Analytical Chemistry, Friedrich Schiller University Jena – Jena, Germany

[2]Cluster of Excellence Balance of the Microverse, Friedrich Schiller University Jena, Jena, Germany

*contact Georg Pohnert: Friedrich Schiller University Jena, Microverse Center Jena, Rosalind-Franklin-Str. 5, D-07745 Jena Germany; Georg.Pohnert@uni-jena.de

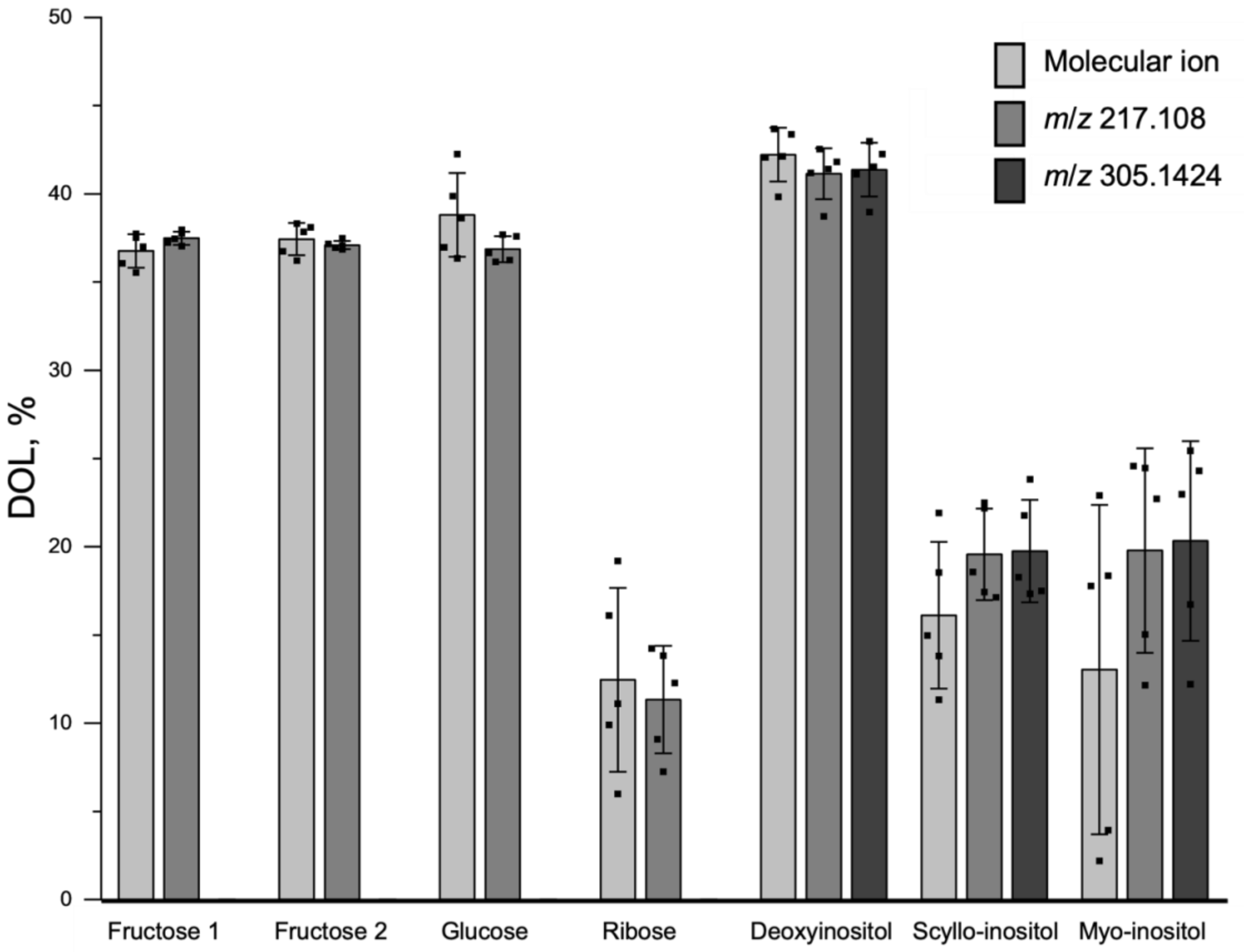

Supplementary Figure 1: Degree of labeling (DOL) of Collodaria holobiont metabolites calculated for molecular ion (chemical ionization mode), a fragment with *m/z* 217.108 (electron ionization mode (EI)), and *m/z* 305.1424 (EI). A comparison showed that the DOL determined from the fragments in EI mode was nearly identical to that obtained from the molecular ion. Biological replicates were n = 5, the same number of replicates was used for the corresponding non-labeled controls. Data are presented as mean ± SD. Source data are provided as a Source Data file.

a

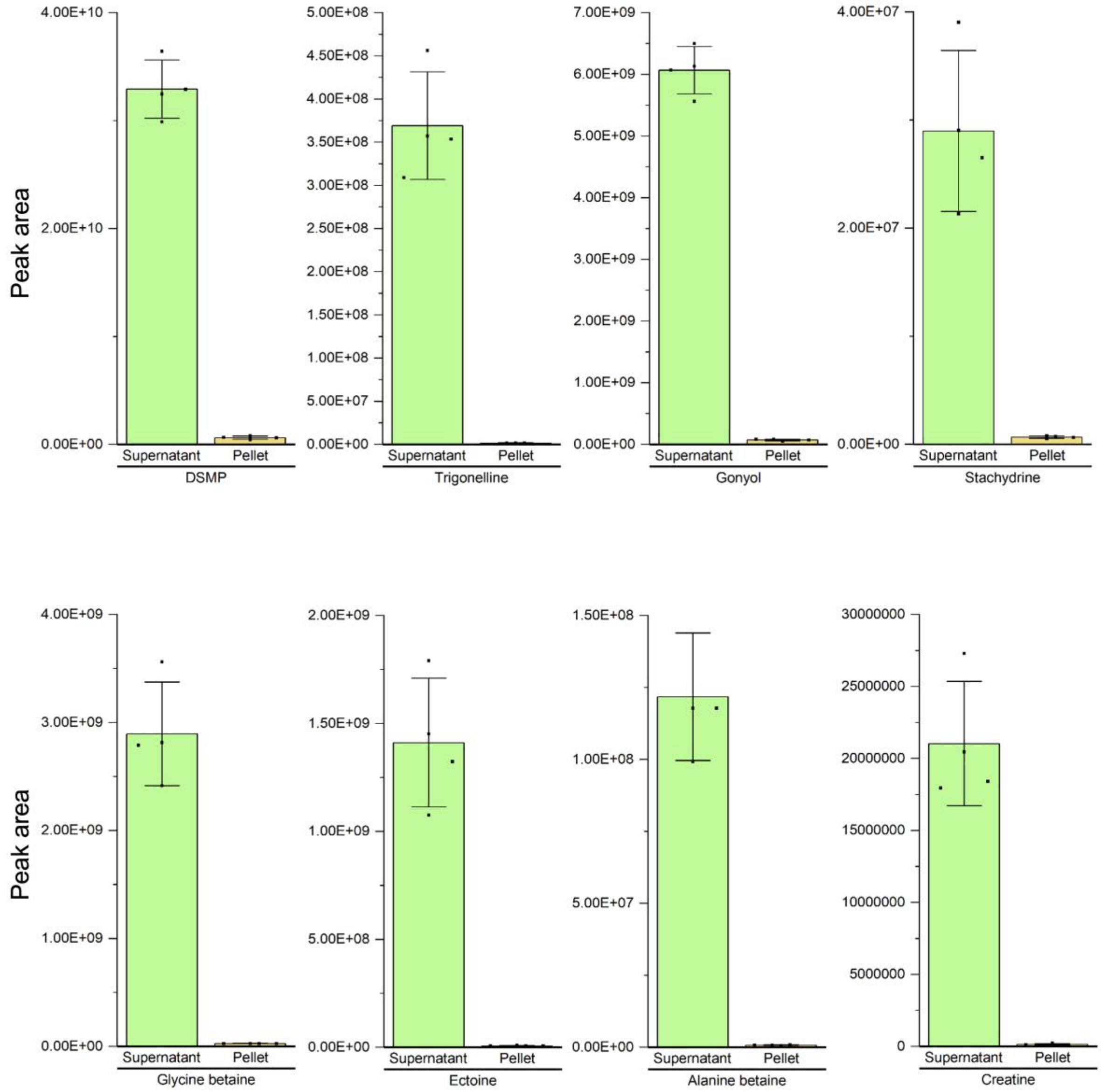

b

Compound	Total peak area	Proportion of supernatant peak area, %	Proportion of pellet peak area, %
DSMP	33542640386	98.14	1.86
Trigonelline	370550188	99.59	0.41
Gonyol	6137366439	98.84	1.16
Stachydrine	29649548	97.75	2.25
Glycine betaine	2921004434	99.09	0.91
Ectoine	1416838943	99.57	0.43
Alanine betaine	122508903	99.40	0.60
Creatine	21165876	99.36	0.64

Supplementary Figure 2: Efficiency of metabolite extraction from algal lysate. (**a**) Peak areas for the metabolites detected in supernatant (green) and pellet (yellow) of the algal lysate extracts; (**b**) Calculated proportions of the metabolites present in the algal lysate extracts supernatants and pellets. Biological replicates were n = 2, and technical replicates were n = 2. Data are presented as mean ± SD. Source data are provided as a Source Data file.

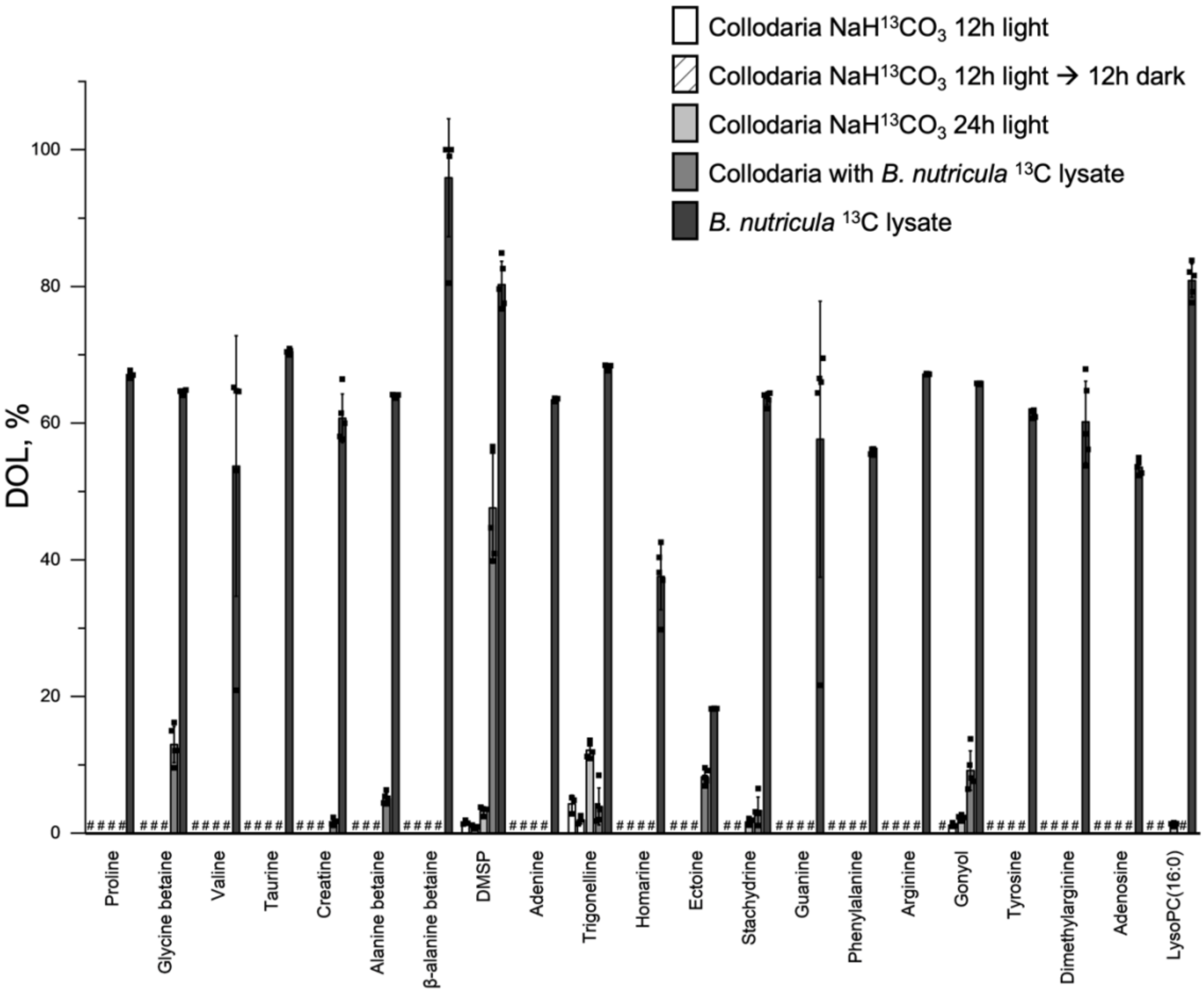

Supplementary Figure 3: Degree of labeling (DOL) of Collodaria holobiont metabolites after incubation with ^{13}C-substituted bicarbonate and ^{13}C-labeled algal lysate, and *Brandtodinium nutricula* metabolites in the lysate. # – no ^{13}C label incorporation was detected. Biological replicates were n = 3 for $NaH^{13}CO_3$ incubations under 12 h light and 12 h light/12 h dark, n = 5 for the 24 h light $NaH^{13}CO_3$ incubation and for incubation with ^{13}C-labeled algal lysate, and ^{13}C labeled algal lysate; the same number of replicates was used for the corresponding non-labeled controls. Data are presented as mean ± SD. Source data are provided as a Source Data file.

Supplementary Table 1: List of metabolites analyzed in the study. LC-HRMS analysis of metabolites was semi-targeted based on the metabolites detected previously [1], GC-HRMS analysis was untargeted; identity of ^{13}C labeled metabolites was verified with analytical standards; "+" indicates the presence of the metabolite in the previous study [1], "-" indicates absence of the compound, "n/a" – not accessed.

Compound class	Compound	Detected in previous metabolomic study [1]		Analytical method
		Collodaria holobiont	Free-living *B. nutricula*	
Amino acids and derivatives	Proline	+	+	LC-HRMS
	N,*N*-Dimethylarginine	+	+	
	Arginine	+	+	
	Valine	+	+	
	Phenylalanine	+	+	
	Tyrosine	-*	-*	
	Ectoine	+	+	
	Creatine	+	+	
	Taurine	-	+	
Nucleobases	Adenine	+	+	
	Guanine	+	+	
Nucleosides	Adenosine	+	+	
Phosphocholines	LysoPC(16:0)	+	+	
Betaines	Glycine betaine	+	+	
	Trigonelline	+	+	
	Stachydrine	+	+	
	Alanine betaine	+	+	
	β-Alanine betaine	+	+	
	Homarine	+	+	
	DMSP	+	+	
	Gonyol	+	+	
Monosaccharides	Fructose	n/a	n/a	GC-HRMS
	Glucose	n/a	n/a	
	Ribose	n/a	n/a	
Sugar alcohols	Deoxyinositol**	n/a	n/a	
	Myo-inositol	n/a	n/a	
	Scyllo-inositol	n/a	n/a	
Sugar acid	Threonic acid	n/a	n/a	
Fatty acid estrer	Methyl palmitate	n/a	n/a	

*In that study metabolite identification was restricted to 200 most abundant metabolites, and tyrosine was not present within these 200 due to very low abundance. Present study focused only on ^{13}C enriched metabolites and tyrosine was among them.

**Deoxyinositol was only putatively identified based on the library hit due to high number on possible isomers.

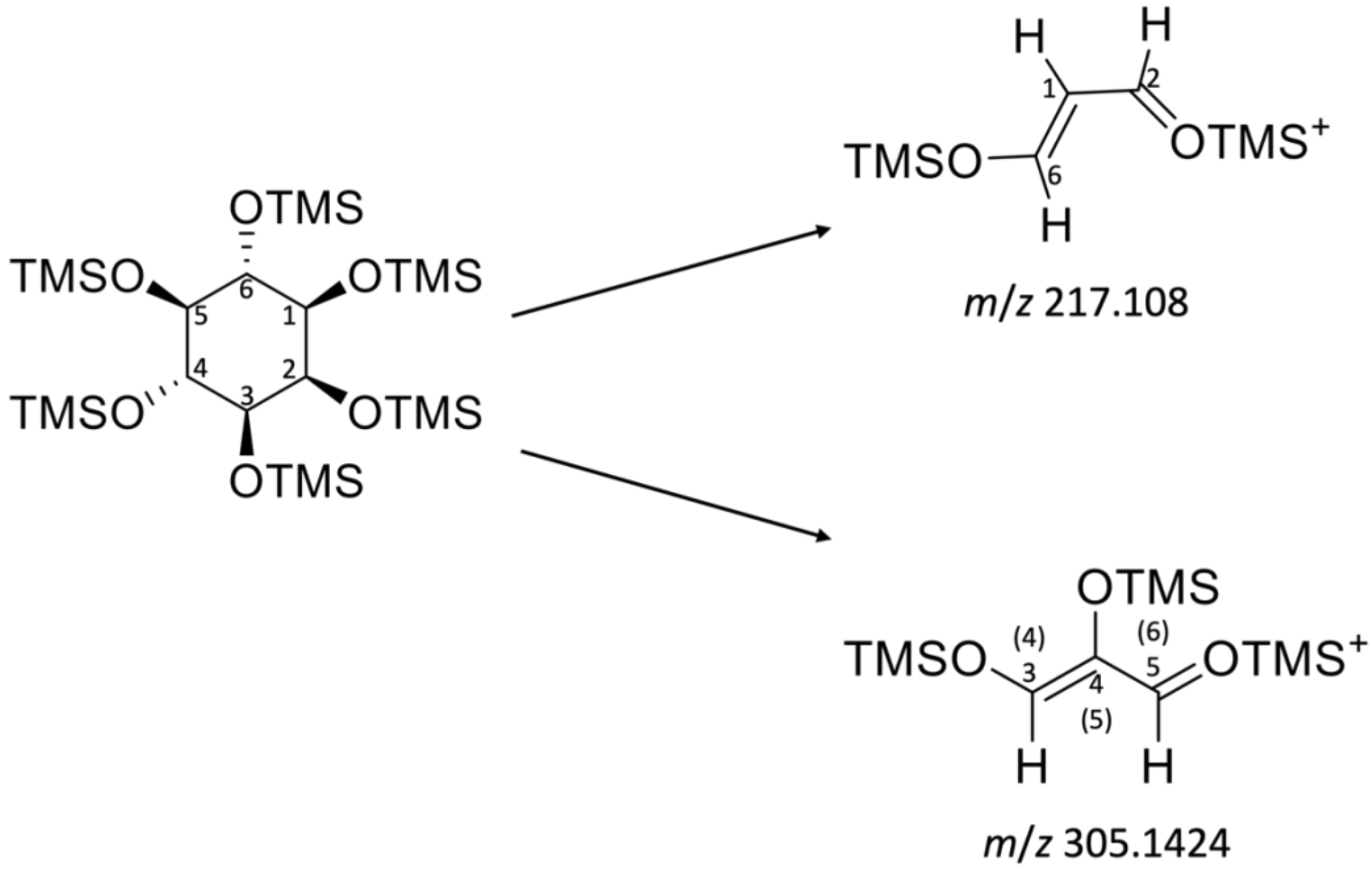

Supplementary Figure 4: Fragmentation of inositols; fragment 217.108 is also present for other carbohydrates [2,3]. TMS – trimethylsily.

References

1. Nikitashina, V. *et al.* Metabolic interdependence and rewiring in radiolaria-microalgae photosymbioses. *ISME J.* **19**, wraf047 (2025).
2. De Jongh, D. C. *et al.* Analysis of trimethylsilyl derivatives of carbohydrates by gas chromatography and mass spectrometry. *J. Am. Chem. Soc.* **91**, 1728–1740 (1969).
3. Sherman, W. R., Ackermann, K. E., Berger, R. A., Gish, B. G. & Zinbo, M. Analysis of inositol mono- and polyphosphates by gas chromatography/mass spectrometry and fast atom bombardment. *Biolomedical Environ. Mass Spectrom.* **13**, 333–341 (1986).
