# Supplementary material for: Metabolic partitioning between photosynthesis and osmotrophy in Collodaria photosymbiotic holobionts
Source: Nat Commun. 2026 Aug 19;17:8570. doi: 10.1038/s41467-026-76549-6 (PMC13490583; doi:10.1038/s41467-026-76549-6)
Supplement: Supplementary file 2 [file 41467_2026_76549_MOESM2_ESM.pdf]

<u>Description of Additional Supplementary Files</u>

Supplementary Code 1. The X13CMS script can be found with settings used for the study.
Supplementary Code 2. Gives a script based on a previously established R-based pipeline (Stettin et al. *Metabolites* **10**, 143 (2020)) optimized for the analysis of the data obtained in this study.
