# Supplementary material for: Metabolic partitioning between photosynthesis and osmotrophy in Collodaria photosymbiotic holobionts
Source: Nat Commun. 2026 Aug 19;17:8570. doi: 10.1038/s41467-026-76549-6 (PMC13490583; doi:10.1038/s41467-026-76549-6)
Supplement: Supplementary file 3 [file 41467_2026_76549_MOESM4_ESM.pdf]

nature portfolio

Corresponding author(s): Georg Pohnert

Last updated by author(s): Jun 8, 2026

Reporting Summary

Nature Portfolio wishes to improve the reproducibility of the work that we publish. This form provides structure for consistency and transparency in reporting. For further information on Nature Portfolio policies, see our Editorial Policies and the Editorial Policy Checklist.

Statistics

For all statistical analyses, confirm that the following items are present in the figure legend, table legend, main text, or Methods section.

n/a	Confirmed	
☐	☒	The exact sample size (*n*) for each experimental group/condition, given as a discrete number and unit of measurement
☐	☒	A statement on whether measurements were taken from distinct samples or whether the same sample was measured repeatedly
☐	☒	The statistical test(s) used AND whether they are one- or two-sided<br>*Only common tests should be described solely by name; describe more complex techniques in the Methods section.*
☒	☐	A description of all covariates tested
☒	☐	A description of any assumptions or corrections, such as tests of normality and adjustment for multiple comparisons
☐	☒	A full description of the statistical parameters including central tendency (e.g. means) or other basic estimates (e.g. regression coefficient) AND variation (e.g. standard deviation) or associated estimates of uncertainty (e.g. confidence intervals)
☐	☒	For null hypothesis testing, the test statistic (e.g. *F*, *t*, *r*) with confidence intervals, effect sizes, degrees of freedom and *P* value noted<br>*Give P values as exact values whenever suitable.*
☒	☐	For Bayesian analysis, information on the choice of priors and Markov chain Monte Carlo settings
☒	☐	For hierarchical and complex designs, identification of the appropriate level for tests and full reporting of outcomes
☒	☐	Estimates of effect sizes (e.g. Cohen's *d*, Pearson's *r*), indicating how they were calculated

Our web collection on statistics for biologists contains articles on many of the points above.

Software and code

Policy information about availability of computer code

Data collection	Thermo Finnigan XCalibur 4.4.16.14
Data analysis	Raw data obtained from LC-HRMS analysis was preprocessed using Compound Discoverer version 3.3 SP3 (Thermo Fisher Scientific, USA), raw GC-HRMS data was preprocessed using R-based pipline that includes the XCMS package, CAMERA package, and MetaMS package, processing was done using R-based pipeline including X13CMS package.

For manuscripts utilizing custom algorithms or software that are central to the research but not yet described in published literature, software must be made available to editors and reviewers. We strongly encourage code deposition in a community repository (e.g. GitHub). See the Nature Portfolio guidelines for submitting code & software for further information.

Data

Policy information about availability of data

All manuscripts must include a data availability statement. This statement should provide the following information, where applicable:

- Accession codes, unique identifiers, or web links for publicly available datasets
- A description of any restrictions on data availability
- For clinical datasets or third party data, please ensure that the statement adheres to our policy

The authors declare that all data supporting the findings of this study are available within the paper and its Supplementary files, or public repository. The metabolomics data have been deposited to MetaboLights 75 repository with the study identifier MTBLS12592.

Research involving human participants, their data, or biological material

Policy information about studies with human participants or human data. See also policy information about sex, gender (identity/presentation), and sexual orientation and race, ethnicity and racism.

Reporting on sex and gender	n/a
Reporting on race, ethnicity, or other socially relevant groupings	n/a
Population characteristics	n/a
Recruitment	n/a
Ethics oversight	n/a

Note that full information on the approval of the study protocol must also be provided in the manuscript.

Field-specific reporting

Please select the one below that is the best fit for your research. If you are not sure, read the appropriate sections before making your selection.

☐ Life sciences ☐ Behavioural & social sciences ☒ Ecological, evolutionary & environmental sciences

For a reference copy of the document with all sections, see nature.com/documents/nr-reporting-summary-flat.pdf

Ecological, evolutionary & environmental sciences study design

All studies must disclose on these points even when the disclosure is negative.

Study description	Photosymbiont-bearing Collodaria colonies (Radiolaria) were collected using plankton nets from a boat, samples were immediately processed at the adjunct marine station.<br>To investigate photosynthatic incorporation of inorganic carbon Collodaria were incubated with 13C substituted NaH13CO3, to investigate assimilation of dissolved organic carbon, colonies were incubated with 13C labeled algal lyzate. Three to five biological replicates were prepared for each experiment, 12C unlabeled controls were precessed in parallel. After incubation was completed, Collodaria were washed in filtered seawater, immediately frozen in liquid nitrogen, and stored below –70°C until further processing.
Research sample	Photosymbiont-bearing Collodaria colonies (Radiolaria) were investigated in this study. Photosymbiont-bearing Collodaria represent planktonic photosymbiosis, however metabolic interactions within this symbiotic system is poorly understood.<br>Collected organisms were incubated in filtered seawater to allow them to clean from attached particles. These samples represent population of planktonic Collodaria.
Sampling strategy	Photosymbiont-bearing Collodaria colonies were collected using 200 µm plankton nets from a boat, samples were immediately processed at the adjunct marine station.<br>Six independent surface tows were collected with a plankton net and combined. Around hundred of colonies were hand-picked every day.<br>Number of biological replicates for the experiments was three to five which is already sufficient to perform statistical testing.
Data collection	Samples were run on GC-MS and LC-MS instrument by V. Nikitashina
Timing and spatial scale	All biological replicates for one setup (including 12C controls and 13C labeling) originate from the same sampling batch. Sampling was performed in four day intervals. GC-MS and LC-MS data was acquired within one run for each instrument respectively.
Data exclusions	No data was excluded.
Reproducibility	pre-experiments (non-replicated) were successfully performed to evaluate if proper replication is given
Randomization	All samples were randomized and recorded with the help of an autosampler
Blinding	Distribution of organisms between 12C controls and 13C experiments was blinded, data analysis was performed with programs, where control and treatment groups should be defined.

Did the study involve field work? ☒ Yes ☐ No

Field work, collection and transport

Field conditions	Morning sampling in spring (March) with a water temperature around 16°C. No strong winds, no precipitation.
Location	Villefrance sur Mer marine station and the Rade de Villefranche sur Mer, (France, 43°41´10´´N, 7°18´50´´E)
Access & import/export	Sampling was collected using plankton nets trom a boat. To ensure proper access a local skipper trained in scientific sampling was always on board.<br>ABS certificate (Reference number: TREL2302365S/655) and its international equivalent (ABS-CH Unique Identifier (UID): ABSCH-IRCC-FR-263703-1) were obtained from Ministère de la Transition écologique et de la Cohésion des territoires / Direction générale de l'aménagement, du logement et de la nature / Direction de l'eau et de la biodiversité / Bureau de l'encadrement des impacts sur la biodiversité on March 29, 2023.
Disturbance	plankton tows cover only a very small subset of the marine population and have no effect on the habitat

Reporting for specific materials, systems and methods

We require information from authors about some types of materials, experimental systems and methods used in many studies. Here, indicate whether each material, system or method listed is relevant to your study. If you are not sure if a list item applies to your research, read the appropriate section before selecting a response.

Materials & experimental systems

n/a	Involved in the study	
☒	☐	Antibodies
☐	☒	Eukaryotic cell lines
☒	☐	Palaeontology and archaeology
☐	☒	Animals and other organisms
☒	☐	Clinical data
☒	☐	Dual use research of concern
☒	☐	Plants

Methods

n/a	Involved in the study	
☒	☐	ChIP-seq
☒	☐	Flow cytometry
☒	☐	MRI-based neuroimaging

Eukaryotic cell lines

Policy information about cell lines and Sex and Gender in Research

Cell line source(s)	A strain of Brandtodinium nutricula RCC 3387 was obtained from the Roscoff Culture Collection (Roscoff France)
Authentication	Authentication has been perfromed by the curator of the culture collection
Mycoplasma contamination	n/a
Commonly misidentified lines (See ICLAC register)	n/a

Animals and other research organisms

Policy information about studies involving animals; ARRIVE guidelines recommended for reporting animal research, and Sex and Gender in Research

Laboratory animals	n/a
Wild animals	n/a
Reporting on sex	n/a
Field-collected samples	Collodaria colonies were sampled with a plankton net, incubated in a filtered seawater at ambient to seawater conditions. After incubation with either NaH12/13CO3 and 12/13C algal lysate the microorganisms were snap frozen in a liquid nitrogen, stored below –70°C until further processing.
Ethics oversight	Since Collodaria are microorganisms, no ethical approval is required.

Note that full information on the approval of the study protocol must also be provided in the manuscript.

Plants

Seed stocks	n/a
Novel plant genotypes	n/a
Authentication	n/a
