# Supplementary material for: Metabolic partitioning between photosynthesis and osmotrophy in Collodaria photosymbiotic holobionts
Source: Nat Commun. 2026 Aug 19;17:8570. doi: 10.1038/s41467-026-76549-6 (PMC13490583; doi:10.1038/s41467-026-76549-6)
Supplement: Supplementary file 4 [file 41467_2026_76549_MOESM5_ESM.pdf]

Peer Review File

Metabolic Partitioning between Photosynthesis and Osmotrophy in Collodaria Photosymbiotic Holobionts

Corresponding Author: Professor Georg Pohnert

This file contains all reviewer reports in order by version, followed by all author rebuttals in order by version.

Attachments originally included by the reviewers as part of their assessment can be found at the end of this file.

Version 0:

Reviewer comments:

Reviewer #1

(Remarks to the Author)
“Partitioning of carbon metabolism between photosynthesis and osmotrophy in Collodaria photosymbiotic holobionts” by Nikitashina and Pohnert aims to characterize the metabolic nature of photosymbionts using stable isotopes. Symbioses in microeukaryotes are diverse in nature, with many lineages serving as hosts and symbionts. Little is understood regarding the exact roles of hosts and symbionts, how the relationships are established and maintained, and how they contribute to carbon and nutrient cycling as compared to autotrophic or heterotrophic specialists. Demonstrating how little we understand about these symbioses in marine microeukaryotes, the endosymbiont cyanobacteria UCYN-A has been reclassified as a new type of haptophyte organelle in a groundbreaking recent study (Coale et al. 2024).

In this study, the authors were interested in examining photosymbionts that provide products of photosynthesis to a heterotrophic host, Collodaria, which is a member of the radiolarians. There is uncertainty in the literature in regards to how much fixed carbon is provided to the host from the photosymbionts, and what other secondary metabolites may be shared between them. To help address this, a stable isotope method is used to track carbon fixation products and metabolites. Experiments were conducted in the light, dark, and with the presence of organics to examine osmotrophic uptake. A noteworthy finding was that photosynthetically derived carbon represented 1/3 of the saccharide (carbohydrate/sugar) pool in the holobiont (host + symbiont), with no or very low amounts of sugar taken up via osmotrophy. Osmotrophic uptake of DMSP was significant, with 50% of the pool taken up by the holobiont. This represents an important, moderate advancement in our understanding of the radiolarian-photosymbiont relationship, building on the studies cited in the introduction and discussion.

The biggest weakness is that cell fractions were not analyzed separately, so it is unclear how much of the C13-photosynthetic products were trafficked into host cells vs. used directly by symbionts. On lines 207-215 the authors state that metabolites were analyzed in each partner, but based on the methods, I don’t see how this was done or how conclusions about this can be made. The introduction and discussion need to be more carefully worded to clarify this important limitation.

Can the authors comments on why 13-C carbonate was used as the tracer instead of bicarbonate, since bicarbonate is the relevant carbon species in the marine ecosystem at a pH of 8. Was the pH monitored to ensure large deviations did not occur as a function of tracer addition?

Line 243 – I am not sure you can comment on shuttling of products since they were only measured in the holobiont, not in individual partners.

Line 296 – Again comments are made about carbon products being trafficked, which is not possible based on this analysis.

(Remarks on code availability)

Reviewer #2

(Remarks to the Author)
The authors of the manuscript "Partitioning of Carbon Metabolism Between Photosynthesis and Osmotrophy in Collodaria Photosymbiotic Holobionts" use isotope labelling and mass spectrometry to perform a metabolomic analysis on the Collodaria holobiont. The authors found that, in the presence of light, labeled glucose and fructose are produced, which are then catabolized in the dark. Longer incubations in the light led to increased labeling of photosynthate as well as labeling of the catabolism products. Meanwhile, labeled osmolytes such as DMSP were detected predominantly in the labeled algal lysate treatment.
The work convincingly demonstrates that photosymbionts are photosynthetically active and contributing sugars to the Collodaria holobiont. This concrete evidence is a welcome contribution to the study of the Radiolaria photosymbiosis; however, the manuscript is quite difficult to follow as the aims and rationale for the experiments were not explained in detail, the methods are not explained to a broad audience, and the results were not placed in the context of radiolarian biology or previous research. In this context, we feel that the document requires revision for the following reasons:

General Comments:
There seems to be a conceptual disconnect between the authors' findings and their claims, although neither is clearly presented. The manuscript also lacks a clear biological rationale for the experiments. This is evident in the following ways:
• Collodaria (and other Radiolaria) are known heterotrophs, i.e., they phagocytose prey. This was only mentioned once, indirectly, in the discussion. Thus, if a collodarian were to acquire sugars through any other route than symbiont photoautotrophy, it would be heterotrophy, not osmotrophy. We suggest that the authors explain their choice of comparison here (osmotrophy vs. symbiont photoautotrophy) for the acquisition of carbon in the holobiont and place it in the context of Collodaria biology; that is, explain why a heterotrophy treatment was not employed.
• The osmotrophy treatment also requires some explanation. Why are B. nutricula lysates the chosen medium for assessing holobiont osmotrophy? A collodarian would never exist within a medium of its symbiont's lysates alone. Please describe in the text why algal lysates are an incomplete proxy for osmotrophy in Collodaria.
Perhaps a clearer explanation of the aims and rationale for the algal lysate experiment (as well as the other experiments) would address these issues. For example, if the aim of the labeled algal lysate experiment were to test whether Collodaria are capable of taking up DMSP from the environment, then the experiment was appropriate, and the conclusions are well supported. If, on the other hand, the aim was to determine whether Collodaria obtain carbon primarily by photoautotrophy or by heterotrophy, the experiment is not appropriate. It was also unclear whether and how the algal culture labeling was used as a control for interpreting the Collodaria data.
The work presented here confirms that collodarian symbionts are photosynthetically active, not that the collodarian host benefits from symbiont photosynthates. This is an important distinction because the former could be true of any alga stuck in the cytoplasm of any host, while the latter confirms the true nature of a photosymbiotic relationship. Please rephrase throughout the document to clarify this point.
Please define all acronyms the first time they are used. A significant amount of jargon is used throughout the article; please clarify methods, results, and interpretations to appeal to a broader audience. It is currently written for those with an expertise in isotope labeling and/or metabolomics exclusively.

Specific Comments:
Line 20 – "that allows to investigate" should be "that allows us to investigate" or "that enables investigation into"
Lines 26-27 – "suggesting no significant contribution of osmotrophy to the holobiont's carbon pool"; surely we do not expect significant carbon acquisition by osmotrophy in the open ocean?
Line 42 – "even if" should be "even though"
Line 51 – Should be "the proportion of the photosymbionts' contribution"
Line 62-66 – It's not obvious from this sentence that Collodaria is a subgroup of Polycystinea – please clarify this.
Line 77 – What is the reference for studies suggesting photosynthetic carbon is less than 1%?
Line 82 – Should be "but the more polar"
Line 88 – Clarify by stating "these symbiont pathways" rather than "these pathways"
Line 90 – How about "limited to monitoring isotope shuttling"
Line 93 – Remove the comma after "both"
Line 95 – Should be "allows us to characterize" or "that enables characterization of"
Line 96 – Should be "we identify the involved"
Line 97 again – "allows us to compare" or "allows/enables comparison of"
Line 100 – Remove "among others"
Line 109 – Should be "in the light to determine which metabolites are derived from"
Line 110 – Remove "allowed"
Line 111 – Change "allowed for monitoring of" to "to monitor"
Lines 111-112 – How does prolonged incubation in the light indicate substrate transfer in the holobiont? Substrate transfer implies transfer from symbiont to host, does it not? The aims and rationale of each labeling strategy should be explained in more detail.
Line 113 – Remove "allowed"
Line 122 – Change to "and/or"
Line 125 – What does degree of labeling measure, specifically?
Line 126 – Please spell out GC-HRMS and CI on first mention
Line 128 – Please spell out EI on first mention
Lines 121-128 – It would be helpful to briefly describe the mass spec procedure, without jargon, so that the reader can visualize it without jumping to the methods first.
Lines 121-123 – Please report the results in more detail, i.e., something like "we detected more mass signatures in the

DMSP spectrum for the osmotrophic treatment than the control or the phototrophic treatment". Also explain which treatment is shown, why only these two metabolites are shown, and what the results for the other treatments were.
Line 138 Fig. 2 legend – Please clarify which experimental treatment these spectra came from. Presumably the "osmotrophic assimilation" comes from the labeled algal lysate treatment, but the "photosynthetic assimilation" could come from any of the 3 labeled carbonate experiments. Which control is the control? Unlabeled cell lysates, or unlabeled culture medium? Also, presumably this is GC-HRMS data (not LC)? Which replicate samples were these spectra from, i.e., presumably you selected one nice spectrum to display, was there much variability among samples?
Line 144 – Add "the" before "holobiont's"
Line 147 – Remove comma after "by"
Line 149 – Add "the" before "holobiont's"
Line 153 Fig. 3 – How were these metabolites selected to display? Was there a threshold for degree of labeling that eliminated all other metabolites? Or was there a set of metabolites that you decided to target a priori? Was the DOL 0 for the controls as well? This could be stated in the legend.
Lines 148-151 – Is this statement intended to be agnostic about which partner is metabolizing the labeled C? Perhaps make this more explicit, otherwise it seems to be suggesting that the host is metabolizing the 13C compounds, which has not yet been demonstrated.
Line 171 – Change "along" to "alone"
Line 171-174 – Why would the DOL decrease for the hexoses? Is the holobiont preferentially metabolizing labeled hexoses? Or is the holobiont somehow fixing new unlabeled carbon into hexoses in the dark? Or am I misunderstanding DOL?
Line 181 – It is not necessary to say "statistically significantly" because "statistically" is implied. It would be better to remove "statistically" in this context throughout the ms.
Line 186 – How was it determined that the carbonates are photosynthetically derived? How does one determine the metabolic origin of a carbonate?
Line 189 – It seems that there is no way to be sure whether the labeled DMSP is incorporated by pinocytosis, phagocytosis, or true osmotrophy (i.e., importing solutes with transporters). Please include a sentence to explain why osmotrophy is more likely than heterotrophic modes of import, since the title of this section states that it is osmotrophy.
Line 209 – Change "a half" to just "half"
Lines 212-215 – How long were the incubations for the free-living alga in ref 32? Also, could there not be an explanation besides slower metabolism in the holobiont? Perhaps the host is taking most of the fixed carbon and metabolizing it along different pathways than what the alga would use it for? Particularly consider that many amino acids are likely to be synthesized by the alga and not the host.
Line 217 – Rather than "obtains" perhaps say "incorporates 13C into"
Line 220 – Change to "glucose, fructose, and derived metabolites"
Line 221 – Change "whereas" to "conversely" or else link this sentence to the previous with a comma after "metabolites".
Lines 237-239 – How do we know that Collodaria cannot survive without photosynthetically active symbionts if we cannot cultivate them?
Line 240 – Is "translocation" really the best word? It suggests carbohydrate transporters, whereas your data concerns metabolites not transporters.
Line 241 – Please reword. "Could be achieved" sounds like it might happen in the future, when you have already achieved it in the past. Also, what is a "compound-resolved analysis"?
Line 243 – I think "metabolism" would be more accurate than "shuttling" in this context?
Better still, remove the first paragraph. It communicates nothing of substance and has several slight inaccuracies.
Line 257 – Remove the "a" after "accumulation"
Lines 257-261 – It would be helpful to describe how the accumulation of photosynthetically derived glucose in photosymbiotic hosts was determined.
Line 263 – Please include a description of what these molecules are, what they do, and why they are important (this is not common knowledge).
Line 271 – Please describe why these compounds are thought to indicate that they originate from algal photosynthesis
Line 275 – Change to "the jellyfish photosymbiosis"
Lines 316-321 – Heterotrophic feeding is not the same as osmotrophy, especially not in a lineage that is known to consume prey as large as copepods. While your hypothesis is sound given what we know about heterotrophy, generally, it does not follow that metabolites acquired via osmotrophy would be the same metabolites acquired via heterotrophic feeding.
Line 337 – Remove parentheses around "filled to the top of the rim" and remove following comma
Line 387 – I believe it should be "Villefranche-sur-Mer"
Line 405 – Better would be "A minimum of" or "At least"
Line 409 – Fix typo to "concentration"
Line 425 – Change "till" to "until"
Line 425 – Does this mean 7 to 9 colonies were prepared in each setup in each replicate? Or divided among setups so that each setup only had 2-3 colonies?
Line 426 – Does this mean that each setup had the same number of colonies across replicates, or each replicate had the same number of colonies across setups?
Line 428 – Fix typo C-labeled
Line 429 – "culture" should be "cultures"
Line 433 – Change "till" to "until"
Line 441 – Change "till" to "until"
Line 441 – Please clarify whether the 11 colonies were divided among the two treatments for each replicate, or whether each treatment had 11 colonies in all replicates.
Line 455 – Remove "a" before "room temperature"

Line 455 – Change “spitted” to “split”
Line 523-524 – Please check this sentence. The comma is in the wrong place and the sentence is incomplete.
Line 566 – What does “downstream processing” mean, please provide specifics

(Remarks on code availability)
We cannot fully evaluate the code as it is beyond our areas of expertise.

Reviewer #3

(Remarks to the Author)
See attached document

(Remarks on code availability)

Reviewer #4

(Remarks to the Author)
I co-reviewed this manuscript with one of the reviewers who provided the listed reports. This is part of the Nature Communications initiative to facilitate training in peer review and to provide appropriate recognition for Early Career Researchers who co-review manuscripts.

(Remarks on code availability)

Reviewer #5

(Remarks to the Author)
General comments:
The manuscript entitled “Partitioning of Carbon Metabolism Between Photosynthesis and Osmotrophy in Collodaria Photosymbiotic Holobionts” by Nikitashina and Pohnert reported the metabolic dependencies between Collodaria hosts and dinoflagellate symbionts using isotopic labeling techniques. Radiolarians, particularly the colonial group Collodaria, are ubiquitous and abundant marine protistan plankton that significantly contribute to the global carbon cycle. It is therefore crucial to develop a comprehensive understanding of ecological strategies, such as photosymbiosis, in order to gain a more profound insight into the subject. Their results demonstrated that glucose and fructose are the major primary metabolites synthesized by the symbiont through photosynthesis. In contrast, osmolytes such as DMSP and betains are of environmental origin, rather than being derived from the photosynthetic pathway. I would like to note that I am not an expert in metabolomics or isotope labeling approaches; therefore, I cannot comment extensively on the technical aspects of the analysis. From the viewpoint of plankton photosymbiosis in general, I would like to applaud their achievement, demonstrating what is produced and transferred among the symbiotic system. The results are sound, although their major finding—namely, sugar synthesis by the symbionts and its subsequent transfer to the host—is something that can be predicted in general. The DMSP accumulation path is interesting; however, I think further discussion is required for this part. Please find the comments below.

・Comments on DMSP
High DMSP concentration in holobiont is generally attributed to their symbionts, but in the case of radiolarians, it has been shown that symbiont contribution alone cannot explain the observed high concentration. This kind of background needs to be explained in the introduction; otherwise, why the authors are focusing on DMSP would not be easily understood by the audience.
This study clearly showed that DMSP of holobionts is derived from both symbiont photosynthesis and from the environment, but the contribution is far higher for the latter, although another possible pathway, the ability of DMSP synthesis by the host itself, cannot be discussed from this experiment. The higher contribution of DMSP incorporation from the environment indicates that Collodaria photosymbiosis is a sink of DMSP. I think this view is important since the size of the environmental pool of DMSP that will be decomposed to generate DMS is reduced. If my understanding is correct, this would also be a strong point of this study.
Whole through the manuscript, quantitative consideration is scarce. I understand that the experiments employed in this study does not allow quantification of the flux of the metabolites, but at least the information on the content of metabolite in the lysate needs to be provided to interpret the results. If the DMSP concentration in the incubation medium was too high compared to the natural environment, the osmotrophic incorporation of DMSP demonstrated in this study may not be a realistic pathway.

・Incubation with labeled metabolites
In this study, the authors used lysates of labeled symbiont (free-living B. nutricula culture) in experiment iv. It is unclear to me whether this mimics the condition of the symbiont digestion process, or the uptake of dissolved organic matter (DOM) from the surrounding environment. I assume that the primary purpose is the latter. If so, why use the specific symbiont rather than common microalgae such as Isochryisis or Thalassiosira? Or does this experiment serve both intentions? Please clarify.

・Class of compounds
Including me, for those who are not familiar with metabolite names, it is very helpful if we can find compound class information elsewhere in the manuscript. The simplest way is to add that information in Figure 3, or add a table of the metabolite discussed in this study. Please consider this addition.

・Not labeled or not detected
In Figure 3, ND (not detected) is used for DOL level. Does it mean 0% DOL (the unlabeled compound was detected) or that even the unlabeled compound was not detected? Presence or absence information is also important, so please clarify.

・Interpretation of the difference between the photosynthesis-derived metabolite and the algal lysate
Comparing the difference in DOL between the metabolite of holobiont incubated with 13C-bicarbonate (products of in hospite B. nutricula) and labeled algal lysate (products of free-living B. nutricula), as shown in Appendix S2, many metabolites are labeled only for the free-living algal lysate, although it was also originally from photosynthetically assimilated carbon. In my understanding, it indicates that (1) the metabolism is altered when they are in symbiotic states, (2) the metabolite was quickly utilized by the host, (3) the metabolite synthesis was too slow to detect the label, or (4) the metabolite pool is so large that the relative proportion of newly synthesized labeled metabolite was too low. In the discussion, it seems the possibility (3) alone is considered (L212-215). Please discuss other possibilities.
I checked their previous publication, Nikitashina et al. (2025, ISME J), and found that Taurine was not detected in the holobiont, but was detected in free-living. This may be the case of (1) or (2) (presence or absence information is also needed). In addition, in Nikitashina et al. (2025), Tyrosine was absent in the free-living symbiont, which is inconsistent with the present result (Appendix S2). Please explain/discuss it.

・Phagotrophy
Since the target species are heterotrophic, knowledge of their natural food/prey needs to be explained. Do they eat living plankton or detritus, or just perform osmotrophy?

・Threonic acid
Compounds whose DOL increased after 12h-12h light-dark condition compared to 12h light condition were interpreted to reflect rapid hexose metabolism into those compounds (ribose, deoxyinositol, gonyol). In terms of threonic acid, it also increased after light-dark cycle, but not discussed in the discussion. Besides, the result of threonic acid for 12h light incubation in Figure 3 is missing. Does it mean “ND” or not detected at all? Please clarify, and include the discussion on this compound in the main text.

Other points
Throughout the text: 13C-carbonate should be 13C-bicarbonate. Carbonate and bicarbonate are different.

L209: “DOL for these compounds” --> needs to be more specific.

L277: Lipid involvement in pelagic photosymbiosis was demonstrated by LeKieffre et al. (2018). This should be cited here.

L321: Need references. Many previous studies showed this idea on N and P requirement in the photosymbiotic system.

L399-400: Please provide the temperature of the ambient seawater.

L434: Please explain the reason for the final concentration of algal lysate (3.5 x 104 cells mL-1). Is it expecting the natural environment or mimicking the phagotrophic condition?

L436: Why was this experiment terminated after 22h, not 24h? In the three experiments using NaH13CO3, when the specimens were transferred to FSW, it was defined as the end of the experiment, so in the forth experiment case, the experimental duration was 22h. Figure 4d needs to be corrected accordingly.

Figure 3: Thrigonelline --> Trigonelline

Figure 4: 13CO2 is not correct. It should be NaH13CO3. This kind of schematic illustration is fine, but this one is not so much informative (the information in this figure can easily be understood from the text). Alternatively, I would suggest to include a figure of experimental design (the experimental condition and flow of the analysis), in addition to this result illustration.

(Remarks on code availability)
Readme file is not available.
I could not run the code since the data is not yet accessible from the data repository. I suppose when the paper is published, the data will be open in the database (MetaboLights ID: MTBLS12592 is provided, but not accessible at this moment). So I cannot evaluate the correctness of the code.

Reviewer #6

(Remarks to the Author)
Overall, this is a well-designed and carefully executed study that provides valuable metabolic insight into Collodaria–algal symbiosis. The combination of stable isotope labeling and metabolomics is particularly effective for resolving carbon assimilation and transfer pathways within the holobiont.
However, while the findings are clearly presented and advance our understanding of Collodaria specifically, the broader patterns reported here—namely, the transfer of photosynthetically derived carbohydrates from algal symbionts to the host and the partitioning of phototrophic versus heterotrophic contributions to host metabolite pools—are well documented across many marine photosymbioses, including corals, jellyfish, foraminifera, and bivalves. In this sense, the results support and extend already established concepts rather than revealing fundamentally novel mechanisms of symbiont–host interaction.
That said, the study still fills an important gap by applying these approaches to Collodaria, a group for which such metabolic data are limited, and it provides a valuable foundation for future work on the ecological role and biogeochemical significance of radiolarian photosymbioses.

The manuscript would benefit from additional attention to the strength and framing of several conclusions, particularly where the results reiterate patterns already well established across other marine photosymbioses. In addition, there are numerous grammatical and phrasing issues throughout the text that hinder clarity. Addressing these points will substantially improve the precision, readability, and overall impact of the manuscript.

Here are my more specific comments:

Line 44-45 - This sentence is a bit odd and doesn't really fit into the narrative of the paragraph

Line 91 - Do you mean to say 'about the metabolites involved' rather than the involved metabolites?

Line 94 - Perhaps ive never heard this term before and stand corrected if it is a common term I'm unfamiliar with, but what do you mean by involved metabolites? Do you mean metabolites involved in a particular process of interest? If this is so, I think this needs to be a bit clearer and explained in each instance.

Line 96 - You identify involved metabolites, do you mean bioactive metabolites?

Line 97 - Perhaps a typo here. I think you mean to say 'this allows comparison….' Or this method allows us to compare…..'

Line 102 - Why is DMSP a 'specialised metabolite' ? DMSP is very common among many bacteria and photosynthetic single cell organisms.

Line 144 - Here you mention 'holobiont' do you mean that here you're measuring the metabolites deriving from all organisms e.g. bacteria, photosymbiotic algae and collodarians? If that is the case, perhaps just define once that you're refering to all components.

Line 145 - Here you can just say 'after 12h of light incubation'

Line 148 - Typo here, I think you mean to say 'to a minor extent'

Line 149 - I would continue to refer it as the collodarian holobiont or collodaria holobiont, not just holobiont.

Line 163 - Remove 'a' from a significantly lower, just say 'resulted in significantly lower'

Line 169 - Should read 'dark incubations' as I imagine there was replication and not just one.

Line 171 - Typo: Should be 'alone' not along

Line 180, 165 etc. - Remove statistically from statistically significantly as this doesn't make sense. You can say statistically significant, but not in the way you've written this sentence. Just say 'significantly' throughout. Significant implies that it was statistically significant.

Line 181 - What two previous setups? Best to just briefly explain what you are referring to here.
Also, please change throughout 'statistically significantly' as this doesn't make sense. In this case here, you can just say: The DOL of Ribose was significantly higher in comparison….

Line 185 - Again explain these setups briefly as its not clear.

This is not clear what you mean here. The photosynthetically derived carbonate showed an increase where? Compared to the dark treatment? This is not at all clear to me what you're comparing to here.

Discussion

Line 241 –'this could be achieved' this should be in past tense and should read: 'This was achieved'

Line 243 – same here. Should read 'This approach allowed us'

Line 244 - I really think this is unlikely that DMSP originates from osmotrophy. The abundance of DMSP produced by the algal symbiont would likely far outweigh anything that is up taken from the surrounding environment. I understand you have detected labelled DMSP originating from the lysate, but I'm sure you must have detected it at much higher concentrations from the symbiotic collodaria also?
Ok after reviewing figure 3 further, I can see that the labelled portion of DMSP is much higher from the lysate labelled fractions. This is an interesting finding indeed. My question is though, how dense were these labelled lysate cultures? For example do you think the amount of lysed cells was more than what would be actually present within the actual collodaria cells? Is there any way you could show this? It would be very useful if this information was provided.

Line 255 - Is this really new information here? Isn't it already quite well established that photosymbionts provide hosts with glucose and in some cases fructose. Is this the first time it has been shown in Collodaria? I would say that this is highly likely.

Line 265 - need to restructure the sentence here and remove the full stop between sea anemones and the word 'and' . Sentences rarely start with the word 'and'

Line 268 - I think this is a somewhat weak conclusion, as this is already common knowledge in many other symbiotic relationships. If anything, you would need to convince the reader why this might be an interesting finding within Collodaria. Why would we presume otherwise that this would or would not be the case?

Line 428 typo – should read 13C labeled

Line 429 typo- should read 'cultures'

Line 437 - This sentence doesn't read well. I think you mean to say something like 'To ensure residual lysate was removed…..'

Line 449 - You might want to rephrase this to something like; 'and 1 µL from each sample was combined to create the pooled quality-control (QC) sample'.

Line 455 - Should read 'incubated at room temperature' spitted? Do you mean split?

Figure 3.
Black and white figures are unnecessary these days as all journals are printed in colour or found online in colour. I would recommend making this figure more visually stimulating by adding some colour to it.

(Remarks on code availability)

Version 1:

Reviewer comments:

Reviewer #1

(Remarks to the Author)
The authors have done an appropriate job clarifying their study organism and research question in the introduction, and the manuscript is more defined and clear. The results now support the revised, more modest claim, and remove mention of carbon products being trafficked between holobiont partners. However, as other reviewers also pointed out, it boils down to a study examining the metabolites shared by photosymbionts with their radiolarian host.

Line 387 highlights their main findings: "Although our approach did not allow us to distinguish between host- and symbiont-derived metabolites within the holobiont, the comparatively high proportion of labeled carbohydrates, and relatively small contribution of the photosymbionts to the holobiont
biomass, suggests that the detected carbohydrates were present in both the symbionts and the host. Therefore, based on our observations we hypothesize the following Collodaria–photosymbiont metabolic interactions: During photosynthesis, algae assimilate carbon and transfer it to the host as glucose and fructose, which are predominantly used for respiration to generate energy."

It is well established that photosymbionts will produce photosynthesis/energy products and provide them to their host, and it's also well understood that many protists engage in osmotrophy. Although interesting to detail this metabolic transfer, and valuable to the study of radiolarian ecology and physiology, the results will not necessarily be surprising to those outside the field. This work may therefore be more appropriate in a discipline-specific journal.

(Remarks on code availability)

Reviewer #2

(Remarks to the Author)
The authors of the manuscript “Partitioning of Carbon Metabolism Between Photosynthesis and Osmotrophy in Collodaria Photosymbiotic Holobionts” use isotope labelling and mass spectrometry to perform a metabolomic analysis on the Collodaria holobiont. The authors found that, in the presence of light, labeled glucose and fructose are produced, which are then catabolized in the dark. Longer incubations in the light led to increased labeling of photosynthate as well as labeling of the catabolism products. Meanwhile, labeled osmolytes such as DMSP were detected predominantly in the labeled algal lysate treatment. The work convincingly demonstrates that photosymbionts are photosynthetically active and contributing sugars to the Collodaria holobiont. This concrete evidence is a welcome contribution to the study of the Radiolaria photosymbiosis.
The revised version is substantially improved. Our major concerns were that the specific aims and rationale for each experiment were not sufficiently clear, the methods were not explained to a broad audience, and the results were not interpreted in the context of radiolarian biology or previous research. In this version, the rationale for each experiment in the list is clearer and more accurately expressed, and the additional information in the results section is helpful. The methods have less jargon and are easier to understand.
However, while there was improvement in placing the results in the context of radiolarian biology, such as adding the mention of phagotrophy to the introduction, there is still room for improvement in this area. Specifically, the authors have argued in their rebuttal that they demonstrated that there is no particle-associated carbon that could have been phagocytosed during the experiment. This may be true, but it is a separate question from our concern, which is that phagotrophy is certainly biologically relevant to Collodaria in the ocean while osmotrophy may or may not be. This should be emphasized more clearly throughout the “Contribution of osmotrophy to Collodaria metabolism” section, and in the discussion (~lines 311-312, 393-394) that the sources of these labeled compounds in this experiment might not reflect the important sources for Collodaria in the ocean, because phagotrophy could not be tested here.

Additional Comments:
Introduction
Line 51: Consider adding “For example,” before “In some sea slugs” because otherwise the preceding sentence sounds as if it is missing a citation.
Lines 51-54: All of the references here are to photosymbioses in animals. It would be more relevant to cite research on photosymbioses in protists. In particular, the sea slug is probably not the beset example because in sea slugs it is doubtful whether the plastids remain active or transfer sugars to the host.
Lines 63-67: Please reconsider these sentences because it is unclear and seems speculative and perhaps even incorrect. Whether or not radiolaria have mechanisms to take up dissolved organic matter was not directly tested in this study. The results suggest that certain organic compounds accumulate in the holobiont, but they could be accumulated by the algae, not the radiolaria, or the accumulation could be passive, perhaps by adherence to the extracellular matrix. Furthermore, the terms “organic carbon” and “nutrients” in this context are vague and potentially misleading. The results of this study suggest that photosymbionts fix carbon that is then respired, but the organic compounds taken up are not likely a significant energy source. Therefore the terms “vitamins” or “micronutrients” might be more relevant than “nutrients”, but again direct evidence is lacking. Finally, none of the works cited here support the suggestion that heterotrophic (and/or mixotrophic) protists engage in osmotrophy. These sentences distract from the more important point at the beginning of the paragraph, which is that radiolarians are known to ingest large prey and this is therefore likely a very important source of nutrients, at least equal to photoautotrophy, and remains an open question as to its nutritional contribution relative to the photosymbionts. Whether radiolarian cells engage in osmotrophy is currently speculative.
Line 71: The cited source here is not relevant for the stated information.
Lines 87-88: Please add a citation for “an earlier study”.
Lines 104-109: Please mention how DMSP is important to algal cell metabolism in addition to describing its importance to the sulfur cycle.
Line 112: Correct the strange typographical error “12 h”
Line 113: Remove comma after “both”
Lines 126-128: It seems a bit facetious to state that there was a deliberate focus on osmotrophy when the truth is that osmotrophy might or might not be biologically relevant for radiolaria. It is certainly less relevant than phagotrophy. Perhaps a more nuanced justification, acknowledging that phagotrophy is more important in life but less experimentally feasible, for example, would be more appropriate here.
Results
Line 135: The statement “we were able to elucidate the contribution of the respective partners” sounds somewhat inappropriate in tone because at this point the results have not yet been presented. How about “we aimed to elucidate”? It would make better grammatical sense with “we therefore” later in the sentence.
Lines 244-247: Please reword to be clearer about the lysate concentration. Does this sentence mean that the concentration was ten times lower than symbiont density and 100 times lower than an algal culture? Or does it mean 10-100x lower than various measurements for symbiont and algal culture density?

Line 262: Please add “in this experiment” to the end of this sentence to avoid giving the impression that osmotrophy is a significant process in Collodaria in general, because phagotrophy is likely far more important but was not directly studied.
Line 277: “synthetized” should be synthesized.

Discussion
Line 311: Please qualify this statement with "in the absence of phagotrophy" or "in this experiment, in the absence of phagotrophy"
Lines 333-334: Please remove the comma after "hypothesized" and change "but a prove was not documented" to "but this has not been demonstrated"
Line 369: Another strange typo "12 h".
Line 369: "the total DOL was analytically not accessible" please explain/clarify this statement.
Line 373: Change "in favor of the first mechanism speaks an increase in label incorporation" to "in favor of the first mechanism, there was an increase in label incorporation".
Lines 393-394: Please remind the reader that phagotrophy was not considered in this experiment.
Lines 396-401: Again, please be clear that these are not generalizable results to Collodaria in the ocean, but simply a demonstration of different carbon uptake mechanisms in this experiment, in the absence of phagotrophy.
Lines 412-415: This concluding sentence is a bit awkwardly worded and seems to be making a wider generalization than is warranted by the data. The papers cited previously did not quantify carbon allocation by heterotrophy relative to photoautotrophy, only that light was essential and prey items were interchangeable.

Methods
Line 497: Remove strange symbol typo in "March".

(Remarks on code availability)
The code used for isotope analysis is beyond our expertise to review.

Reviewer #4

(Remarks to the Author)
I co-reviewed this manuscript with one of the reviewers who provided the listed reports. This is part of the Nature Communications initiative to facilitate training in peer review and to provide appropriate recognition for Early Career Researchers who co-review manuscripts.

(Remarks on code availability)

Reviewer #5

(Remarks to the Author)
I thank the authors for their detailed responses and for considering all of my suggestions and comments. I confirmed that significant revisions were made to the manuscript according to my review, as well as the other reviewers'.
I have only a few additional minor comments.

-Host-symbiont signaling
In the manuscript, they mentioned the possible function of inositols as host-symbiont signaling, and this could be a general mechanism in photosymbiosis. In my understanding, general signaling molecules are e.g., lectins (C-type lectins). Although I understand that multiple substances may play a role in signaling, and inositols may be the ones, this part of the discussion (L331–338) sounds simplistic. It requires a more holistic review of host-symbiont signaling, what is known in the other photosymbiosic systems.

L110: "12 h" at the top should be deleted.

L345-347: Please also cite LeKieffre et al. (2018, Marine Biology), which discussed lipid transfer from symbionts to the host in planktonic foraminifera photosymbiosis. Actually, the reference I cited in the previous review report was LeKieffre et al. (2018, Marine Biology), not LeKieffre et al. (2018, Scientific reports). The latter also addressed the lipid transfer, but for benthic foraminifera.

L361: Threonic acid, in turn, is formed form from… ---> formed from

L369: "12 h" at the top should be deleted.

(Remarks on code availability)

Reviewer #6

(Remarks to the Author)
I have reviewed the revised manuscript and am pleased to see that the authors have carefully and thoroughly addressed all of my previous comments. The revisions have strengthened the manuscript, and this great study now presents a clear and well-supported contribution to the field. I only have a small number of remaining grammatical and phrasing suggestions, which are provided below. Presuming these very minor changes are addressed, I will not need to see the manuscript again

and can recommend it for publication in this journal.

Here are my very minor comments:
Line 17 – you can write just ‘ maintenance of photosymbiosis’
Line 31 – remove ‘the’ can be written as just ‘fluxes in plankton’
Line 35 – Again, this sounds a little odd here because of the inanimate passion phrasing. Here’s a suggestion to make the sentence flow better:’ Phototrophic microorganisms inhabiting the upper layers of the water column form the foundation of the marine food web and contribute significantly to the global carbon cycle via photosynthesis’
Line 47 – change to ‘suggests that photosymbionts provide organic compounds’
Line 78 maybe put ‘still’ or ‘currently’ not fully understood.
Line 86 – should be ‘products’ with an s.
Line 89 – ‘but other more polar metabiolites….’
Line 110 – there seems to be some error with the beginning of the sentence here, I think the 12 should have been deleted. I also suggest to start the sentence with: ‘Here we address the origin….’
Line 112 – Suggestion: ‘ This method makes it possible to investigate…..’
Line 275 – sentence structure issues. ‘So that analyzed’ I’m unsure what you mean here, please address.

Discussion
Line 308 – ‘both sugars were’ ? perhaps should be in past tense?
Line 313 – Particularly is a strange way to start a new paragraph. I suggest its replaced with ‘In particular’ or just ‘We observed…’
Line 331 – I know I keep asking ‘the’ to be removed, but in this case this sentence would benefit from it. ‘depending on the species of the colonizing symbiont’.
Line 333 – grammatical error - ‘but no proof was documented’
Line 351 – change to ‘significant decrease’
Line 361 – form has been typed twice here, please fix.
Line 369 – typo at beginning of paragraph, please check
Line 373 – speaks an increase? This doesn’t make sense, could you please rephrase here.
Line 394 – remove ‘the’ you can put just Collodaria
Line 396 – Change ‘this fact’ to ‘this finding’
Line 401 - This sentence is a bit odd, I suggest a rephrase to something like this: All metabolites in this category exhibit high polarity, which may be a prerequisite for uptake, and all contain either nitrogen or sulfur, suggesting that osmotrophic assimilation of these compounds may also contribute to nitrogen or sulfur acquisition.
Line 443 – ‘together with that’ this is not correct phrasing here, please change. Do you mean to say ‘collectively’ ?
Line 445 – Do you mean to say? Through (or via) accumulation of high….?
Line 448 – ‘marine microbial communities’

(Remarks on code availability)
I have not reviewed the code as it falls outside of my expertise.

Version 2:

Reviewer comments:

Reviewer #2

(Remarks to the Author)
The revised manuscript is improved. Our main concern was the framing of the study which initially failed to distinguish between osmotrophy and phagotrophy and now describes the aims more clearly and in a way that aligns better with the methodology and results. The authors have explained the rationale for the algal lysate experiments better in the discussion section, and the caveats about the fact that their experiment did not address phagotrophy are now sufficiently prominent. We have no further suggestions for improvement.

(Remarks on code availability)

Reviewer #4

(Remarks to the Author)
I co-reviewed this manuscript with one of the reviewers who provided the listed reports. This is part of the Nature Communications initiative to facilitate training in peer review and to provide appropriate recognition for Early Career Researchers who co-review manuscripts.

(Remarks on code availability)

Open Access This Peer Review File is licensed under a Creative Commons Attribution 4.0 International License, which permits use, sharing, adaptation, distribution and reproduction in any medium or format, as long as you give appropriate credit to the original author(s) and the source, provide a link to the Creative Commons license, and indicate if changes were made.
In cases where reviewers are anonymous, credit should be given to 'Anonymous Referee' and the source.
The images or other third party material in this Peer Review File are included in the article's Creative Commons license, unless indicated otherwise in a credit line to the material. If material is not included in the article's Creative Commons license and your intended use is not permitted by statutory regulation or exceeds the permitted use, you will need to obtain permission directly from the copyright holder.
To view a copy of this license, visit https://creativecommons.org/licenses/by/4.0/

REVIEWER COMMENTS

Reviewer #1 (Remarks to the Author):

“Partitioning of carbon metabolism between photosynthesis and osmotrophy in Collodaria photosymbiotic holobionts” by Nikitashina and Pohnert aims to characterize the metabolic nature of photosymbionts using stable isotopes. Symbioses in microeukaryotes are diverse in nature, with many lineages serving as hosts and symbionts. Little is understood regarding the exact roles of hosts and symbionts, how the relationships are established and maintained, and how they contribute to carbon and nutrient cycling as compared to autotrophic or heterotrophic specialists. Demonstrating how little we understand about these symbioses in marine microeukaryotes, the endosymbiont cyanobacteria UCYN-A has been reclassified as a new type of haptophyte organelle in a groundbreaking recent study (Coale et al. 2024).

In this study, the authors were interested in examining photosymbionts that provide products of photosynthesis to a heterotrophic host, Collodaria, which is a member of the radiolarians. There is uncertainty in the literature in regards to how much fixed carbon is provided to the host from the photosymbionts, and what other secondary metabolites may be shared between them. To help address this, a stable isotope method is used to track carbon fixation products and metabolites. Experiments were conducted in the light, dark, and with the presence of organics to examine osmotrophic uptake. A noteworthy finding was that photosynthetically derived carbon represented 1/3 of the saccharide (carbohydrate/sugar) pool in the holobiont (host + symbiont), with no or very low amounts of sugar taken up via osmotrophy. Osmotrophic uptake of DMSP was significant, with 50% of the pool taken up by the holobiont. This represents an important, moderate advancement in our understanding of the radiolarian-photosymbiont relationship, building on the studies cited in the introduction and discussion.

We thank the reviewer for the critical and helpful assessment of our manuscript and the constructive comments.

The biggest weakness is that cell fractions were not analyzed separately, so it is unclear how much of the C13-photosynthetic products were trafficked into host cells vs. used directly by symbionts. On lines 207-215 the authors state that metabolites were analyzed in each partner, but based on the methods, I don’t see how this was done or how conclusions about this can be made.

We rephrased the sentence to avoid this misunderstanding (Lines 313-316): “We addressed the contribution of photosynthetic activity of the symbionts and of osmotrophy of the host each partner to the pool of metabolites shared by the Collodaria holobiont and free-living form of the algae (Appendix S4), by analyzing their DOL in each incubation.”

The introduction and discussion need to be more carefully worded to clarify this important limitation. We made appropriate changes – highlighted in grey.

Can the authors comments on why 13-C carbonate was used as the tracer instead of bicarbonate, since bicarbonate is the relevant carbon species in the marine ecosystem at a pH of 8. Was the pH monitored to ensure large deviations did not occur as a function of tracer addition? – we thank the reviewer for pointing this out, there was a typo throughout the manuscript. We used sodium bicarbonate as a tracer, which is also normally added to the growth medium in non-labeled form and doesn't affect the pH. We corrected this issue throughout the manuscript.

Line 243 – I am not sure you can comment on shuttling of products since they were only measured in the holobiont, not in individual partners. – we removed the sentence to avoid this claim.

Line 296 – Again comments are made about carbon products being trafficked, which is not possible based on this analysis. We agree with this comment. However, the comparatively high proportion of labeled metabolites and relatively small contribution of the photosymbionts to the total biomass suggests that the detected carbohydrates were present in both partners. Taking this into account, we phrase a hypothesis, which, of course, still needs to be proven but at least provides directions for future studies, such as targeted analysis of labeled carbohydrates in the host using e.g. mass spectrometry imaging. We added this clarification to the text to avoid any misinterpretation highlighted in gray lines 487-489.

Reviewer #2 (Remarks to the Author):

The authors of the manuscript "Partitioning of Carbon Metabolism Between Photosynthesis and Osmotrophy in Collodaria Photosymbiotic Holobionts" use isotope labelling and mass spectrometry to perform a metabolomic analysis on the Collodaria holobiont. The authors found that, in the presence of light, labeled glucose and fructose are produced, which are then catabolized in the dark. Longer incubations in the light led to increased labeling of photosynthate as well as labeling of the catabolism products. Meanwhile, labeled osmolytes such as DMSP were detected predominantly in the labeled algal lysate treatment.

The work convincingly demonstrates that photosymbionts are photosynthetically active and contributing sugars to the Collodaria holobiont. This concrete evidence is a welcome contribution to the study of the Radiolaria photosymbiosis; however, the manuscript is quite difficult to follow as the aims and rationale for the experiments were not explained in detail, the methods are not explained to a broad audience, and the results were not placed in the context of radiolarian biology or previous research. In this context, we feel that the document requires revision for the following reasons:

We thank the reviewers for their in-depth assessment of our manuscript and the very helpful comments and suggestions.

General Comments:

There seems to be a conceptual disconnect between the authors' findings and their claims, although neither is clearly presented. The manuscript also lacks a clear biological rationale for the experiments. This is evident in the following ways:

• Collodaria (and other Radiolaria) are known heterotrophs, i.e., they phagocytose prey. This was only mentioned once, indirectly, in the discussion. Thus, if a collodarian were to acquire sugars through any other route than symbiont photoautotrophy, it would be heterotrophy, not osmotrophy. We suggest that the authors explain their choice of comparison here (osmotrophy vs. symbiont photoautotrophy) for the acquisition of carbon in the holobiont and place it in the context of Collodaria biology; that is, explain why a heterotrophy treatment was not employed.

– we thank the reviewer for this thoughtful observation and realize that the initial text was too much focused. To put the content into the required broader perspective we write now in the introduction part (Line 71) that radiolaria feed on small zooplankton, microorganisms, and detritus, and include references for this information. Many marine microorganisms are known to feed on dissolved organic carbon (Balch et al. 2023). From this perspective, we hypothesized that radiolaria can have developed additionally mechanisms to assimilate dissolved carbon through osmotrophy that complement phagocytotsis and symbiont photoautotrophy. We added this information to the introduction: "" - Lines 73-77.

For an experimental verification that indeed only osmotrophy and not phagocytosis is observed, in our approach, we tested the algal lysate for particles that carry organic metabolites and would qualify as substrates for phagocytosis. This showed that nearly no particle associated carbon is present in our treatment. This is now documented in the new Supplement – Appendix S2 and discussed in the manuscript (Lines 292-294). Our approach thus covers the radiolarian uptake of dissolved organic matter and allows to monitor the uptake into the holobiont.

• The osmotrophy treatment also requires some explanation. Why are B. nutricula lysates the chosen medium for assessing holobiont osmotrophy? A collodarian would never exist within a medium of its symbiont's lysates alone. Please describe in the text why algal lysates are an incomplete proxy for osmotrophy in Collodaria.

– we now re-structured the beginning of the results part to illustrate more clearly the scope of the experiments (Lines 162-170). We also extended the description of the generation of labeled dissolved organic metabolites in the chapter "Contribution of osmotrophy to Collodaria metabolism". There we mention now "These compounds will only be a proxy for the metabolites the holobiont is exposed to but include the most prevalent compound classes produced by phytoplankton." Lines 287-289.

Perhaps a clearer explanation of the aims and rationale for the algal lysate experiment (as well as the other experiments) would address these issues. For example, if the aim of the labeled algal lysate experiment were to test whether Collodaria are capable of taking up DMSP from the environment, then the experiment was appropriate, and the conclusions are well supported. If, on the other hand, the aim was to determine whether Collodaria obtain carbon primarily by photoautotrophy or by heterotrophy, the experiment is not appropriate. It was also unclear

whether and how the algal culture labeling was used as a control for interpreting the Collodaria data.

Thank you for this suggestion. We now explained the rationale behind each experiment in the beginning of the Result part in more detail. The numbered list (Lines 162-170) describes each of the experiments. In addition, we clarify the rationale of the algal lysate experiment at the beginning of the Results-chapter “Contribution of osmotrophy to Collodaria metabolism” - lines 284-289.

Incubation with lysate serves the purpose to mimic the uptake of DOM from the environment and trace the fate of specific compounds inside the holobiont. We used a lysate of algal symbiont to cover the metabolites typically produced by microalgae (DMSP, amino acids, lipids...) and to investigate their uptake. The use of the lysate of the symbiotic alga additionally allows to compare the taken up metabolites with those present in the free-living form of the symbiont. This allowed us in cases of metabolites with observed low label incorporation after incubation with the algal lysate to determine if this was caused by low incorporation in the algal metabolome by selective uptake and/or metabolism in the holobiont – we discuss these observations for amino acids (line 322), and ectoine (lines 534-537).

The work presented here confirms that collodarian symbionts are photosynthetically active, not that the collodarian host benefits from symbiont photosynthates. This is an important distinction because the former could be true of any alga stuck in the cytoplasm of any host, while the latter confirms the true nature of a photosymbiotic relationship. Please rephrase throughout the document to clarify this point. – We completely agree with this comment, for this reason we now write that the photosynthetic activity contributes to the metabolic pool of the holobiont, which includes both the host and the photosymbionts. We checked the manuscript carefully and corrected all parts that could imply any misinterpretation.

Please define all acronyms the first time they are used. A significant amount of jargon is used throughout the article; please clarify methods, results, and interpretations to appeal to a broader audience. It is currently written for those with an expertise in isotope labeling and/or metabolomics exclusively. – Thank you for pointing this out. We define all acronyms the first time they are used. We assume that most difficulties are caused by technical terms in the analysis of mass spectra and now also explain e.g. isotopologues, degree of labeling, and all others.

Specific Comments:

Line 20 – “that allows to investigate” should be “that allows us to investigate” or “that enables investigation into” - corrected

Lines 26-27 – “suggesting no significant contribution of osmotrophy to the holobiont’s carbon pool”; surely we do not expect significant carbon acquisition by osmotrophy in the open ocean? – We specify “to the holobiont´s carbohydrate pool” (Line 24). In fact, other metabolites like DMSP and betaines are indeed acquired through osmotrophy. Many marine microorganisms including microalgae are known to complement their metabolic pool through osmotrophy of dissolved organic carbon (Balch et al. 2023). From this perspective, although we find the comment valid in

terms of the probable low availability of dissolved organic carbon in oligotrophic waters, we show that that radiolaria can assimilate dissolved carbon through osmotrophy once inhabiting DOC rich environment.

Line 42 – "even if" should be "even though" - corrected

Line 51 – Should be "the proportion of the photosymbionts' contribution" - corrected

Line 62-66 – It's not obvious from this sentence that Collodaria is a subgroup of Polycystinea – please clarify this. We added this information.

Line 77 – What is the reference for studies suggesting photosynthetic carbon is less than 1%? – Swanberg and Harbison, The ecology of *Collozoum longiforme*, sp. nov., a new colonial radiolarian from the equatorial Atlantic Ocean. Deep Sea Res. Part A. Oceanogr. Res. Pap. 27, 715–732 (1980). – we added it to the sentence

Line 82 – Should be "but the more polar" - corrected

Line 88 – Clarify by stating "these symbiont pathways" rather than "these pathways" - corrected

Line 90 – How about "limited to monitoring isotope shuttling" - corrected

Line 93 – Remove the comma after "both" - corrected

Line 95 – Should be "allows us to characterize" or "that enables characterization of" - modified

Line 96 – Should be "we identify the involved" - corrected

Line 97 again – "allows us to compare" or "allows/enables comparison of"- modified

Line 100 – Remove "among others" - corrected

Line 109 – Should be "in the light to determine which metabolites are derived from" - corrected

Line 110 – Remove "allowed" - corrected

Line 111 – Change "allowed for monitoring of" to "to monitor" - corrected

Lines 111-112 – How does prolonged incubation in the light indicate substrate transfer in the holobiont? Substrate transfer implies transfer from symbiont to host, does it not? The aims and rationale of each labeling strategy should be explained in more detail. - Here, we meant the "accumulation" of metabolites, we corrected the text (Line 166-170).

Line 113 – Remove "allowed" - corrected

Line 122 – Change to "and/or" - corrected

Line 125 – What does degree of labeling measure, specifically? - Degree of labeling (DOL) shows which proportion of an analyzed compound's molecules contain an introduced stable isotope label. We added this information to the text (Line 186).

Line 126 – Please spell out GC-HRMS and CI on first mention - corrected

Line 128 – Please spell out EI on first mention - corrected

Lines 121-128 – It would be helpful to briefly describe the mass spec procedure, without jargon, so that the reader can visualize it without jumping to the methods first. – We added information to the introduction part (Lines 139-145) and expanded Figure 2 to illustrate the process.

Lines 121-123 – Please report the results in more detail, i.e., something like "we detected more mass signatures in the DMSP spectrum for the osmotrophic treatment than the control or the phototrophic treatment". Also explain which treatment is shown, why only these two metabolites are shown, and what the results for the other treatments were**.** – We added some clarification to this part (Lines 177-187). The comparison of different treatments follows in the next part, this paragraph together with figure 2 serve as a proof of concept demonstrating, that we can detect the label incorporation using high resolution mass spectrometry. We improved the clarity of the Fig. 2 and its caption.

Line 138 Fig. 2 legend – Please clarify which experimental treatment these spectra came from. Presumably the "osmotrophic assimilation" comes from the labeled algal lysate treatment, but the "photosynthetic assimilation" could come from any of the 3 labeled carbonate experiments. Which control is the control? Unlabeled cell lysates, or unlabeled culture medium? Also, presumably this is GC-HRMS data (not LC)? Which replicate samples were these spectra from, i.e., presumably you selected one nice spectrum to display, was there much variability among samples? We improved the clarity of the Fig. 2 and its caption: "Representative mass spectra of ^{13}C-enriched metabolites of the Collodaria holobiont. Incubation with ^{13}C-enriched algal lysate resulted in no detectable ^{13}C incorporation in glucose (**b**) but substantial incorporation into DMSP (**e**). Incubation with ^{13}C-bicarbonate resulted in substantial ^{13}C incorporation into glucose (**c**) and minor incorporation into DMSP (**f**). Panels (**a**) and (**d**) show the ^{12}C-bicarbonate control; the ^{12}C-lysate control yielded identical spectra to this control and therefore is not shown. Panels (**a**-**c**) display mass spectra of the depicted C3-fragment of derivatized glucose (*m/z* = 217.108) measured by GC-HRMS, panels (**d**-**f**) show $M+H^+$ ion of DMSP (*m/z* = 135.048) measured by LC-HRMS. y-axes show relative intensity compared to the most abundant ion; x-axes show the mass to charge ratio (*m/z*). TMS – trimethylsily."

Control is incubation with unlabeled medium, we didn't show the spectra from incubation with unlabeled lysate since it looks exactly the same as the one from incubation with unlabeled medium (it is also mentioned in the caption). We now clarify, that the spectra of the glucose fragment comes from GC-HRMS, of DMSP – from LC-HRMS. For representation we took random replicates, since spectra of metabolites from one setup looked very similar. We added all this information to the caption of the figure.

Line 144 – Add "the" before "holobiont's" - corrected

Line 147 – Remove comma after "by" - corrected

Line 149 – Add "the" before "holobiont's" - corrected

Line 153 Fig. 3 – How were these metabolites selected to display? Was there a threshold for degree of labeling that eliminated all other metabolites? Or was there a set of metabolites that you decided to target a priori? Here we displayed all the metabolites that, on one hand, were labeled at least in one of the experimental setups, and on the other hand could be identified and

confirmed with analytical standards. Not all compounds of the metabolome are accessible to mass spectrometric analysis. We now write in the figure legend "In this figure the labeled compounds are shown for which the structure could be unambiguously proven with a standard. "

Was the DOL 0 for the controls as well? This could be stated in the legend. The DOL for labeled compounds is normalized to the DOL of unlabeled compounds (note that every carbon containing compound has some degree of labeling because ^{13}C carbon is naturally present with 1.11% abundance). We added this information to the legend and describe the DOL determination in materials and methods section – lines 756-772.

Lines 148-151 – Is this statement intended to be agnostic about which partner is metabolizing the labeled C? Perhaps make this more explicit, otherwise it seems to be suggesting that the host is metabolizing the 13C compounds, which has not yet been demonstrated. We rephrased the statement: "Overall, the substantial ^{13}C incorporation in the Collodaria holobiont's carbohydrates from dissolved bicarbonate suggests that the photosynthetic activity of symbiotic algae contributes significantly to the holobiont's carbohydrate pool." Lines 224-227.

Line 171 – Change "along" to "alone" - corrected

Line 171-174 – Why would the DOL decrease for the hexoses? Is the holobiont preferentially metabolizing labeled hexoses? Or is the holobiont somehow fixing new unlabeled carbon into hexoses in the dark? Or am I misunderstanding DOL.

During the dark, carbohydrates that were freshly assimilated during light period are metabolized. Their pool can be fueled from carbohydrate storage molecules, such as starch, which is not necessarily labeled as it could have been accumulated during an earlier light cycle (not the one, when the ^{13}C was added). We added a clarifying sentence to the manuscript: "The decrease in freshly photosynthetically derived carbon in both hexoses, coupled with its increase in deoxyinositol, ribose, threonic acid, and gonyol indicates rapid metabolic turnover of the hexose pool in the dark. These patterns are consistent with hexoses being consumed and subsequently replenished by carbon sources independent of direct photosynthetic fixation – most likely through mobilization of previously stored compounds such as starch (Fettke et al. 2009, Tunçay et al. 2013)." Lines 254-260. The label incorporation into starch and its following mobilization was out of scope of this study and can be accessed in future works with longer labeling time and a starch hydrolysis step prior to metabolomic analysis.

Line 181 – It is not necessary to say "statistically significantly" because "statistically" is implied. It would be better to remove "statistically" in this context throughout the ms. corrected

Line 186 – How was it determined that the carbonates are photosynthetically derived? How does one determine the metabolic origin of a carbonate? Thank you for pointing this out, there was a typo, we meant carbon, we corrected it.

Line 189 – It seems that there is no way to be sure whether the labeled DMSP is incorporated by pinocytosis, phagocytosis, or true osmotrophy (i.e., importing solutes with transporters). Please include a sentence to explain why osmotrophy is more likely than heterotrophic modes of import,

since the title of this section states that it is osmotrophy. - We verified that the main source of assimilated metabolites is likely osmotrophic uptake of dissolved compounds by determining the amount of these metabolites in the supernatant and pellet of the lysate – this is now documented in the new Supplement – Appendix S2. We consider the metabolization of phagotrophically taken up algal particles to these metabolites to be very unlikely considering that nearly no particles were present in our preparation and the high label incorporation (especially for DMSP). We added a description and results of the comparative analysis of the supernatant and pellet of the lysate – Lines 292-294, 589-599. General considerations of the bioenergetic, membrane recycling, and vacuolar volume requirements that must be satisfied for pinocytosis-mediated uptake make this mechanism also unlikely (see e.g. Thamatrakoln and Kustka 2009).

Line 209 – Change “a half” to just “half” ” - corrected

Lines 212-215 – How long were the incubations for the free-living alga in ref 32? – In this reference, no incubation was performed. We now clarify in the text (Lines 313-317): “We addressed the contribution of photosynthetic activity of the symbionts and of osmotrophy of the host to the pool of metabolites shared by the Collodaria holobiont and free-living form of the algae (Appendix S4) by analyzing their DOL in each incubation. The investigated metabolites were identified in a previous study that compared the metabolic composition of the holobiont and the free-living algae (Nikitashina et al. 2025).”

Also, could there not be an explanation besides slower metabolism in the holobiont? Perhaps the host is taking most of the fixed carbon and metabolizing it along different pathways than what the alga would use it for? Particularly consider that many amino acids are likely to be synthesized by the alga and not the host. Since we didn’t detect any label incorporation in these metabolites neither after the incubation with labeled bicarbonate nor after the incubation with the labeled lysate, we concluded that if these routes are contributing to the assimilation of these metabolites, then the metabolism is slow and could not be detected within the time scale of performed incubation. We now discuss possible options that could be responsible for the absence of the label incorporation: “Alternatively, the lack of ^{13}C incorporation into these metabolites following incubation with labeled bicarbonate may reflect their rapid turnover by the host or symbiosis-induced modifications of algal metabolic pathways that suppress their production.” Lines 325-328.

Line 217 – Rather than “obtains” perhaps say “incorporates 13C into” We changed obtains to produces, this illustrates the process better

Line 220 – Change to “glucose, fructose, and derived metabolites” - corrected

Line 221 – Change “whereas” to “conversely” or else link this sentence to the previous with a comma after “metabolites”. - corrected

Lines 237-239 – How do we know that Collodaria cannot survive without photosynthetically active symbionts if we cannot cultivate them? – Previous study showed that when kept in the darkness (therefore without photosynthetically active symbionts), the survival of photosymbiont-bearing Collodaria was significantly reduced. We rephrase the sentence accordingly. Lines 360-362.

Line 240 – Is “translocation” really the best word? It suggests carbohydrate transporters, whereas your data concerns metabolites not transporters. – We rephrased it to: “Here, we describe a set of molecules that connect the partners in this symbiotic system by different pathways.” Lines 374-376.

Line 241 – Please reword. “Could be achieved” sounds like it might happen in the future, when you have already achieved it in the past. Also, what is a “compound-resolved analysis”? We rephrased this paragraph – Lines 360-383.

Line 243 – I think “metabolism” would be more accurate than “shuttling” in this context? Better still, remove the first paragraph. It communicates nothing of substance and has several slight inaccuracies. - corrected

Line 257 – Remove the “a” after “accumulation” - corrected

Lines 257-261 – It would be helpful to describe how the accumulation of photosynthetically derived glucose in photosymbiotic hosts was determined. We added this information in the text: “based on evaluations of 13C or 14C incorporation into host tissues after incubation with labeled bicarbonate” Lines 406-408.

Line 263 – Please include a description of what these molecules are, what they do, and why they are important (this is not common knowledge). – we added this information: “Inositols are cyclic sugar alcohols found in eukaryotic cells, including marine microalgae and protists. They function as compatible solutes to protect cells from osmotic stress and inositol phosphates mediate cellular signaling.” Lines 416-419.

Line 271 – Please describe why these compounds are thought to indicate that they originate from algal photosynthesis – We clarified the sentence: “Assimilation of photosynthetically derived ^{13}C carbon was also detected in methyl palmitate and palmitoyl-glycero-phosphocholine, indicating that these metabolites originate from photosynthetic activity of the microalgae as well.” Lines 429-432.

Line 275 – Change to “the jellyfish photosymbiosis” - corrected

Lines 316-321 – Heterotrophic feeding is not the same as osmotrophy, especially not in a lineage that is known to consume prey as large as copepods. While your hypothesis is sound given what we know about heterotrophy, generally, it does not follow that metabolites acquired via osmotrophy would be the same metabolites acquired via heterotrophic feeding.

We fully agree and clarified this part accordingly: “Our results for Collodaria photosymbiosis support these observations from corals and highlight a potentially common mechanism in marine photosymbiotic systems. Earlier studies on heterotrophy in photosymbiont-bearing radiolaria showed that they can feed on various prey; however, this plays only a secondary role in their overall fitness. In contrast, illumination is essential for their survival (Swanberg and Anderson 1985, Swanberg and Caron 1991). Consistent with the patterns observed in our study, this suggests that while heterotrophy may not be the primary source of energy for radiolaria, together with osmotrophy, it could still provide access to essential nitrogen- or phosphorus-rich metabolites.” Lines 510-517.

Line 337 – Remove parentheses around "filled to the top of the rim" and remove following comma - corrected

Line 387 – I believe it should be "Villefranche-sur-Mer" - corrected

Line 405 – Better would be "A minimum of" or "At least" - corrected

Line 409 – Fix typo to "concentration" - corrected

Line 425 – Change "till" to "until" - corrected

Line 425 – Does this mean 7 to 9 colonies were prepared in each setup in each replicate? Or divided among setups so that each setup only had 2-3 colonies? – 7 to 9 colonies were prepared for each replicate – clarified in the text – lines 639-641.

Line 426 – Does this mean that each setup had the same number of colonies across replicates, or each replicate had the same number of colonies across setups? – Each replicate had the same number of colonies across setups

Line 428 – Fix typo C-labeled - corrected

Line 429 – "culture" should be "cultures" - corrected

Line 433 – Change "till" to "until" - corrected

Line 441 – Change "till" to "until" - corrected

Line 441 – Please clarify whether the 11 colonies were divided among the two treatments for each replicate, or whether each treatment had 11 colonies in all replicates. – each replicate contained 11 colonies – clarified in the text – line 662.

Line 455 – Remove "a" before "room temperature" - corrected

Line 455 – Change "spitted" to "split" - corrected

Line 523-524 – Please check this sentence. The comma is in the wrong place and the sentence is incomplete. – corrected – "Consecutive isotopologues were identified as peaks differing by 1.00335 Da (± 5 ppm)." – Lines 748-749.

Line 566 – What does "downstream processing" mean, please provide specifics – Thank you for pointing this out, by downstream processing we meant the assembly of the image itself, we clarified this point – "Images of six regions from one focus plane were taken and assembled to cover the whole colony." Lines 790-791.

Reviewer #2 (Remarks on code availability):

We cannot fully evaluate the code as it is beyond our areas of expertise.

Reviewer #3 (Remarks to the Author):

Review of Vera Nikitashina and Georg Pohnert “Partitioning of Carbon Metabolism Between Photosynthesis and Osmotrophy in Collodaria Photosymbiotic Holobionts”

Overall comment: I enjoyed reading this ms. It is a very addition to many questions that were investigated ~50 years ago, but now using modern approaches. Aside from improving our knowledge of radiolarian ecology, it will be very useful for the understanding of symbioses overall. I do not have many criticisms about the methodologies used here; it is pretty straightforward, as the results. However, I was a bit frustrated with the discussion section. The authors mentioned many different elements, but only provided a glimpse of their impact on the holobiont functioning. I have made several specific comments below to indicate this lack of discussion. Finally, the introduction is lacking details, which can be easily added.

Overall, with a bit of polishing and editing, it will be a good and useful ms.

I made specific comments below.

We thank the reviewer for the careful reading of our manuscript and useful comments. Below we provide answers to each comment.

Abstract

Line 15- I would write “can contribute”. There are only a few reports of them contributing to primary production. - corrected

Introduction

Line 39- I would add a general reference to marine photosymbioses (perhaps remove one of the many references to photosymbioses with protists?). - corrected

Line 42- What is meant by ‘central for the global photosynthesis budget’? The same goes for ‘roles of photosymbiotic’? Please provide clearer elements. – we rephrased this part to: “Despite their substantial role in primary production and the marine food web, the mechanisms governing photosymbiotic relationships remain poorly understood. “ Lines 46-48.

Line 53- photosynthetic ‘plastids’? – corrected to “photosynthetic plastids”

Line 53- ‘carbon pool’ = inorganic? Organic? – corrected to “organic carbon”

Line 58- The transition from the previous paragraph is odd. We are jumping from jellyfish to radiolarians? – we added a sentence “However, the role of the photosymbiont in photosymbioses of planktonic radiolaria remains poorly understood.” as a transition. Lines 64-65.

Line 59- ‘Four out of five orders’. Most readers will be ignorant about radiolarian classification. I would either name the five orders, mention the only non-mixotrophic order, and/or at least provide a reference for this statement. – we modified this part to make it easier to understand for the broader audience: “Radiolaria feed heterotrophically on small zooplankton, microorganisms, and

detritus. Many planktonic radiolaria harbor photosymbiotic microalgae and are therefore considered as mixotrophs (Anderson 1976, Swanberg and Harbison 1980, Swanberg and Anderson 1985, Zettler et al. 1997, Stoecker et al. 2009, Decelle and Not 2015, Lampitt et al. 2023)." Lines 70-73.

Line 62- It is quite unusual to see mixotrophy mentioned only at this point. Mixotrophy should be mentioned earlier (in particular since you mention kleptoplastidy with your sea-slugs). – we added this detail earlier in the text: "The general model of photosymbiosis suggests that the photosymbiont provides organic compounds, fueled by photosynthesis, to an otherwise heterotrophic partner, thereby making such associations mixotrophic" Lines 54-56.

Line 63- Again, Collodaria appear out of nowhere! You should probably introduce them with one sentence before (at least say that it's one of the mixotrophic orders you mentioned before). We added the following sentence: "Collodaria is an order of Radiolaria (subgroup of Polycystinea) that includes photosymbiont-bearing species." Line 78.

Line 64- radiolaria comes with a capital R. - corrected

Line 65- I would separate this sentence into two; it is pretty long and hard to follow. – modified to "Photosymbiotic Collodaria are the most abundant radiolaria in the upper 100 m of the water column (Biard et al. 2016) and can harbor hundreds to thousands of microalgae in their cytoplasm (Fig. 1). Because the population density of microalgae is higher within the host compared to the surrounding environment, photosymbiont-bearing Collodaria represent hot spots of primary production (Anderson 1976, Swanberg and Harbison 1980, Caron et al. 1995, Decelle et al. 2021)." Lines 80-85

Line 71- Is it really a matter of debate, or rather something that we do not know? – we changed this to "not fully understood", which is the best description for the current state of knowledge for this subject. Line 89.

Line 75- 'other studies' Which one?!?! Here we reference the paper from Swanberg and Harbison, 1980, we added the reference in the end of the sentence.

Results

Figure 2. I am not used to seeing such graphics. There is no explanation about 'mlz'? What does it stand for? Also, your y-axis says 'relative abundance'. I am guessing that it is expressed as %. If so, why would you have several 100% bars? Please clarify the figure and the caption. – *m/z* stands for mass to charge ratio which is used to identify ions of different molecular weight; y-axis represents relative abundance (relative intensity) of each ion – an ion with most intensive signal in the selected range has per definition 100% intensity, intensities of all other ions are represented relatively to the intensity of the most intensive ion. If being very precise, only one ion at each graph has 100% intensities, other ions can be very close, but still not 100%. We clarified the figure caption accordingly: "Representative mass spectra of ^{13}C-enriched metabolites of the Collodaria holobiont. Incubation with ^{13}C-enriched algal lysate resulted in no detectable ^{13}C incorporation in glucose (**b**) but substantial incorporation into DMSP (**e**). Incubation with ^{13}C-bicarbonate resulted in substantial ^{13}C incorporation into glucose (**c**) and minor incorporation into DMSP (**f**). Panels (**a**) and (**d**) show the ^{12}C-bicarbonate control; the ^{12}C-lysate control yielded identical spectra to this

control and therefore is not shown. Panels (**a**-**c**) display mass spectra of the depicted C3-fragment of derivatized glucose (*m*/*z* = 217.108) measured by GC-HRMS, panels (**d**-**f**) show M+H$^+$ ion of DMSP (*m*/*z* = 135.048) measured by LC-HRMS. y-axes show relative intensity compared to the most abundant ion; x-axes show the mass to charge ratio (*m*/*z)*. TMS – trimethylsily." We also modified the figure and included a color scheme to make it easier to understand.

Line 126- What are 'CL' and 'EL' modes? - 'CI' and 'EI' modes stand for chemical ionization and electron ionization, we added the explanation in the text

Line 132- You have defined before who 'B. nutricula' are (and cannot use the abbreviation then). – the species name is now written out the first time it is mentioned and later abbreviated.

Line 176- This is the first time you mention the 'metabolome'. You should define this above in the introduction. The same goes for 'osmotrophy' later. – We now introduced both terms in the introduction: "Such an ability to obtain nutrients by taking up dissolved organic compounds from the surrounding environment – known as osmotrophy, is common among unicellular plankton" – Lines 75-77, "By analyzing $_{13}$C incorporation into the holobiont's endometabolome (the complete set of low-molecular weight metabolites within the holobiont), we identify the specific metabolites involved in the photosymbiosis and determine the contribution of each mechanism of metabolite acquisition to the holobiont's metabolic pool." Lines 148-152.

Line 192- What is an 'algal lysate'? – it's a lysate of cultured *B. nutricula*, we added this information to the description of the experiment: "…we also investigated ^{13}C enrichment in metabolites that results from collodarian osmotrophy upon exposure to labeled dissolved organic metabolites. To obtain the labeled metabolites, which mimic compounds released from co-occurring microalgae in the field, we grew *Brandtodinium nutricula* – the free-living form of the Collodaria photosymbiont (Probert et al. 2014) in ^{13}C-bicarbonate enriched medium and extracted the labeled metabolites from the algae." (line 282-287).

Line 208- Monitored for how long? I think that it is important to mention the incubation time since you are arguing that it was not enough to detect accumulation. – There was a typo – we didn't monitor, we analyzed the DOL for these compounds after incubation within each setup. We clarified the sentence: "We addressed the contribution of photosynthetic activity of the symbionts and of osmotrophy of the host to the pool of metabolites shared by the Collodaria holobiont and free-living form of the algae (Appendix S4) by analyzing their DOL in each incubation." Lines 313-316.

Discussion

Line 238- I would provide a reference to support 'uncultivable' – we provided a reference at the end of the sentence.

This first paragraph seems like a conclusion rather than the first paragraph of a discussion section (which would be the second). We rephrased the paragraph to be more fitting for the beginning of the discussion: "The absence of photosynthetically active algal symbionts significantly reduces the survival of the photosymbiont-bearing Collodaria (Swanberg and Anderson 1985). However, because Collodaria are uncultivable, it is still not fully understood how the photosynthetic symbionts support them (Ohtsuka et al. 2015). Here, we describe a set of molecules that connect

the partners in this symbiotic system by different pathways. Overall, our analysis of the DOL of the Collodaria holobiont metabolome upon incubation with labeled bicarbonate or labeled algal lysate showed that the holobiont produces glucose and fructose during photosynthesis (Fig. 4). During the dark period, both sugars are converted to other metabolites and their pool is being replenished from sources other than direct photosynthetic fixation. Prolonged incubation with labeled bicarbonate in the light results in DOL-increase in glucose, fructose, and derived metabolites. Osmotrophy serves as a source of osmolytes but not sugars or amino acids." Lines 360-383.

Line 266- 'role in host-photosymbiont signaling'. Unclear, be more precise. – we rephrased this part to: "The roles of these inositols, however, were not defined (Burriesci et al. 2012, Matthews et al. 2018). It was hypothesized, that they play a role in host-photosymbiont signaling, recognition and dysfunction, but a prove was not documented." Lines 421-424.

Line 282- If ribose and deoxyinositol are continuously produced, please explain what roles do they play in the holobiont?. The same goes for your sentence at line 298. We added this information to the text: "Together with the decrease of DOL for these two sugars, a significant increase of the DOL for ribose, putatively identified deoxyinositol, and threonic acid was observed (Fig. 3a). The increase of the DOL for these metabolites during the incubations suggests that they are produced from the sugars initially accumulated during photosynthesis. The phosphorylated form of ribose is produced from glucose via the pentose phosphate pathway and plays key roles in cellular energy metabolism. It also provides the carbon backbone for nucleotide and nucleic acid biosynthesis (Stincone et al. 2015). Its assimilation from photosynthetically derived glucose in Collodaria photosymbiosis implies a significant impact of photosynthetic activity for the holobiont´s energy metabolism. Threonic acid, in turn, is formed form from ascorbic acid, which itself can be synthesized from fructose (Kanehisa and Goto 2000, Ford et al. 2024). Photosynthetically derived threonic acid has been reported in coral photosymbioses (Matthews et al. 2018), and in photosymbiotic systems it may be associated with coping with oxidative stress resulting from symbiont photosynthetic activity. There are few reports on the biosynthesis and physiological role of putatively identified deoxyinositol. By drawing parallels with other inositols, it is possible that deoxyinositol is synthesized from glucose via myo-inositol and may serve osmolytic and antioxidative functions (Klages et al. 1999, Kanehisa and Goto 2000, Yancey 2005)." Lines 443-460.

Line 303- Technically speaking, you did not observe any feeding. You speculate that lysates were transferred to the host, but the mechanisms are unknown. – we rephrased to: "…we also observed an osmotrophic uptake of an algal lysate by the Collodaria holobiont." Line 490-491.

Line 316- I would turn your sentence saying that you support observations made for corals. I think that you can simply say that the explanations provided for coral symbioses could the same for Collodaria (and open the door to several common mechanisms in photosymbiosis overall). – we rephrased this part to: "Our results for Collodaria photosymbiosis support these observations from corals and highlight a potentially common mechanism in marine photosymbiotic systems." Lines 510-512.

Line 321- Here, you tested only heterotrophic feeding through the scope of osmotrophy. Are there any elements from past literature (e.g., Anderson and Swanberg's experiments back in the 80s). – we added some information from the literature: "Earlier studies on heterotrophy in photosymbiont-bearing radiolaria showed that they can feed on various prey; however, this plays only a secondary role in their overall fitness. In contrast, illumination is essential for their survival (Swanberg and Anderson 1985, Swanberg and Caron 1991). Consistent with the patterns observed in our study, this suggests that while heterotrophy may not be the primary source of energy for radiolaria, together with osmotrophy, it could still provide access to essential nitrogen- or phosphorus-rich metabolites." Lines 512-517.

Line 322- What would be the interest for Collodaria to uptake DMSP? What is the role of DMSP in symbiosis? Was it not suggested to play an antioxidant role in radiolarian symbioses? Isn't DMSP produced by the symbionts and their production boosted by the host (see work from Decelle's lab) to cope with high light stress? – DMSP plays an important role as an osmoprotectant, antioxidant, and signaling molecule in various marine microorganisms and coral photosymbiosis (Sunda et al. 2002, Raina et al. 2013, Nikitashina and Pohnert 2023). Similar to coral photosymbiosis, in radiolaria photosymbiosis may play all these roles. We agree with the comment about increased DMSP content in radiolaria photosymbiosis, however, to the best of our knowledge, its exclusive production by the algal symbiont has never been documented and was assumed due to the lack of other evidence. In the study investigating DMSP content in Collodaria photosymbiosis, the origin of this metabolite and possible contribution of the host to its pool was still open. In this study, together with photosynthetically derived DMSP, we observed that the Collodaria holobiont assimilates significant amounts of DMSP from the surrounding water, which demonstrates that osmotrophy contributes to its pool in the holobiont. We added information about DMSP and its possible roles and origin in the Collodaria photosymbiosis to the introduction (Lines 112-123): "Together with photosynthate translocation, metabolism of specialized metabolites (i.e. compounds that are not directly required for basic growth, development, or reproduction, but instead serve ecological and adaptive functions) can play a fundamental role in the holobiont-functioning. In this context, radiolaria photosymbioses are potentially important sources of marine dimethylsulfoniopropionate (DMSP) (Decelle et al. 2012, Gutierrez-Rodriguez et al. 2017). As a sulfur-containing compound and a precursor of the volatile dimethyl sulfide, DMSP contributes significantly to the global sulfur cycle and is primarily produced by marine phytoplankton and pelagic bacteria (Kirst et al. 1991, Yost and Mitchelmore 2009, Bullock et al. 2017, Jackson and Gabric 2022). In radiolarian photosymbioses, a significantly elevated DMSP content compared to free-living algae has been reported, however, the relative contribution of each symbiotic partner to the DMSP pool remains unclear (Decelle et al. 2012, Gutierrez-Rodriguez et al. 2017)." And in the discussion we added (Lines 524-533): "DMSP plays an important role as an osmoprotectant, antioxidant, and signaling molecule in various marine microorganisms and coral photosymbiosis (Sunda et al. 2002, Raina et al. 2013, Nikitashina and Pohnert 2023). Although previously detected in higher concentrations in radiolaria photosymbioses than those calculated for free-living algae, its origin has never been determined. Our observation of a high proportion of labeled DMSP after incubation with labeled algal lysate, together with photosynthetic contribution to the pool of this compound suggest that Collodaria can obtain this metabolite from the environment and the photosymbionts. The dual origin of this molecule may explain its higher-than-expected concentration in Collodaria reported earlier

(Gutierrez-Rodriguez et al. 2017) and indicate Collodaria photosymbiosis as an important sink of environmental DMSP.”

Reviewer #4 (Remarks to the Author):

I co-reviewed this manuscript with one of the reviewers who provided the listed reports. This is part of the Nature Communications initiative to facilitate training in peer review and to provide appropriate recognition for Early Career Researchers who co-review manuscripts.

Reviewer #5 (Remarks to the Author):

General comments:

The manuscript entitled “Partitioning of Carbon Metabolism Between Photosynthesis and Osmotrophy in Collodaria Photosymbiotic Holobionts” by Nikitashina and Pohnert reported the metabolic dependencies between Collodaria hosts and dinoflagellate symbionts using isotopic labeling techniques. Radiolarians, particularly the colonial group Collodaria, are ubiquitous and abundant marine protistan plankton that significantly contribute to the global carbon cycle. It is therefore crucial to develop a comprehensive understanding of ecological strategies, such as photosymbiosis, in order to gain a more profound insight into the subject. Their results demonstrated that glucose and fructose are the major primary metabolites synthesized by the symbiont through photosynthesis. In contrast, osmolytes such as DMSP and betains are of environmental origin, rather than being derived from the photosynthetic pathway. I would like to note that I am not an expert in metabolomics or isotope labeling approaches; therefore, I cannot comment extensively on the technical aspects of the analysis. From the viewpoint of plankton photosymbiosis in general, I would like to applaud their achievement, demonstrating what is produced and transferred among the symbiotic system. The results are sound, although their major finding—namely, sugar synthesis by the symbionts and its subsequent transfer to the host—is something that can be predicted in general. The DMSP accumulation path is interesting; however, I think further discussion is required for this part. Please find the comments below.
We thank the referee for his / her assessment of the manuscript and the helpful comments that allowed us to focus our work and to include novel aspects.

・Comments on DMSP

High DMSP concentration in holobiont is generally attributed to their symbionts, but in the case of radiolarians, it has been shown that symbiont contribution alone cannot explain the observed high concentration. This kind of background needs to be explained in the introduction; otherwise, why the authors are focusing on DMSP would not be easily understood by the audience. This study clearly showed that DMSP of holobionts is derived from both symbiont photosynthesis and from the environment, but the contribution is far higher for the latter, although another possible pathway, the ability of DMSP synthesis by the host itself, cannot be discussed from this experiment. The higher contribution of DMSP incorporation from the environment indicates that

Collodaria photosymbiosis is a sink of DMSP. I think this view is important since the size of the environmental pool of DMSP that will be decomposed to generate DMS is reduced. If my understanding is correct, this would also be a strong point of this study. – This comment is in agreement with Reviewer 3. As mentioned above, we added information about DMSP and its possible roles and unresolved origin in the Collodaria photosymbiosis to the introduction (Lines 112-123): "Together with photosynthate translocation, metabolism of specialized metabolites (i.e. compounds that are not directly required for basic growth, development, or reproduction, but instead serve ecological and adaptive functions) can play a fundamental role in the holobiont-functioning. In this context, radiolaria photosymbioses are potentially important sources of marine dimethylsulfoniopropionate (DMSP) (Decelle et al. 2012, Gutierrez-Rodriguez et al. 2017). As a sulfur-containing compound and a precursor of the volatile dimethyl sulfide, DMSP contributes significantly to the global sulfur cycle and is primarily produced by marine phytoplankton and pelagic bacteria (Kirst et al. 1991, Yost and Mitchelmore 2009, Bullock et al. 2017, Jackson and Gabric 2022). In radiolarian photosymbioses, a significantly elevated DMSP content compared to free-living algae has been reported, however, the relative contribution of each symbiotic partner to the DMSP pool remains unclear (Decelle et al. 2012, Gutierrez-Rodriguez et al. 2017)." And in the discussion we added: "DMSP plays an important role as an osmoprotectant, antioxidant, and signaling molecule in various marine microorganisms and coral photosymbiosis (Sunda et al. 2002, Raina et al. 2013, Nikitashina and Pohnert 2023). Although previously detected in higher concentrations in radiolaria photosymbioses than those calculated for free-living algae, its origin has never been determined. Our observation of a high proportion of labeled DMSP after incubation with labeled algal lysate, together with photosynthetic contribution to the pool of this compound suggest that Collodaria can obtain this metabolite from the environment and the photosymbionts. The dual origin of this molecule may explain its higher-than-expected concentration in Collodaria reported earlier (Gutierrez-Rodriguez et al. 2017) and indicate Collodaria photosymbiosis as an important sink of environmental DMSP." (Lines 524-533).

Whole through the manuscript, quantitative consideration is scarce. I understand that the experiments employed in this study does not allow quantification of the flux of the metabolites, but at least the information on the content of metabolite in the lysate needs to be provided to interpret the results. If the DMSP concentration in the incubation medium was too high compared to the natural environment, the osmotrophic incorporation of DMSP demonstrated in this study may not be a realistic pathway. – we agree with this comment and included these aspects in the text: "The concentration of the lysate was selected to correspond to cell counts of the algae that are ten to a hundred times lower than the symbiont density within a colony (Swanberg and Harbison 1980, Mansour et al. 2021) and the density of an exponentially growing algal culture, respectively." – Lines 289-292.

As we also indicate below, due to comparatively low amount of available biological material collected from the environment, we didn't have an opportunity to test several lysate concentrations, and had to choose one, which, on one hand was comparatively low to avoid harmful effects for the colony, but high enough to detect label incorporation after the incubation.

- Incubation with labeled metabolites

In this study, the authors used lysates of labeled symbiont (free-living B. nutricula culture) in experiment iv. It is unclear to me whether this mimics the condition of the symbiont digestion process, or the uptake of dissolved organic matter (DOM) from the surrounding environment. I assume that the primary purpose is the latter. If so, why use the specific symbiont rather than common microalgae such as Isochryisis or Thalassiosira? Or does this experiment serve both intentions? Please clarify. – We agree that this setup only partially mimics the natural conditions because in nature the DOM originate from mixed microalgal and bacterial population present. However, we don't see a benefit in utilizing any other species, because it still will not solve a problem of complex species composition in the environment. However, it would make the comparisons of labeled metabolomes less straightforward. We now added a clarifying section in the description of the experiment: "...we grew *Brandtodinium nutricula* – the free-living form of the Collodaria photosymbiont (Probert et al. 2014) in ^{13}C-bicarbonate enriched medium and extracted the labeled metabolites from the algae. These compounds will only be a proxy for the metabolites the holobiont is exposed to but include the most prevalent compound classes produced by phytoplankton." Lines 285-289.

- Class of compounds

Including me, for those who are not familiar with metabolite names, it is very helpful if we can find compound class information elsewhere in the manuscript. The simplest way is to add that information in Figure 3, or add a table of the metabolite discussed in this study. Please consider this addition. We added a table with information on compound classes for targeted metabolites to the supplement - Appendix S4.

- Not labeled or not detected

In Figure 3, ND (not detected) is used for DOL level. Does it mean 0% DOL (the unlabeled compound was detected) or that even the unlabeled compound was not detected? Presence or absence information is also important, so please clarify. – We clarified the caption of Figure 3 and defined ND: "Degree of labeling (DOL) for labeled metabolites of the Collodaria holobiont after incubation with ^{13}C-bicarbonate and ^{13}C-enriched algal lysate, respectively. (**a**) Metabolites that incorporated ^{13}C exclusively through photosynthesis, (**b**) metabolites that incorporated ^{13}C by both, photosynthesis and osmotrophy, and (**c**) metabolites that acquire ^{13}C labeling exclusively by osmotrophy. The final ^{13}C-DOL values were corrected for natural ^{13}C abundance by normalization with DOL values of non-labeled control samples. In this figure the labeled compounds are shown for which the structure could be unambiguously proven with a standard (exception deoxyinositol was identified based on a library hit). Error bars represent the standard deviation; LysoPC – palmitoyl-glycero-phosphocholine, DMSP – dimethylsulfoniopropionate; * – $p \leq 0.05$, ** – $p \leq 0.01$, *** – $p \leq 0.001$ (unpaired *t* test), ND – metabolite present, but no label incorporation detected."

・Interpretation of the difference between the photosynthesis-derived metabolite and the algal lysate

Comparing the difference in DOL between the metabolite of holobiont incubated with 13C-bicarbonate (products of in hospite B. nutricula) and labeled algal lysate (products of free-living B. nutricula), as shown in Appendix S2, many metabolites are labeled only for the free-living algal lysate, although it was also originally from photosynthetically assimilated carbon. In my understanding, it indicates that (1) the metabolism is altered when they are in symbiotic states, (2) the metabolite was quickly utilized by the host, (3) the metabolite synthesis was too slow to detect the label, or (4) the metabolite pool is so large that the relative proportion of newly synthesized labeled metabolite was too low. In the discussion, it seems the possibility (3) alone is considered (L212-215). Please discuss other possibilities. Thank you very much for this comment. We included possibilities (1) and (2): "Alternatively, the lack of ^{13}C incorporation into these metabolites following incubation with labeled bicarbonate may reflect their rapid turnover by the host or symbiosis-induced modifications of algal metabolic pathways that suppress their production." (Lines 325-328). However, in our opinion, the possibility (3) already includes possibility (4), because even if the metabolite pool is very large, rapid synthesis of the metabolite would make it distinguishable, as we observe it for other metabolites, for which we could detect the label incorporation after incubation.

I checked their previous publication, Nikitashina et al. (2025, ISME J), and found that Taurine was not detected in the holobiont, but was detected in free-living. This may be the case of (1) or (2) (presence or absence information is also needed). In addition, in Nikitashina et al. (2025), Tyrosine was absent in the free-living symbiont, which is inconsistent with the present result (Appendix S2). Please explain/discuss it. – overall tyrosine has a very low content in both *B. nutricula* and Collodaria samples. In Nikitashina et al. (2025), only top 200 most intense metabolites were processed and annotated, and tyrosine was not present within these 200. For the present study we used another processing workflow focused on labeled compounds, and since there was only a low amount of the labeled compounds present in the algal lysate, tyrosine is now listed. It had similar low intensity as reported in the previous study. This is now mentioned in the Appendix S4. Taurine was previously detected only in the free-living algae, consistent with these results, we could detect ^{13}C labeled taurine in the algal extracts and didn't detect this compound among metabolites of the holobiont.

・Phagotrophy

Since the target species are heterotrophic, knowledge of their natural food/prey needs to be explained. Do they eat living plankton or detritus, or just perform osmotrophy? – information about radiolaria feeding behavior is now included in the introduction– lines 70-73: "Radiolaria feed heterotrophically on small zooplankton, microorganisms, and detritus. Many planktonic radiolaria harbor photosymbiotic microalgae and are therefore considered as mixotrophs (Anderson 1976, Swanberg and Harbison 1980, Swanberg and Anderson 1985, Zettler et al. 1997, Stoecker et al. 2009, Decelle and Not 2015, Lampitt et al. 2023)."

· Threonic acid

Compounds whose DOL increased after 12h-12h light-dark condition compared to 12h light condition were interpreted to reflect rapid hexose metabolism into those compounds (ribose, deoxyinositol, gonyol). In terms of threonic acid, it also increased after light-dark cycle, but not discussed in the discussion. Besides, the result of threonic acid for 12h light incubation in Figure 3 is missing. Does it mean "ND" or not detected at all? Please clarify, and include the discussion on this compound in the main text. –Thank you for pointing this out. ND – metabolite present but no label incorporation detected, is now explained in the legend (see comment above). There was a typo in the figure 3 – there should be ND for threonic acid for 12h light incubation, we corrected it. We also included information for this metabolite in the result and discussion parts – Lines 253, 256, 445, 470, and 452-456: "Threonic acid, in turn, is formed form from ascorbic acid, which itself can be synthesized from fructose (Kanehisa and Goto 2000, Ford et al. 2024). Photosynthetically derived threonic acid has been reported in coral photosymbioses (Matthews et al. 2018), and in photosymbiotic systems it may be associated with coping with oxidative stress resulting from symbiont photosynthetic activity."

Other points

Throughout the text: 13C-carbonate should be 13C-bicarbonate. Carbonate and bicarbonate are different. – Thank you for pointing this out, we correct it throughout the manuscript.

L209: "DOL for these compounds" --> needs to be more specific. – we added a table with targeted compounds in supplement materials (Appendix S4)

L277: Lipid involvement in pelagic photosymbiosis was demonstrated by LeKieffre et al. (2018). This should be cited here. – Thank you for this suggestion, we added this information and reference to the text (Lines 436-438): "Furthermore, assimilation of photosynthetically derived carbon in lipid droplets was described for benthic foraminifera bearing photosynthetically active sequestrated algal chloroplasts (LeKieffre et al. 2018)."

L321: Need references. Many previous studies showed this idea on N and P requirement in the photosymbiotic system. – We have added a reference for the study showing this phenomenon in corals (Kubiak and Kloc 2018). Here we meant that our results support these observations and draw parallels between different symbiotic system. We rephrased this part to clarify this: "Therefore, photosymbiont-bearing corals obtain nitrogen- and phosphorus-containing metabolites from other sources than photosymbionts (Kubiak and Kloc 2018). Our results for Collodaria photosymbiosis support these observations from corals and highlight a potentially common mechanism in marine photosymbiotic systems." (Lines 503-512).

L399-400: Please provide the temperature of the ambient seawater. We added the temperature (Lines 614).

L434: Please explain the reason for the final concentration of algal lysate (3.5 x 104 cells mL-1). Is it expecting the natural environment or mimicking the phagotrophic condition? – We added the

rationality behind chosen lysate concentration (Lines 651-654): “This concentration is approximately ten to a hundred times lower than the symbiont density within a colony (Swanberg and Harbison 1980, Mansour et al. 2021) and the density of an exponentially growing algal culture, respectively, and was chosen to avoid any potentially harmful effects of the lysate on the colony.” Due to demand on high colony number per experiment and comparatively low amount of available biological material collected from the environment, we didn’t have an opportunity to test several lysate concentration, therefore we used this one, which, on, one hand was comparatively low to avoid be potential harmful for the colony, but high enough to detect label incorporation after the incubation.

L436: Why was this experiment terminated after 22h, not 24h? In the three experiments using NaH13CO3, when the specimens were transferred to FSW, it was defined as the end of the experiment, so in the forth experiment case, the experimental duration was 22h. Figure 4d needs to be corrected accordingly. – we used this time window to have the overall incubation time equal for all setups, also based on the daytime when the experiments were setup and terminated. This was done to make comparisons across the setup more precise, because additional incubation hours could influence colonies’ fitness and physiology. We corrected figure 4d accordingly.

Figure 3: Thrigonelline --> Trigonelline - corrected

Figure 4: 13CO2 is not correct. It should be NaH13CO3. This kind of schematic illustration is fine, but this one is not so much informative (the information in this figure can easily be understood from the text). Alternatively, I would suggest to include a figure of experimental design (the experimental condition and flow of the analysis), in addition to this result illustration. Thank you for the suggestion, we modified and corrected the figure 4 accordingly.

Reviewer #5 (Remarks on code availability):

Readme file is not available.

I could not run the code since the data is not yet accessible from the data repository. I suppose when the paper is published, the data will be open in the database (MetaboLights ID: MTBLS12592 is provided, but not accessible at this moment). So I cannot evaluate the correctness of the code. – Yes, the study will be publicly available when published, for now the data should be accessible following the link: https://www.ebi.ac.uk/metabolights/reviewerecb0e821-8d0d-4c1d-9ee0-b0d61da157fe, we also provided this link in the submitted version of the manuscript in the Data availability section.

Reviewer #6 (Remarks to the Author):

Overall, this is a well-designed and carefully executed study that provides valuable metabolic insight into Collodaria–algal symbiosis. The combination of stable isotope labeling and metabolomics is particularly effective for resolving carbon assimilation and transfer pathways within the holobiont.

However, while the findings are clearly presented and advance our understanding of Collodaria specifically, the broader patterns reported here—namely, the transfer of photosynthetically derived carbohydrates from algal symbionts to the host and the partitioning of phototrophic versus heterotrophic contributions to host metabolite pools—are well documented across many marine photosymbioses, including corals, jellyfish, foraminifera, and bivalves. In this sense, the results support and extend already established concepts rather than revealing fundamentally novel mechanisms of symbiont–host interaction.

That said, the study still fills an important gap by applying these approaches to Collodaria, a group for which such metabolic data are limited, and it provides a valuable foundation for future work on the ecological role and biogeochemical significance of radiolarian photosymbioses.

The manuscript would benefit from additional attention to the strength and framing of several conclusions, particularly where the results reiterate patterns already well established across other marine photosymbioses. In addition, there are numerous grammatical and phrasing issues throughout the text that hinder clarity. Addressing these points will substantially improve the precision, readability, and overall impact of the manuscript.

We thank the referee for the assessment of the manuscript and the helpful comments. Even if data on corals, jellyfish etc. exist, our study that introduces a novel methodology is the first to provide such an in depth insight into radiolaria photosymbiosis that was previously not accessible for these unculturable organisms.

Here are my more specific comments:

Line 44-45 - This sentence is a bit odd and doesn't really fit into the narrative of the paragraph – we fully agree and rephrased it to: "Despite their substantial role in primary production and the marine food web, the mechanisms governing photosymbiotic relationships remain poorly understood. This hampers our ability to reliably understand and predict the functioning of marine ecosystems and the impacts of climate change. The urgent need to better investigate the marine ecosystems and their biodiversity has been highlighted by many international initiatives, such as the 2025 United Nations Ocean Conference." – Lines 46-53.

Line 91 - Do you mean to say 'about the metabolites involved' rather than the involved metabolites? – corrected

Line 94 - Perhaps ive never heard this term before and stand corrected if it is a common term I'm unfamiliar with, but what do you mean by involved metabolites? Do you mean metabolites involved in a particular process of interest? If this is so, I think this needs to be a bit clearer and explained in each instance. – Thank you for this comment, we clarified the wording of the sentence to "… metabolites involved in biochemical processes" Line 139.

Line 96 - You identify involved metabolites, do you mean bioactive metabolites? – the analytical method does not allow to identify bioactive metabolites but covers a broad range of compounds. We rephrased this part to "By analyzing ^{13}C incorporation into the holobiont's endometabolome (the complete set of low-molecular weight metabolites within the holobiont), we identify the

specific metabolites involved in the photosymbiosis and determine the contribution of each mechanism of metabolite acquisition to the holobiont's metabolic pool." Lines 148-152.

Line 97 - Perhaps a typo here. I think you mean to say 'this allows comparison….' Or this method allows us to compare…..' – corrected

Line 102 - Why is DMSP a 'specialised metabolite' ? DMSP is very common among many bacteria and photosynthetic single cell organisms. – "specialized metabolites" is a term that distinguishes the compounds from those of primary metabolism. These compounds are also known as secondary metabolites. We now explain this term: "(i.e. compounds that are not directly required for basic growth, development, or reproduction, but instead serve ecological and adaptive functions)" Lines 113-115.

Line 144 - Here you mention 'holobiont' do you mean that here you're measuring the metabolites deriving from all organisms e.g. bacteria, photosymbiotic algae and collodarians? If that is the case, perhaps just define once that you're refering to all components. – Yes, term "holobiont" refers to the Collodaria together with the photosymbionts and associated bacteria, since the Collodaria were collected from the natura environment and didn't undergo any antibiotic treatments. Before incubation with labeled substate, we incubated organisms in filtered seawater to allow them to clean from attached particles including organisms, and prior to sample preparation, we checked each organism under the stereomicroscope, to be sure there no major contamination of any attached particles and organisms. We explain the term "holobiont" in the beginning of Results part (Line 160).

Line 145 - Here you can just say 'after 12h of light incubation' - corrected

Line 148 - Typo here, I think you mean to say 'to a minor extent' - corrected

Line 149 - I would continue to refer it as the collodarian holobiont or collodaria holobiont, not just holobiont. – Thank you for this suggestion, we added "Collodaria" to holobiont when appropriate

Line 163 - Remove 'a' from a significantly lower, just say 'resulted in significantly lower' - corrected

Line 169 - Should read 'dark incubations' as I imagine there was replication and not just one. - corrected

Line 171 - Typo: Should be 'alone' not along - corrected

Line 180, 165 etc. - Remove statistically from statistically significantly as this doesn't make sense. You can say statistically significant, but not in the way you've written this sentence. Just say 'significantly' throughout. Significant implies that it was statistically significant. - corrected

Line 181 - What two previous setups? Best to just briefly explain what you are referring to here. Also, please change throughout 'statistically significantly' as this doesn't make sense. In this case here, you can just say: The DOL of Ribose was significantly higher in comparison…. – with "two previous setups" we refer to 12h light incubation and 12h light/12h dark incubation. We corrected the text accordingly - lines 269, 274.

Line 185 - Again explain these setups briefly as its not clear.

This is not clear what you mean here. The photosynthetically derived carbonate showed an increase where? Compared to the dark treatment? This is not at all clear to me what you're comparing to here. – we clarified this part accordingly: "Furthermore, 24 h incubation with labeled bicarbonate resulted in label detection in stachydrine and palmitoyl-glycero-phosphocholine that were not labeled in both 12 h light and 12 h light/12 h dark incubations. The increase in the proportion of photosynthetically derived carbon after prolonged (24 h) light incubation compared to shorter light incubation (12 h) suggests a direct relationship between symbionts' photosynthetic activity and carbon incorporation in Collodaria holobiont metabolism." Lines 272-279.

Discussion

Line 241 –'this could be achieved' this should be in past tense and should read: 'This was achieved' - corrected

Line 243 – same here. Should read 'This approach allowed us' - corrected

Line 244 - I really think this is unlikely that DMSP originates from osmotrophy. The abundance of DMSP produced by the algal symbiont would likely far outweigh anything that is up taken from the surrounding environment. I understand you have detected labelled DMSP originating from the lysate, but I'm sure you must have detected it at much higher concentrations from the symbiotic collodaria also? – We detected label incorporation in DMSP after incubation with labeled bicarbonate, but only to a very minor extent compared to the uptake of labeled lysate. Our results confirm the dominant contribution of osmotrophy (see Fig. 2 and Fig. 3).

Ok after reviewing figure 3 further, I can see that the labelled portion of DMSP is much higher from the lysate labelled fractions. This is an interesting finding indeed. My question is though, how dense were these labelled lysate cultures? For example do you think the amount of lysed cells was more than what would be actually present within the actual collodaria cells? Is there any way you could show this? It would be very useful if this information was provided. See also comment to Referee 5 – we included these aspects in the text: "The concentration of the lysate was selected to correspond to cell counts of the algae that are ten to a hundred times lower than the symbiont density within a colony (Swanberg and Harbison 1980, Mansour et al. 2021) and the density of an exponentially growing algal culture, respectively." Line 289-292.

Line 255 - Is this really new information here? Isn't it already quite well established that photosymbionts provide hosts with glucose and in some cases fructose. Is this the first time it has been shown in Collodaria? I would say that this is highly likely. Although it is well-established for some systems, as we also indicate in introduction and discussion part, for Collodaria, to the best of our knowledge, there were no previous reports of glucose and fructose being involved in photosynthate assimilation. As we indicated in the previous sentence and in the introduction, in experiments that investigated photosynthate translocation, the composition of aqueous-soluble fraction was not determined. Therefore, although been intuitively the most expected mechanism, it has never been described for Collodaria. We rephrased this part to make the statement clearer: "Particularly, we observed that a substantial portion of glucose and fructose in the Collodaria holobiont was ^{13}C-enriched after incubation with labeled bicarbonate. An accumulation of photosynthetically derived glucose by the host has been previously reported for various

photosymbiont-bearing corals, jellyfish, and giant clams based on evaluations of ^{13}C or ^{14}C incorporation into host tissues after incubation with labeled bicarbonate (Hofmann and Kremer 1981, Ishikura et al. 1999, Whitehead and Douglas 2003, Burriesci et al. 2012, Matthews et al. 2018). Assimilation of photosynthetically derived fructose was previously reported for a photosymbiotic sea anemone (Matthews et al. 2018). Based on the earlier observations of substantial ^{14}C label incorporation in the aqueous-soluble fraction in central capsules of photosymbiont-bearing of Collodaria (Anderson et al. 1983), we conclude that in Collodaria photosymbiosis, photosynthetically assimilated glucose and fructose are metabolites that photosymbionts provide to the host." Lines 403-413.

Line 265 - need to restructure the sentence here and remove the full stop between sea anemones and the word 'and' . Sentences rarely start with the word 'and' – corrected

Line 268 - I think this is a somewhat weak conclusion, as this is already common knowledge in many other symbiotic relationships. If anything, you would need to convince the reader why this might be an interesting finding within Collodaria. Why would we presume otherwise that this would or would not be the case? – We rephrased our conclusions to emphasize the importance of the findings: "Together with glucose and fructose, we also observed a substantial DOL for putatively identified deoxyinositol, and myo- and scyllo-inositols upon incubation with labeled bicarbonate (Fig. 3). Inositols are cyclic sugar alcohols found in eukaryotic cells, including marine microalgae and protists. They function as compatible solutes to protect cells from osmotic stress and inositol phosphates mediate cellular signaling (Gillaspy 2011, Downing et al. 2018). In photosymbiotic systems, photosynthetically derived inositols were previously detected in the tissues of photosymbiotic sea anemones, where the presence of myo-inositol depended on the species of colonizing symbiont. The roles of these inositols, however, were not defined (Burriesci et al. 2012, Matthews et al. 2018). It was hypothesized, that they play a role in host-photosymbiont signaling, recognition and dysfunction, but a prove was not documented (Rosic et al. 2015, Matthews et al. 2018). Presence of photosynthetically-derived inositols in Collodaria photosymbiosis indicate that there could be a general mechanism of host-symbiont signaling in photosymbiotic systems involving dinoflagellates. Drawn parallels provide a direction for further investigations in host-symbiont communication in marine photosymbiotic systems."– Lines 414-428

Line 428 typo – should read 13C labeled - corrected

Line 429 typo- should read 'cultures' - corrected

Line 437 - This sentence doesn't read well. I think you mean to say something like 'To ensure residual lysate was removed…..' . - corrected

Line 449 - You might want to rephrase this to something like; 'and 1 μL from each sample was combined to create the pooled quality-control (QC) sample'. - corrected

Line 455 - Should read 'incubated at room temperature' spitted? Do you mean split? - corrected

Figure 3. Black and white figures are unnecessary these days as all journals are printed in colour or found online in colour. I would recommend making this figure more visually stimulating by adding some colour to it. – we thank the reviewer for the comment, however, from our point of view, the grey-scale graphs in the figure 3 don't lose any information if compared to the colorized

option, and are color vision deficiency friendly and ensure the translation of the information even if printed with the black/white option.

References

Anderson, O.R. 1976. Ultrastructure of a colonial radiolarian Collozoum inerme and a cytochemical determination of the role of its zooxanthellae. *Tissue and Cell*. 8:195–208.

Anderson, O.R., Swanberg, N.R. & Bennett, P. 1983. Assimilation of symbiont-derived photosynthates in some solitary and colonial radiolaria. *Marine Biology*. 77:265–9.

Balch, W.M., Drapeau, D.T., Poulton, N., Archer, S.D., Cartisano, C., Burnell, C. & Godrijan, J. 2023. Osmotrophy of dissolved organic compounds by coccolithophore populations: Fixation into particulate organic and inorganic carbon. *Science Advances*. 9.

Biard, T., Stemmann, L., Picheral, M., Mayot, N., Vandromme, P., Hauss, H., Gorsky, G. et al. 2016. In situ imaging reveals the biomass of giant protists in the global ocean. *Nature*. 532:504–7.

Bullock, H.A., Luo, H. & Whitman, W.B. 2017. Evolution of dimethylsulfoniopropionate metabolism in marine phytoplankton and bacteria. *Frontiers in Microbiology*. 8:637.

Burriesci, M.S., Raab, T.K. & Pringle, J.R. 2012. Evidence that glucose is the major transferred metabolite in dinoflagellate-cnidarian symbiosis. *Journal of Experimental Biology*. 215:3467–77.

Caron, D.A., Michaels, A.F., Swanberg, N.R. & Howse, F.A. 1995. Primary productivity by symbiont-bearing planktonic sarcodines (Acantharia, Radiolaria, Foraminifera) in surface waters near Bermuda. *Journal of Plankton Research*. 17:103–29.

Decelle, J. & Not, F. 2015. Acantharia. *In ELS*. John Wiley & Sons, Ltd., Chichester, pp. 1–10.

Decelle, J., Probert, I., Bittner, L., Desdevises, Y., Colin, S., Vargas, C. De, Galí, M. et al. 2012. An original mode of symbiosis in open ocean plankton. *Proceedings of the National Academy of Sciences of the United States of America*. 109:18000–5.

Decelle, J., Veronesi, G., LeKieffre, C., Gallet, B., Chevalier, F., Stryhanyuk, H., Marro, S. et al. 2021. Subcellular architecture and metabolic connection in the planktonic photosymbiosis between Collodaria (radiolarians) and their microalgae. *Environmental Microbiology*. 23:6569–86.

Downing, A.B., Wallace, G.T. & Yancey, P.H. 2018. Organic osmolytes of amphipods from littoral to hadal zones: Increases with depth in trimethylamine N-oxide , scyllo-inositol and other potential pressure counteractants. *Deep-Sea Research Part I*. 138:1–10.

Fettke, J., Hejazi, M., Smirnova, J., Höchel, E., Stage, M. & Steup, M. 2009. Eukaryotic starch degradation: Integration of plastidial and cytosolic pathways. *Journal of Experimental Botany*. 60:2907–22.

Ford, C.M., Sweetman, C. & Fry, S.C. 2024. Ascorbate degradation: Pathways, products, and possibilities. *Journal of Experimental Botany*. 75:2733–9.

Gillaspy, G.E. 2011. The cellular language of myo- inositol signaling. 823–39.

Gutierrez-Rodriguez, A., Pillet, L., Biard, T., Said-ahmad, W., Amrani, A. & Not, F. 2017. Dimethylated sulfur compounds in symbiotic protists: A potentially significant source for

marine DMS (P). *Limnology and Oceanography*. 62:1139–54.

Hofmann, D.K. & Kremer, B.P. 1981. Carbon metabolism and strobilation in Cassiopea andromedea (Cnidaria: Scyphozoa): Significance of endosymbiotic dinoflagellates. *Marine Biology*. 65:25–33.

Ishikura, M., Adachi, K. & Maruyama, T. 1999. Zooxanthellae release glucose in the tissue of a giant clam, Tridacna crocea. *Marine Biology*. 133:665–73.

Jackson, R. & Gabric, A. 2022. Climate change impacts on the marine cycling of biogenic sulfur: A review. *Microorganisms*. 10:1581.

Kanehisa, M. & Goto, S. 2000. KEGG : Kyoto Encyclopedia of Genes and Genomes. *Nucleic Acids Research*. 28:27–30.

Kirst, G.O., Thiel, C., Wolff, H., Nothnagel, J., Wanzek, M. & Ulmke, R. 1991. Dimethylsulfoniopropionate (DMSP) in icealgae and its possible biological role. *Marine Chemistry*. 35:381–8.

Klages, K., Boldingh, H. & Smith, G.S. 1999. Accumulation of myo-inositol in Actinidia seedlings subjected to salt stress. *Annals of Botany*. 84:521–7.

Kubiak, J.Z. & Kloc, M. 2018. Marine organisms as model systems in biology and medicine. Springer, Cham. 627 pp.

Lampitt, R.S., Briggs, N., Cael, B.B., Espinola, B., Hélaouët, P., Henson, S.A., Norrbin, F. et al. 2023. Deep ocean particle flux in the Northeast Atlantic over the past 30 years: Carbon sequestration is controlled by ecosystem structure in the upper ocean. *Frontiers in Earth Science*. 11:1176196.

LeKieffre, C., Jauffrais, T., Geslin, E., Jesus, B., Bernhard, J.M., Giovani, M.-E. & Meibom, A. 2018. Inorganic carbon and nitrogen assimilation in cellular compartments of a benthic kleptoplastic foraminifer. *Scientific Reports*. 8:10140.

Mansour, J.S., Norlin, A., Llopis Monferrer, N., L'Helguen, S. & Not, F. 2021. Carbon and nitrogen content to biovolume relationships for marine protist of the Rhizaria lineage (Radiolaria and Phaeodaria). *Limnology and Oceanography*. 66:1703–17.

Matthews, J.L., Oakley, C.A., Lutz, A., Hillyer, K.E., Roessner, U., Grossman, A.R., Weis, V.M. et al. 2018. Partner switching and metabolic flux in a model cnidarian–dinoflagellate symbiosis. *Proceedings of the Royal Society B: Biological Sciences*. 285:20182336.

Nikitashina, V., Bartels, B., Mansour, J.S., LeKieffre, C., Decelle, J., Hertweck, C., Not, F. et al. 2025. Metabolic interdependence and rewiring in radiolaria-microalgae photosymbioses. *The ISME Journal*. 19:wraf047.

Nikitashina, V. & Pohnert, G. 2023. Adaptation to salinity changes by phytoplankton: reassessing compositional changes of osmolytes in the light of modern analytical methods. *Cahiers de Biologie Marine*. 64:95–103.

Ohtsuka, S., Suzaki, T., Horiguchi, T., Suzuki, N., Not, F., Decelle, J., Colin, S. et al. 2015. Marine Protists. 1st ed. Springer Japan, Tokyo. 165–500 pp.

Probert, I., Siano, R., Poirier, C., Decelle, J., Biard, T., Tuji, A., Suzuki, N. et al. 2014. Brandtodinium gen. nov. and B. nutricula comb. Nov. (Dinophyceae), a dinoflagellate commonly found in symbiosis with polycystine radiolarians. *Journal of Phycology*. 50:388–

99.

Raina, J.-B., Tapiolas, D.M., Forêt, S., Lutz, A., Abrego, D., Ceh, J., Seneca, F.O. et al. 2013. DMSP biosynthesis by an animal and its role in coral thermal stress response. *Nature*. 502:677–80.

Rosic, N., Ling, E.Y.S., Chan, C.K.K., Lee, H.C., Kaniewska, P., Edwards, D., Dove, S. et al. 2015. Unfolding the secrets of coral-algal symbiosis. *ISME Journal*. 9:844–56.

Stincone, A., Prigione, A., Cramer, T., Wamelink, M.M.C., Campbell, K., Cheung, E., Olin-Sandoval, V. et al. 2015. The return of metabolism: Biochemistry and physiology of the pentose phosphate pathway. *Biological Reviews*. 90:927–63.

Stoecker, D., Johnson, M., DeVargas, C. & Not, F. 2009. Acquired phototrophy in aquatic protists. *Aquatic Microbial Ecology*. 57:279–310.

Sunda, W.G., Kieber, D., Kiene, R.P. & Huntsman, S. 2002. An antioxidant function of DMSP and DMS in marine algae. *Nature*. 418:317–20.

Swanberg, N.R. & Anderson, O.R. 1985. The nutrition of radiolarians: Trophic activity of some solitary Spumellaria. *Limnology and Oceanography*. 30:646–52.

Swanberg, N.R. & Harbison, G.R. 1980. The ecology of Collozoum longiforme, sp. nov., a new colonial radiolarian from the equatorial Atlantic Ocean. *Deep Sea Research Part A. Oceanographic Research Papers*. 27:715–32.

Swanberg, N.R.R. & Caron, D.A. 1991. Patterns of sarcodine feeding in epipelagic oceanic plankton. *Journal of Plankton Research*. 13:287–312.

Thamatrakoln, K. & Kustka, A.B. 2009. When to say when: Can excessive drinking explain silicon uptake in diatoms? *BioEssays*. 31:322–7.

Tunçay, H., Findinier, J., Duchêne, T., Cogez, V., Cousin, C., Peltier, G., Ball, S.G. et al. 2013. A forward genetic approach in Chlamydomonas reinhardtii as a strategy for exploring starch catabolism. *PLoS ONE*. 8.

Whitehead, L.F. & Douglas, A.E. 2003. Metabolite comparisons and the identity of nutrients translocated from symbiotic algae to an animal host. *Journal of Experimental Biology*. 206:3149–57.

Yancey, P.H. 2005. Organic osmolytes as compatible, metabolic and counteracting cytoprotectants in high osmolarity and other stresses. *Journal of Experimental Biology*. 208:2819–30.

Yost, D. & Mitchelmore, C. 2009. Dimethylsulfoniopropionate (DMSP) lyase activity in different strains of the symbiotic alga Symbiodinium microadriaticum. *Marine Ecology Progress Series*. 386:61–70.

Zettler, L.A., Sogin, M.L. & Caron, D.A. 1997. Phylogenetic relationships between the Acantharea and the polycystinea: A molecular perspective on Haeckel's radiolaria. *Proceedings of the National Academy of Sciences of the United States of America*. 94:11411–6.

To the editor

During revision our manuscript has gained a broader perspective so that we would suggest to adjust the title to: "Metabolic Partitioning between Photosynthesis and Osmotrophy in Collodaria Photosymbiotic Holobionts".

REVIEWER COMMENTS

We sincerely thank the reviewers for their thorough and insightful assessment of our manuscript. Their constructive comments and suggestions have been valuable in improving the final open points, and overall quality of the work. We have carefully considered all points raised and have revised the manuscript accordingly. In the following, we provide a detailed, point-by-point response to each comment and indicate the corresponding changes made in the revised version of the manuscript.

Reviewer #1 (Remarks to the Author):

The authors have done an appropriate job clarifying their study organism and research question in the introduction, and the manuscript is more defined and clear. The results now support the revised, more modest claim, and remove mention of carbon products being trafficked between holobiont partners. However, as other reviewers also pointed out, it boils down to a study examining the metabolites shared by photosymbionts with their radiolarian host.

Line 387 highlights their main findings: "Although our approach did not allow us to distinguish between host- and symbiont-derived metabolites within the holobiont, the comparatively high proportion of labeled carbohydrates, and relatively small contribution of the photosymbionts to the holobiont biomass, suggests that the detected carbohydrates were present in both the symbionts and the host. Therefore, based on our observations we hypothesize the following Collodaria–photosymbiont metabolic interactions: During photosynthesis, algae assimilate carbon and transfer it to the host as glucose and fructose, which are predominantly used for respiration to generate energy."

It is well established that photosymbionts will produce photosynthesis/energy products and provide them to their host, and it's also well understood that many protists engage in osmotrophy. Although interesting to detail this metabolic transfer, and valuable to the study of radiolarian ecology and physiology, the results will not necessarily be surprising to those outside the field. This work may therefore be more appropriate in a discipline-specific journal.

We appreciate the reviewer's perspective that metabolic exchanges in photosymbioses are, in general terms, well established. However, we would like to clarify that the main contribution of our study is entangling both phototrophic and osmotrophic contribution in the ecologically significant planktonic photosymbiosis of Collodaria. No comprehensive investigation of Collodaria nor related Radiolaria has been reported to date and generalizing findings from other photosymbioses to such a unique yet ecologically important system is, in our view, not justified. Investigation of different photosymbiotic systems and comparison of common and unique mechanisms will provide a better understanding of general and specialized mechanisms underlying the establishment and evolution of photosymbiotic systems.
As outlined in the final paragraph of the manuscript, our work expands current understanding of the holobiont's role in oceanic elemental cycles. In the context of global environmental change, elucidating the metabolic contributions of such keystone organisms will improve our ability to predict how these systems and planktonic photosymbioses more broadly influence

marine nutrient cycling..In line with these arguments, we rephrased parts of the introduction and discussion.

Reviewer #2 (Remarks to the Author):

The authors of the manuscript "Partitioning of Carbon Metabolism Between Photosynthesis and Osmotrophy in Collodaria Photosymbiotic Holobionts" use isotope labelling and mass spectrometry to perform a metabolomic analysis on the Collodaria holobiont. The authors found that, in the presence of light, labeled glucose and fructose are produced, which are then catabolized in the dark. Longer incubations in the light led to increased labeling of photosynthate as well as labeling of the catabolism products. Meanwhile, labeled osmolytes such as DMSP were detected predominantly in the labeled algal lysate treatment. The work convincingly demonstrates that photosymbionts are photosynthetically active and contributing sugars to the Collodaria holobiont. This concrete evidence is a welcome contribution to the study of the Radiolaria photosymbiosis.
The revised version is substantially improved. Our major concerns were that the specific aims and rationale for each experiment were not sufficiently clear, the methods were not explained to a broad audience, and the results were not interpreted in the context of radiolarian biology or previous research. In this version, the rationale for each experiment in the list is clearer and more accurately expressed, and the additional information in the results section is helpful. The methods have less jargon and are easier to understand.
However, while there was improvement in placing the results in the context of radiolarian biology, such as adding the mention of phagotrophy to the introduction, there is still room for improvement in this area. Specifically, the authors have argued in their rebuttal that they demonstrated that there is no particle-associated carbon that could have been phagocytosed during the experiment. This may be true, but it is a separate question from our concern, which is that phagotrophy is certainly biologically relevant to Collodaria in the ocean while osmotrophy may or may not be. This should be emphasized more clearly throughout the "Contribution of osmotrophy to Collodaria metabolism" section, and in the discussion (~lines 311-312, 393-394) that the sources of these labeled compounds in this experiment might not reflect the important sources for Collodaria in the ocean, because phagotrophy could not be tested here.

We acknowledge the reviewer's point that phagotrophy has been demonstrated in Collodaria, whereas osmotrophy has not.
However, Swnaberg and Anderson question the general relevance of phagotrophy (Swanberg and Anderson 1985). They reported that several tested Radiolaria generally did not respond to feeding regimes. If they were illuminated, the mean survivorship of starved specimens was the same as that of those fed herbivorous, carnivorous, or omnivorous diets. This suggests that phagotrophy is probably important but not essential for the survival of these photosymbiotic systems.
On the other hand, dissolved organic compounds represent a major reservoir of organic carbon in the ocean (Thornton 2014). They are passively or actively released by phytoplankton and the release is high in oligotrophic waters (Thornton 2014) where Collodaria are abundant. Therefore, assimilation of dissolved organic compounds from the surrounding environment may be relevant for Collodaria. This contribution was tested in the current study.
These aspects are now clearly mentioned in the manuscript. We added information about the significance of dissolved organic compounds in the ocean, to rationalize our choice for

investigation of osmotrophy. We also added information that all experiments were conducted and interpreted in the absence of phagotrophy as suggested by reviewer.

Additional Comments:

Introduction

Line 51: Consider adding “For example,” before “In some sea slugs” because otherwise the preceding sentence sounds as if it is missing a citation. - corrected

Lines 51-54: All of the references here are to photosymbioses in animals. It would be more relevant to cite research on photosymbioses in protists. In particular, the sea slug is probably not the beset example because in sea slugs it is doubtful whether the plastids remain active or transfer sugars to the host.

– we added information and references for photosymbiotic ciliates and foraminifera (Lines 52-58): *“For example, in some sea slugs with acquired photosynthetic plastids, the photosynthesis provides less than 1% of the total organic carbon, whereas in some pelagic foraminifera, ciliates, corals, and jellyfish, the photosynthetic activity of symbionts can almost fully meet the carbon requirements (Esteban et al. 2010, Johnson 2011, Rauch et al. 2017, Melo Clavijo et al. 2018, Djeghri et al. 2019). The form of the translocated photosynthetic carbon includes diverse metabolites like lipids in foraminifera, sugars in ciliates, glycerol, lipids, and glucose in corals, or carbohydrates, fatty acids, and amino acids in jellyfish (Hofmann and Kremer 1981, Yellowlees et al. 2008, Mortillaro et al. 2009, Esteban et al. 2010, Johnson 2011, Ohtsuka et al. 2015, Kubiak and Kloc 2018, LeKieffre et al. 2018a, Djeghri et al. 2019).”*

Lines 63-67: Please reconsider these sentences because it is unclear and seems speculative and perhaps even incorrect. Whether or not radiolaria have mechanisms to take up dissolved organic matter was not directly tested in this study.

– We agree with the reviewer that the information provided here was not fitting to the context – We deleted this part to avoid any misinterpretation.

The results suggest that certain organic compounds accumulate in the holobiont, but they could be accumulated by the algae, not the radiolaria, or the accumulation could be passive, perhaps by adherence to the extracellular matrix. Furthermore, the terms “organic carbon” and “nutrients” in this context are vague and potentially misleading. The results of this study suggest that photosymbionts fix carbon that is then respired, but the organic compounds taken up are not likely a significant energy source. Therefore the terms “vitamins” or “micronutrients” might be more relevant than “nutrients”, but again direct evidence is lacking.

We do demonstrate assimilation of metabolites by the holobiont. Even if accumulated by the algae, as the reviewer suggested, the compounds must still pass the host cell membrane because the algal symbionts are present exclusively inside host cells. Concerning their adherence to the extracellular matrix, the probability of this is very low because the organisms were washed three times with the total incubation time of 2 hours in the filtered non-labeled seawater, before sample preparation. Since the detected compounds are very polar, they will readily dissolve in the water rather than adhere to the cell membrane. In addition, the majority of these compounds were previously detected inside the Collodaria holobiont (Nikitashina et al. 2025).

Finally, none of the works cited here support the suggestion that heterotrophic (and/or mixotrophic) protists engage in osmotrophy. These sentences distract from the more important point at the beginning of the paragraph, which is that radiolarians are known to ingest large

prey and this is therefore likely a very important source of nutrients, at least equal to photoautotrophy, and remains an open question as to its nutritional contribution relative to the photosymbionts. Whether radiolarian cells engage in osmotrophy is currently speculative.

Cited references include information about heterotrophic protists: in Ohtsuka et al. 2015, the osmotrophy is described among others for Chytridiomycetes, heterotrophic flagellates, non-photosynthetic euglenids, hetrotrophic archaeplastids,; in Weisse 2017 the osmotrophy is mentioned in ciliates; Mitra et al. 2014, and Flynn et al. 2019 suggest that osmotrophy is ubiquitous for both phyto- and microzooplankton.

Line 71: The cited source here is not relevant for the stated information.
– We disagree since the cited source (Biard et al. 2016) reports that "…in the top 100 m of the water column, photosymbiotic Collodaria contributed most to rhizarian biomass…" Since Collodaria are a subgroup of radiolarians within Rhizaria, this supports our statement that photosymbiotic Collodaria are the most abundant radiolaria in the upper 100 m of the water column. For clarity, we refer specifically to radiolarians to make the statement more accessible to readers who may not be familiar with rhizarian phylogeny.

Lines 87-88: Please add a citation for "an earlier study".
– added

Lines 104-109: Please mention how DMSP is important to algal cell metabolism in addition to describing its importance to the sulfur cycle.
– we added this information (Line 109): It serves as osmoprotectant, antioxidant, and signaling molecule (Sunda et al. 2002, Raina et al. 2013, Nikitashina and Pohnert 2023).

Line 112: Correct the strange typographical error "12 h" - corrected

Line 113: Remove comma after "both" - corrected

Lines 126-128: It seems a bit facetious to state that there was a deliberate focus on osmotrophy when the truth is that osmotrophy might or might not be biologically relevant for radiolaria. It is certainly less relevant than phagotrophy. Perhaps a more nuanced justification, acknowledging that phagotrophy is more important in life but less experimentally feasible, for example, would be more appropriate here
– thank you for pointing this out, we added the information about significance of dissolved organic compounds in the ocean to clarify our approach (Lines 113-119): *"Dissolved organic compounds represent a major reservoir of organic carbon in the ocean (Thornton 2014). They are passively or actively released by phytoplankton and play an essential role in global carbon cycling being a primary carbon source for marine pelagic prokaryotes (Thornton 2014). Furthermore, various protists are capable of uptake and consumption of dissolved organic compounds in a process termed osmotrophy (Mitra et al. 2014, Ohtsuka et al. 2015, Selosse et al. 2017, Weisse 2017, Flynn et al. 2019, Meyer et al. 2022). Osmotrophy has been mainly studied in phytoplankton (Vila-Costa et al. 2006, Ruiz-González et al. 2012, Beamud et al. 2014, Balch et al. 2023), but has not yet been observed for Collodaria (Biard et al. 2015)."*

Furthermore, we rephrased the paragraph to be more precise (Lines 130-139): *"Incubations with labeled inorganic and organic carbon sources thus allow us to distinguish between phototrophic and osmotrophic metabolite acquisition in the colonial Collodaria photosymbiosis in the absence of phagotrophy. By analyzing ^{13}C incorporation into the holobiont's endometabolome (the complete set of low-molecular weight metabolites within the holobiont),*

we identify the specific metabolites involved in the photosymbiosis and determine the contribution of photosynthesis and osmotrophy to metabolite acquisition of holobiont."

Results

Line 135: The statement "we were able to elucidate the contribution of the respective partners" sounds somewhat inappropriate in tone because at this point the results have not yet been presented. How about "we aimed to elucidate"? It would make better grammatical sense with "we therefore" later in the sentence. – changed accordingly

Lines 244-247: Please reword to be clearer about the lysate concentration. Does this sentence mean that the concentration was ten times lower than symbiont density and 100 times lower than an algal culture? Or does it mean 10-100x lower than various measurements for symbiont and algal culture density?
– Revised (Lines 255-259): *"The final lysate concentration corresponded to algal cell densities that were approximately 10 times lower than the symbiont density within a colony".*

Line 262: Please add "in this experiment" to the end of this sentence to avoid giving the impression that osmotrophy is a significant process in Collodaria in general, because phagotrophy is likely far more important but was not directly studied. We clarified this point (line 274)

Line 277: "synthetized" should be synthesized. - corrected

Discussion

Line 311: Please qualify this statement with "in the absence of phagotrophy" or "in this experiment, in the absence of phagotrophy"
- we fully agree and added the sentence (Line 325-326): *"It has to be considered that all experiments were conducted in the absence of phagotrophy that might provide additional resources."*

Lines 333-334: Please remove the comma after "hypothesized" and change "but a prove was not documented" to "but this has not been demonstrated"
– we introduced corrections accordingly (lines 346-348): *It was hypothesized, that they play a role in host-photosymbiont signaling, recognition and dysfunction, but no proof a prove was not documented*

Line 369: Another strange typo "12 h". - corrected

Line 369: "the total DOL was analytically not accessible" please explain/clarify this statement.
Now explained (Lines 389-390): *"In metabolomics studies the total DOL is not accessible but the DOL of the identified metabolites is recorded."*

Line 373: Change "in favor of the first mechanism speaks an increase in label incorporation" to "in favor of the first mechanism, there was an increase in label incorporation".
– we rephrased the sentence accordingly (lines 394-396): *"An increase in label incorporation for some metabolites, such as ribose, threonic acid, gonyol, and deoxyinositol during the dark period, supports the first mechanism."*

Lines 393-394: Please remind the reader that phagotrophy was not considered in this experiment.

– we rephrased the paragraph osmotrophy in accordance with this and following comment and added information justifying the choice of osmotrophy (Lines 417-428): "*Dissolved organic compounds constitute a significant portion of organic carbon in the ocean and their release by phytoplankton is high in oligotrophic waters (Thornton 2014). Since various protists are capable of osmotrophy (Ohtsuka et al. 2015), and since it has been suggested that the osmotrophy is ubiquitous among phyto- and microzooplankton (Mitra et al. 2014, Flynn et al. 2019), we investigated the ability of the Collodaria holobiont to assimilate dissolved organic compounds from the surrounding environment. Our results suggest that the Collodaria exhibit a selective uptake of defined metabolites. Thus, we did not observe any label incorporation for carbohydrates and amino acids when incubated with labeled dissolved organic compounds. This fact supports our conclusions above that carbohydrates are primarily derived from photosynthetic activity at least in the absence of phagotrophy.*"

Lines 396-401: Again, please be clear that these are not generalizable results to Collodaria in the ocean, but simply a demonstration of different carbon uptake mechanisms in this experiment, in the absence of phagotrophy. – please see the answer above

Lines 412-415: This concluding sentence is a bit awkwardly worded and seems to be making a wider generalization than is warranted by the data. The papers cited previously did not quantify carbon allocation by heterotrophy relative to photoautotrophy, only that light was essential and prey items were interchangeable.
– We focus now clearer on the core of our manuscript and deleted the corresponding lines.

Methods
Line 497: Remove strange symbol typo in "March". - corrected

Reviewer #2 (Remarks on code availability):
The code used for isotope analysis is beyond our expertise to review.

Reviewer #4 (Remarks to the Author):

I co-reviewed this manuscript with one of the reviewers who provided the listed reports. This is part of the Nature Communications initiative to facilitate training in peer review and to provide appropriate recognition for Early Career Researchers who co-review manuscripts.

Reviewer #5 (Remarks to the Author):

I thank the authors for their detailed responses and for considering all of my suggestions and comments. I confirmed that significant revisions were made to the manuscript according to my review, as well as the other reviewers'.
I have only a few additional minor comments.

-Host-symbiont signaling
In the manuscript, they mentioned the possible function of inositols as host-symbiont signaling, and this could be a general mechanism in photosymbiosis. In my understanding, general signaling molecules are e.g., lectins (C-type lectins). Although I understand that multiple

substances may play a role in signaling, and inositols may be the ones, this part of the discussion (L331–338) sounds simplistic. It requires a more holistic review of host-symbiont signaling, what is known in the other photosymbiosic systems.
– thank you for pointing this out, we revised this paragraph (line 348-356): *"Although observations suggest that signaling is active in many photosymbiotic associations, little is known about the involved chemistry (Rosset et al. 2021). Among the identified signals in corals are glycans, oxylipins, reactive species, noncoding RNAs, and also secondary messengers associated with the phosphatidylinositol pathway (Rosset et al. 2021, Tortorelli et al. 2022). Presence of photosynthetically-derived inositols in Collodaria photosymbiosis suggest a possible role in host-symbiont signaling. Drawn parallels provide a direction for further investigations in host-symbiont communication in marine photosymbiotic systems."*

L110: "12 h" at the top should be deleted. - corrected

L345-347: Please also cite LeKieffre et al. (2018, Marine Biology), which discussed lipid transfer from symbionts to the host in planktonic foraminifera photosymbiosis. Actually, the reference I cited in the previous review report was LeKieffre et al. (2018, Marine Biology), not LeKieffre et al. (2018, Scientific reports). The latter also addressed the lipid transfer, but for benthic foraminifera.
– we added the reference and the information accordingly (lines 363-365): *Furthermore, accumulation of translocated symbiotic photosynthates in the form of lipid droplets has been observed in planktonic foraminifera with dinoflagellate photosymbionts (LeKieffre et al. 2018b).*

L361: Threonic acid, in turn, is formed form from… ---> formed from - corrected

L369: "12 h" at the top should be deleted. - corrected

Reviewer #6 (Remarks to the Author):

I have reviewed the revised manuscript and am pleased to see that the authors have carefully and thoroughly addressed all of my previous comments. The revisions have strengthened the manuscript, and this great study now presents a clear and well-supported contribution to the field. I only have a small number of remaining grammatical and phrasing suggestions, which are provided below. Presuming these very minor changes are addressed, I will not need to see the manuscript again and can recommend it for publication in this journal.

Here are my very minor comments:

Line 17 – you can write just ' maintenance of photosymbiosis'- corrected

Line 31 – remove 'the' can be written as just 'fluxes in plankton'- corrected

Line 35 – Again, this sounds a little odd here because of the inanimate passion phrasing. Here's a suggestion to make the sentence flow better:' Phototrophic microorganisms inhabiting the upper layers of the water column form the foundation of the marine food web and contribute significantly to the global carbon cycle via photosynthesis'- corrected

Line 47 – change to 'suggests that photosymbionts provide organic compounds' - corrected

Line 78 maybe put ‘still’ or ‘currently’ not fully understood. - corrected

Line 86 – should be ‘products’ with an s. - corrected

Line 89 – ‘but other more polar metabiolites….’ - corrected

Line 110 – there seems to be some error with the beginning of the sentence here, I think the 12 should have been deleted. I also suggest to start the sentence with: ‘Here we address the origin….’ – rephrased accordingly

Line 112 – Suggestion: ‘ This method makes it possible to investigate…..’ – rephrased accordingly

Line 275 – sentence structure issues. ‘So that analyzed’ I’m unsure what you mean here, please address.
– rephrased to: “*These results imply slow biosynthesis of these metabolites in the holobiont, so such that analyzed incubation times used were not sufficient for accumulation of newly synthestized metabolites in detectable amounts neither by the photosymbionts nor by the host. (Lines 287-290).”*

Discussion
Line 308 – ‘both sugars were’ ? perhaps should be in past tense? - corrected

Line 313 – Particularly is a strange way to start a new paragraph. I suggest its replaced with ‘In particular’ or just ‘We observed…’ - corrected

Line 331 – I know I keep asking ‘the’ to be removed, but in this case this sentence would benefit from it. ‘depending on the species of the colonizing symbiont’. - corrected

Line 333 – grammatical error - ‘but no proof was documented’ - corrected

Line 351 – change to ‘significant decrease’- corrected

Line 361 – form has been typed twice here, please fix. - corrected

Line 369 – typo at beginning of paragraph, please check - corrected

Line 373 – speaks an increase? This doesn’t make sense, could you please rephrase here.
– rephrased to: *“An increase in label incorporation for some metabolites, such as ribose, threonic acid, gonyol, and deoxyinositol during the dark period, supports the first mechanism. (Lines 394-396).”*

Line 394 – remove ‘the’ you can put just Collodaria - corrected

Line 396 – Change ‘this fact’ to ‘this finding’- corrected

Line 401 - This sentence is a bit odd, I suggest a rephrase to something like this: All metabolites in this category exhibit high polarity, which may be a prerequisite for uptake, and all contain

either nitrogen or sulfur, suggesting that osmotrophic assimilation of these compounds may also contribute to nitrogen or sulfur acquisition. – rephrased accordingly

Line 443 – 'together with that' this is not correct phrasing here, please change. Do you mean to say 'collectively' ?
– we changed 'together with that' to "alongside this" which is in our opinion more fitting to the context (Line 476).

Line 445 – Do you mean to say? Through (or via) accumulation of high – corrected to "Through"

Line 448 – 'marine microbial communities' - corrected

Reviewer #6 (Remarks on code availability):

I have not reviewed the code as it falls outside of my expertise.

References

Balch, W.M., Drapeau, D.T., Poulton, N., Archer, S.D., Cartisano, C., Burnell, C. & Godrijan, J. 2023. Osmotrophy of dissolved organic compounds by coccolithophore populations: Fixation into particulate organic and inorganic carbon. *Science Advances*. 9:eadf6973 (2023).

Beamud, S.G., Karrasch, B., Pedrozo, F.L. & Diaz, M.M. 2014. Utilisation of organic compounds by osmotrophic algae in an acidic lake of Patagonia (Argentina). *Limnology*. 15:163–72.

Biard, T., Pillet, L., Decelle, J., Poirier, C., Suzuki, N. & Not, F. 2015. Towards an Integrative Morpho-molecular Classification of the Collodaria (Polycystinea, Radiolaria). *Protist*. 166:374–88.

Biard, T., Stemmann, L., Picheral, M., Mayot, N., Vandromme, P., Hauss, H., Gorsky, G. et al. 2016. *In situ* imaging reveals the biomass of giant protists in the global ocean. *Nature*. 532:504–7.

Djeghri, N., Pondaven, P., Stibor, H. & Dawson, M.N. 2019. Review of the diversity, traits, and ecology of zooxanthellate jellyfishes. *Marine Biology*. 166:1–19.

Esteban, G.F., Fenchel, T. & Finlay, B.J. 2010. Mixotrophy in Ciliates. *Protist*. 161:621–41.

Flynn, K.J., Mitra, A., Anestis, K., Anschütz, A.A., Calbet, A., Ferreira, G.D., Gypens, N. et al. 2019. Mixotrophic protists and a new paradigm for marine ecology: where does plankton research go now? *Journal of Plankton Research*. 41:375–91.

Hofmann, D.K. & Kremer, B.P. 1981. Carbon metabolism and strobilation in *Cassiopea andromedea* (Cnidaria: Scyphozoa): Significance of endosymbiotic dinoflagellates. *Marine Biology*. 65:25–33.

Johnson, M.D. 2011. The acquisition of phototrophy: Adaptive strategies of hosting endosymbionts and organelles. *Photosynthesis Research*. 107:117–32.

Kubiak, J.Z. & Kloc, M. 2018. Marine organisms as model systems in biology and medicine. Springer, Cham. 627 pp.

LeKieffre, C., Spero, H.J., Russell, A.D., Fehrenbacher, J.S., Geslin, E. & Meibom, A. 2018a. Assimilation, translocation, and utilization of carbon between photosynthetic symbiotic dinoflagellates and their planktic foraminifera host. *Marine Biology*. 165:1–15.

LeKieffre, C., Spero, H.J., Russell, A.D., Fehrenbacher, J.S., Geslin, E. & Meibom, A. 2018b. Assimilation, translocation, and utilization of carbon between photosynthetic symbiotic dinoflagellates and their planktic foraminifera host. *Marine Biology*. 165:1–15.

Melo Clavijo, J., Donath, A., Serôdio, J. & Christa, G. 2018. Polymorphic adaptations in

metazoans to establish and maintain photosymbioses. *Biological Reviews*. 93:2006–20.
Meyer, N., Rydzyk, A. & Pohnert, G. 2022. Pronounced uptake and metabolism of organic substrates by diatoms revealed by pulse-labeling metabolomics. *Frontiers in Marine Science*. 9.
Mitra, A., Flynn, K.J., Burkholder, J.M., Berge, T., Calbet, A., Raven, J.A., Granéli, E. et al. 2014. The role of mixotrophic protists in the biological carbon pump. *Biogeosciences*. 11:995–1005.
Mortillaro, J.M., Pitt, K.A., Lee, S.Y. & Meziane, T. 2009. Light intensity influences the production and translocation of fatty acids by zooxanthellae in the jellyfish *Cassiopea* sp. *Journal of Experimental Marine Biology and Ecology*. 378:22–30.
Nikitashina, V., Bartels, B., Mansour, J.S., LeKieffre, C., Decelle, J., Hertweck, C., Not, F. et al. 2025. Metabolic interdependence and rewiring in radiolaria-microalgae photosymbioses. *The ISME Journal*. 19:wraf047.
Nikitashina, V. & Pohnert, G. 2023. Adaptation to salinity changes by phytoplankton: reassessing compositional changes of osmolytes in the light of modern analytical methods. *Cahiers de Biologie Marine*. 64:95–103.
Ohtsuka, S., Suzaki, T., Horiguchi, T., Suzuki, N., Not, F., Decelle, J., Colin, S. et al. 2015. Marine Protists. 1st ed. Springer Japan, Tokyo. 165–500 pp.
Raina, J.-B., Tapiolas, D.M., Forêt, S., Lutz, A., Abrego, D., Ceh, J., Seneca, F.O. et al. 2013. DMSP biosynthesis by an animal and its role in coral thermal stress response. *Nature*. 502:677–80.
Rauch, C., Jahns, P., Tielens, A.G.M., Gould, S.B. & Martin, W.F. 2017. On being the right size as an animal with plastids. *Frontiers in Plant Science*. 8:1402.
Rosset, S.L., Oakley, C.A., Ferrier-Pagès, C., Suggett, D.J., Weis, V.M. & Davy, S.K. 2021. The molecular language of the cnidarian–dinoflagellate symbiosis. *Trends in Microbiology*. 29:320–33.
Ruiz-González, C., Galí, M., Sintes, E., Herndl, G.J., Gasol, J.M. & Simó, R. 2012. Sunlight effects on the osmotrophic uptake of DMSP-sulfur and leucine by polar phytoplankton. *PLoS ONE*. 7:1–14.
Selosse, M.A., Charpin, M. & Not, F. 2017. Mixotrophy everywhere on land and in water: The grand écart hypothesis. *Ecology Letters*. 20:246–63.
Sunda, W.G., Kieber, D., Kiene, R.P. & Huntsman, S. 2002. An antioxidant function of DMSP and DMS in marine algae. *Nature*. 418:317–20.
Swanberg, N.R. & Anderson, O.R. 1985. The nutrition of radiolarians: Trophic activity of some solitary Spumellaria. *Limnology and Oceanography*. 30:646–52.
Thornton, D.C.O. 2014. Dissolved organic matter (DOM) release by phytoplankton in the contemporary and future ocean. *European Journal of Phycology*. 49:20–46.
Tortorelli, G., Rautengarten, C., Bacic, A., Segal, G., Ebert, B., Davy, S.K., van Oppen, M.J.H. et al. 2022. Cell surface carbohydrates of symbiotic dinoflagellates and their role in the establishment of cnidarian–dinoflagellate symbiosis. *ISME Journal*. 16:190–9.
Vila-Costa, M., Simó, R., Harada, H., Gasol, J.M., Slezak, D. & Kiene, R.P. 2006. Dimethylsulfoniopropionate uptake by marine phytoplankton. *Science*. 314:652–4.
Weisse, T. 2017. Functional diversity of aquatic ciliates. *European Journal of Protistology*. 61:331–58.
Yellowlees, D., Rees, T.A. V. & Leggat, W. 2008. Metabolic interactions between algal symbionts and invertebrate hosts. *Plant, Cell & Environment*. 31:679–94.

Review of Vera Nikitashina and Georg Pohnert "Partitioning of Carbon Metabolism Between Photosynthesis and Osmotrophy in Collodaria Photosymbiotic Holobionts"

Overall comment: I enjoyed reading this ms. It is a very addition to many questions that were investigated ~50 years ago, but now using modern approaches. Aside from improving our knowledge of radiolarian ecology, it will be very useful for the understanding of symbioses overall. I do not have many criticisms about the methodologies used here; it is pretty straightforward, as the results. However, I was a bit frustrated with the discussion section. The authors mentioned many different elements, but only provided a glimpse of their impact on the holobiont functioning. I have made several specific comments below to indicate this lack of discussion. Finally, the introduction is lacking details, which can be easily added.
Overall, with a bit of polishing and editing, it will be a good and useful ms.

I made specific comments below.

Abstract

Line 15- I would write "can contribute". There are only a few reports of them contributing to primary production.

Introduction

Line 39- I would add a general reference to marine photosymbioses (perhaps remove one of the many references to photosymbioses with protists?).

Line 42- What is meant by 'central for the global photosynthesis budget'? The same goes for 'roles of photosymbiotic'? Please provide clearer elements.

Line 53- photosynthetic 'plastids'?
Line 53- 'carbon pool' = inorganic? Organic?

Line 58- The transition from the previous paragraph is odd. We are jumping from jellyfish to radiolarians?

Line 59- 'Four out of five orders'. Most readers will be ignorant about radiolarian classification. I would either name the five orders, mention the only non-mixotrophic order, and/or at least provide a reference for this statement.

Line 62- It is quite unusual to see mixotrophy mentioned only at this point. Mixotrophy should be mentioned earlier (in particular since you mention kleptoplastidy with your sea-slugs).

Line 63- Again, Collodaria appear out of nowhere! You should probably introduce them with one sentence before (at least say that it's one of the mixotrophic orders you mentioned before).

Line 64- radiolaria comes with a capital R.

Line 65- I would separate this sentence into two; it is pretty long and hard to follow.

Line 71- Is it really a matter of debate, or rather something that we do not know?

Line 75- ‘other studies’ Which one?!?!

Results

Figure 2. I am not used to seeing such graphics. There is no explanation about ‘mlz’? What does it stand for? Also, your y-axis says ‘relative abundance’. I am guessing that it is expressed as %. If so, why would you have several 100% bars? Please clarify the figure and the caption.

Line 126- What are ‘CL’ and ‘EL’ modes?

Line 132- You have defined before who ‘B. nutricula’ are (and cannot use the abbreviation then).

Line 176- This is the first time you mention the ‘metabolome’. You should define this above in the introduction. The same goes for ‘osmotrophy’ later.

Line 192- What is an ‘algal lysate’?

Line 208- Monitored for how long? I think that it is important to mention the incubation time since you are arguing that it was not enough to detect accumulation.

Discussion

Line 238- I would provide a reference to support ‘uncultivable’.

This first paragraph seems like a conclusion rather than the first paragraph of a discussion section (which would be the second).

Line 266- ‘role in host-photosymbiont signaling’. Unclear, be more precise.

Line 282- If ribose and deoxyinositol are continuously produced, please explain what roles do they play in the holobiont?. The same goes for your sentence at line 298.

Line 303- Technically speaking, you did not observe any feeding. You speculate that lysates were transferred to the host, but the mechanisms are unknown.

Line 316- I would turn your sentence saying that you support observations made for corals. I think that you can simply say that the explanations provided for coral symbioses could the same for Collodaria (and open the door to several common mechanisms in photosymbiosis overall).

Line 321- Here, you tested only heterotrophic feeding through the scope of osmotrophy. Are there any elements from past literature (e.g., Anderson and Swanberg’s experiments back in the 80s).

Line 322- What would be the interest for Collodaria to uptake DMSP? What is the role of DMSP in symbiosis? Was it not suggested to play an antioxidant role in radiolarian symbioses? Isn't DMSP produced by the symbionts and their production boosted by the host (see work from Decelle's lab) to cope with high light stress?
